# Nitrile-Based *Leishmania* Cysteine Protease B Inhibitors with In Vivo Efficacy against Visceral Leishmaniasis

**DOI:** 10.1021/acsomega.6c06826

**Published:** 2026-10-22

**Authors:** Jhonatan Santos de Lima, Michele Ferreira da Silva Mela, Juliana Romano Lopes, João Lucas Bruno Prates, Natalia Caroline Costa Coelho, Ana Clara Lunardi Yagi, Rafael Consolin Chelucci, Simone Michelan Duarte, Leonardo Luiz Gomes Ferreira, Adriano Defini Andricopulo, Gustavo Chagas Paulino, João Victor Marcelino de Souza, Bruna Cardinali Lustri Morgado, Paulo Ricardo da Silva Sanches, Eduardo Maffud Cilli, Jean Leandro dos Santos, Angela Maria Arenas Velásquez, Marcia Aparecida Silva Graminha

**Affiliations:** † Department of Clinical Analysis, School of Pharmaceutical Sciences, São Paulo State University (UNESP), Araraquara 14800-903, Brazil; ‡ Department of Drugs and Medicines, School of Pharmaceutical Sciences, São Paulo State University (UNESP), Araraquara 14800-903, Brazil; § Laboratory of Medicinal and Computational Chemistry, Physics Institute of São Carlos, University of São Paulo (USP), São Carlos 13563-120, SP, Brazil; ∥ Department of Biochemistry and Organic Chemistry, Institute of Chemistry, São Paulo State University (UNESP), Araraquara 14800-060, Brazil; ⊥ Department of Biological Sciences, School of Pharmaceutical Sciences, São Paulo State University (UNESP), Araraquara 14800-903, Brazil; # Latin American Institute of Life and Nature Sciences, Federal University of Latin American Integration (UNILA), Foz do Iguaçu 85870-650, Brazil

## Abstract

Leishmaniasis remains a major neglected tropical disease with limited therapeutic options and increasing concerns regarding toxicity and drug resistance. Cysteine protease B (CPB), a key virulence factor in *Leishmania* spp., represents an attractive molecular target for the development of new antileishmanial agents. In this study, we designed and synthesized a series of dipeptidyl nitrile derivatives as reversible covalent CPB inhibitors and evaluated their biological activity in vitro and in vivo. Molecular docking performed in covalent mode demonstrated favorable accommodation of the compounds within the CPB active site, with the benzyl-containing derivatives showing the most favorable interactions. Among the series, compound **1** ((*S*)-*tert*-butyl (1-((cyanomethyl)­amino)-1-oxo-3-phenylpropan-2-yl)­carbamate) exhibited potent inhibition of recombinant *Lm*CPB2.8ΔCTE (IC_50_ = 4.3 ± 0.2 μM) and strong activity against intracellular amastigotes of *Leishmania infantum* (EC_50_ = 3.9 ± 0.5 μM; SI ≈ 77) and *Leishmania amazonensis* (EC_50_ = 4.6 ± 1.6 μM; SI ≈ 65), surpassing miltefosine in selectivity. Compound **1** showed no cytotoxicity toward murine macrophages, no hemolytic activity, and good tolerability in the *Galleria mellonella* model. Oral administration of compound **1** to *L. infantum*-infected BALB/c mice (0.77 mg/kg/day for 5 days) resulted in a significant reduction in parasite burden in the liver and spleen (≈ 40–55%), even at a 10-fold lower dose than miltefosine. Although alterations in hepatic transaminases were observed, no evidence of renal dysfunction was detected. Collectively, these findings identify dipeptidyl nitriles as promising CPB-targeting scaffolds and position compound **1** as a lead structure for further optimization aimed at enhancing antileishmanial efficacy while minimizing host toxicity.

## Graphical abstract

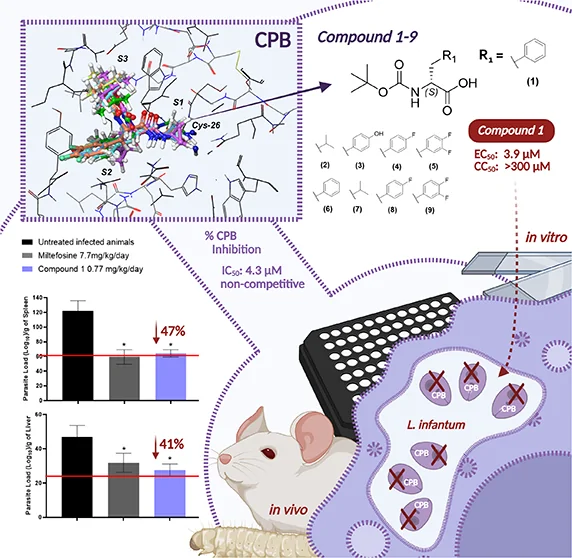

## 1 Introduction

Leishmaniasis is an infectious parasitic disease of major public health importance, particularly in tropical and subtropical regions. It is caused by parasites of the genus *Leishmania* (family *Trypanosomatidae*), which exist as intracellular amastigotes in mammalian hosts and comprise more than 20 different species capable of infecting humans, with the infecting species largely determining the clinical manifestation of the disease. The parasites are transmitted by female phlebotomine sand flies (subfamily *Phlebotominae*), primarily of the genera *Lutzomyia* in the New World and *Phlebotomus* in the Old World.^1,2,84^ The main clinical forms include visceral leishmaniasis (VL), cutaneous leishmaniasis (CL), and mucocutaneous leishmaniasis (MCL). VL, caused predominantly by *L. infantum*, is characterized by high fever, dry cough, diarrhea, fatigue, and visceromegaly; if left untreated, it can be fatal.^3–5^ CL, the most prevalent form, is typically associated with various *Leishmania* species of the subgenera *Leishmania* and *Viannia*, such as *Leishmania (Leishmania) amazonensis*, and is characterized by painless skin lesions at the site of the insect bite.^6–8^ In some cases, CL can progress to MCL, affecting the nasal cavity, oropharynx, lips, and palate, leading to destructive mucosal lesions.^9^ In Brazil, leishmaniasis is endemic in its visceral, cutaneous, and mucocutaneous forms.^10,87^ Regarding epidemiological aspects, recent data from the World Health Organization indicate that leishmaniasis remains endemic in approximately 99 countries, with an estimated 700,000–1 million new cases occurring annually. The disease represents a substantial public health burden, particularly due to VL, which is associated with approximately 20,000 to 30,000 deaths per year, while more than 1 billion people live in areas at risk of infection.^86,87^ Despite substantial progress in some endemic regions, the burden of VL remains unevenly distributed worldwide. In South Asia, the number of cases has declined markedly following successful elimination efforts, whereas Eastern Africa continues to bear the highest global burden. In South and Central America, the number of cases decreased by 57% between 2017 and 2022; however, Brazil remains the main hotspot, accounting for approximately 92% of reported VL cases in the region.^83^ Despite its considerable global health impact, leishmaniasis is classified as a Neglected Tropical Disease (NTD), reflecting a longstanding history of underinvestment in research, drug development, and the implementation of effective control strategies.^83^

The treatment of leishmaniasis is highly complex, and the choice of medication depends on factors such as the *Leishmania* species, clinical form, geographic distribution, and patient characteristics; there is no universal therapeutic regimen.^83–87^ Available options are limited to a relatively small number of drugs: pentavalent antimonials (meglumine antimoniate and sodium stibogluconate), amphotericin B (deoxycholate −AmB– and liposomal formulations −L-AmB−), pentamidine salts, paromomycin sulfate, and miltefosine.^11–13^ Among them, miltefosine is the only orally administered drug, whereas the others require hospitalization and are administered parenterally. These drugs may be administered as monotherapy or in combination, depending on the clinical manifestation. For VL, combination regimens include sodium stibogluconate plus paromomycin in Eastern Africa and L-AmB plus miltefosine in patients with HIV coinfection in South Asia and Eastern Africa. In Latin America, where *L. infantum* predominates, the recommended first-line treatment is multiple-dose L-AmB administered parenterally, whereas AmB or meglumine antimoniate are alternatives.^83^ For CL, therapeutic approaches also include local interventions, such as wound care, intralesional antimonials, thermotherapy, cryotherapy, and topical paromomycin, particularly for uncomplicated disease. Systemic treatment with miltefosine, pentamidine, L-AmB, AmB, or systemic antimonials is indicated for multiple, large, complicated, disseminated, or diffuse lesions and for immunocompromised patients.^83^ MCL, in contrast, requires systemic treatment, with antimonials used alone or in combination with pentoxifylline, while L-AmB is preferred in special situations such as pregnancy or lactation.^11–13,83^ It is worth noting that these therapies are associated with high toxicity, prolonged treatment regimens, and the growing emergence of drug-resistant parasite strains.^14,15,87^ Furthermore, immunocompromised patients, particularly those with VL and HIV coinfection, have poorer treatment responses, greater drug toxicity, and a higher risk of relapse and therefore require specialized management, including antileishmanial therapy combined with appropriate antiretroviral treatment and, in high-risk cases, secondary prophylaxis.^83^ Consequently, the identification of novel molecular targets and the development of safer, more effective, and more selective therapeutic strategies remain urgent unmet medical needs.

Peptides have emerged as promising candidates in drug development due to their high specificity toward molecular targets, low toxicity, and degradation into nontoxic metabolites.^16^ However, their clinical application is often limited by poor metabolic stability, low membrane permeability, rapid proteolytic degradation, and conformational instability.^17^ To overcome these drawbacks, peptidomimetics have been developed. These are organic molecules designed to mimic the structural and physicochemical properties of natural peptides while exhibiting improved pharmacokinetic and pharmacodynamic characteristics.^17,18^ Within this context, dipeptidyl nitriles represent a relevant class of peptidomimetic compounds, as they retain key recognition features of peptide substrates while incorporating a nitrile warhead capable of forming reversible covalent interactions with catalytic cysteine residues.^19^

Targeting essential enzymes for parasite survival is a rational strategy for the development of selective antileishmanial drugs. Among these targets, cysteine proteases (CPs) stand out as critical virulence factors involved in processes such as differentiation, nutrition, and host cell infection.^20^ In *Leishmania*, three families of cysteine protease genes have been identified (CPA, CPB, and CPC), with class B cysteine proteases (CPB) being strongly associated with parasite virulence. Genetic validation studies in *Leishmania mexicana* have provided substantial evidence supporting the role of CPB in macrophage infection, disease progression, and virulence attenuation following gene disruption.^21^ In addition to its direct contribution to infectivity, CPB has been implicated in the modulation of host–parasite interactions, promoting immune evasion and supporting intracellular survival in both promastigote and amastigote stages. Collectively, these findings reinforce CPB as a biologically relevant and strategically attractive target for antileishmanial drug development.^22^

Recent findings from our research group have demonstrated the potential of CPB inhibitors as therapeutic agents against leishmaniasis. Several chemical classes have shown promising activity, including guanidine bioconjugates,^23,24^ benzophenone-based derivatives,^25^ and *N*-oxide derivatives.^26^ In line with these observations, previous studies investigating CPB inhibitors in *Leishmania*, such as compound 14e and furoxan-derived analogues, have reported comparable reductions in parasite burden (∼50%) in experimental animal models,^26,27^ further supporting CPB as a therapeutically relevant target.

These compounds share a common structural framework consisting of a recognition sequence (peptidic or nonpeptidic) and a functional group capable of interacting with the enzyme’s active site.^28^ In line with this rationale, dipeptidyl nitriles have been explored as CPB inhibitors due to their capacity to combine substrate-like recognition with a reversible covalent mechanism of inhibition.

Dipeptidyl nitriles, or nitrile-based compounds, have been explored in different studies because of their ability to interact with cysteine proteases, including cathepsins L, S, K and papain,^29,30^ cruzain^31–35^ and *Lm*CPB.^36^ In the work of Ribeiro and collaborators (2020),^36^ a series of dipeptidyl derivatives was evaluated against *Lm*CPB, with p*K*i values ranging from 4.3 to 8.3. Despite these advances, little is known about the structure–activity relationships governing CPB inhibition in *Leishmania* spp., particularly in the absence of experimentally resolved protein structures. In this context, we employed the recombinant *Lm*CPB2.8ΔCTE enzyme, previously expressed in *Pichia pastoris* by our research group,^26^ to evaluate nitrile-based derivatives.

Considering the potent inhibitory activity of dipeptidyl nitriles against cysteine proteases and the need to establish a clearer correlation between enzyme inhibition and antiparasitic efficacy, this study aimed to evaluate the in vitro and in vivo antileishmanial activity and safety of selected derivatives against *L. amazonensis* and *L. infantum*. Our objective was to identify reversible covalent CPB inhibitors that may serve as lead compounds for future structural optimization, with the goal of enhancing leishmanicidal potency while minimizing potential host toxicity.

## 2 Results and Discussion

Given the persistent therapeutic limitations in leishmaniasis, including toxicity, high costs, emerging resistance, and limited treatment accessibility,^37–39^ there remains a critical need for new molecular scaffolds targeting validated parasite enzymes. Despite ongoing efforts in drug development, including initiatives led by Drugs for Neglected Diseases initiative (*DNDi*),^40^ the translation of promising compounds into clinically approved therapies has been limited.^40^ In this context, the present study focuses on the identification and biological characterization of nitrile-based reversible covalent inhibitors targeting *Leishmania* CPB, aiming to explore this enzyme as a pharmacologically actionable target and to establish structural scaffolds suitable for further optimization.

### 2.1 Synthesis and Molecular Docking of Dipeptidyl Nitrile Derivatives

The discovery of new bioactive molecules can be conducted through phenotypic screening approaches, in which large compound libraries are evaluated for biological activity without prior knowledge of the molecular target under study.^41–43^ Although effective for hit identification, this strategy may limit mechanistic understanding and subsequent optimization. In contrast, target-based drug design enables the rational development of molecules directed toward a validated molecular target, thereby streamlining pharmacological optimization.^44,45^ Among the validated targets in *Leishmania* spp., cysteine protease B has emerged as a particularly attractive candidate due to the reported activity of CPB inhibitors against the parasite.^46^ Based on this rationale, the dipeptidyl-nitrile derivatives evaluated in this study were designed using a target-based approach focused on CPB and selected according to the hit criteria as described previously.^47^

The compounds (**1–9**) were efficiently obtained via amide bond formation between Boc-L-amino acids and either 2-aminoacetonitrile or 1-aminocyclopropanecarbonitrile, using HATU as the coupling reagent in a basic medium with DIPEA (Scheme 1). After purification, compounds were isolated and characterized by analytical methods, including high-resolution mass spectrometry (HRMS) and nuclear magnetic resonance (NMR), confirming the expected chemical structures with yields ranging from 12 to 67%.

**Scheme 1:**
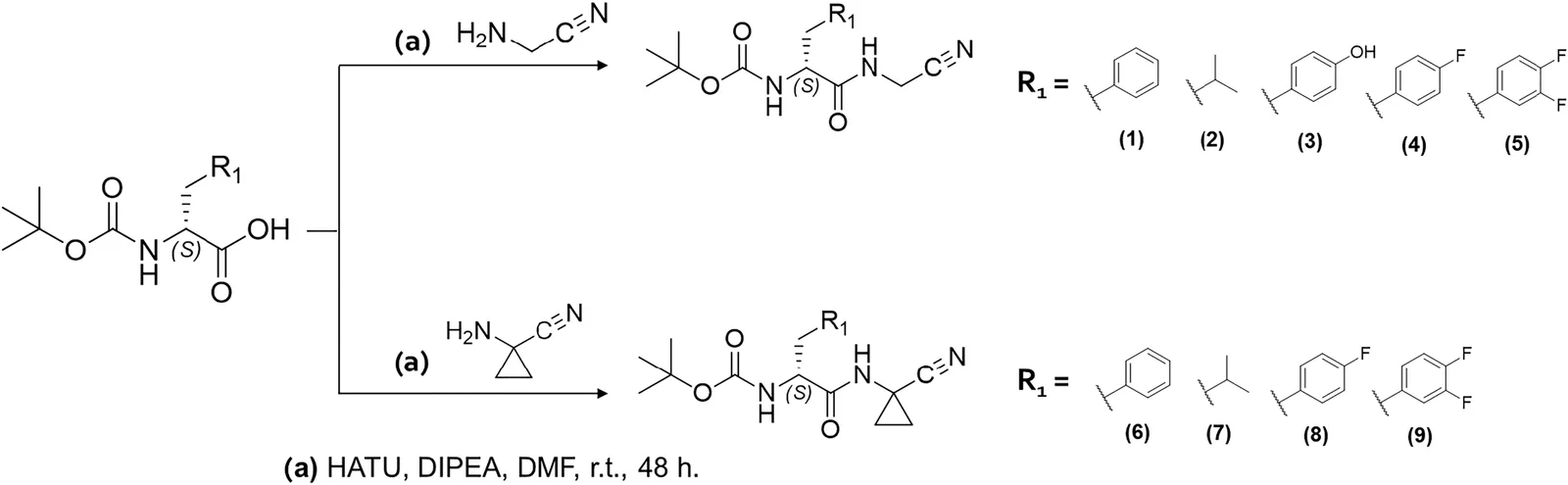
Synthetic Methods Employed for the Synthesis of Compounds **1**–**9**

Molecular docking was performed to identify the interactions between the molecules and the active site of CPB. Considering the two warheads present in the series of dipeptidyl nitrile, docking was performed in a covalent mode. Docking score and MMGBSA values were fully presented in Table S1 (Supporting Information). Binding energies were similarly obtained, ranging from −6.173 to −6.709 for compounds **1**, **3**, **4–6**, and **9**. However, compounds **2** and **7** showed the lowest values: −3.819 and −4.541, respectively, both containing the isopropyl subunit. This suggests that this group may not be the best fit for interaction with the CPB active site. Compounds K777 and E64, both known cysteine-protease inhibitors,^48,49^ were used as controls in our docking studies (Table S1, Supporting Information) Superposition of compound **1** and E64 showed that the molecules interact with different regions in the CPB catalytic site, due to differences in their size and structural flexibility (Figure S1, Supporting Information).

Compounds exhibited similar binding poses, interacting with amino acid residues located in the S2 and S3 regions of the protease (Figure 1). In the lowest-energy pose, the Boc-carbamate group preferentially occupies the S3 region, forming a hydrogen bond with a glycine residue (GLY67). The phenyl ring or isopropyl group occupies the S2 region, which is predominantly hydrophobic. These results indicate that the dipeptidyl nitrile derivatives show favorable accommodation within the CPB active site, suggesting strong interactions with the protease.

**Figure 1:**
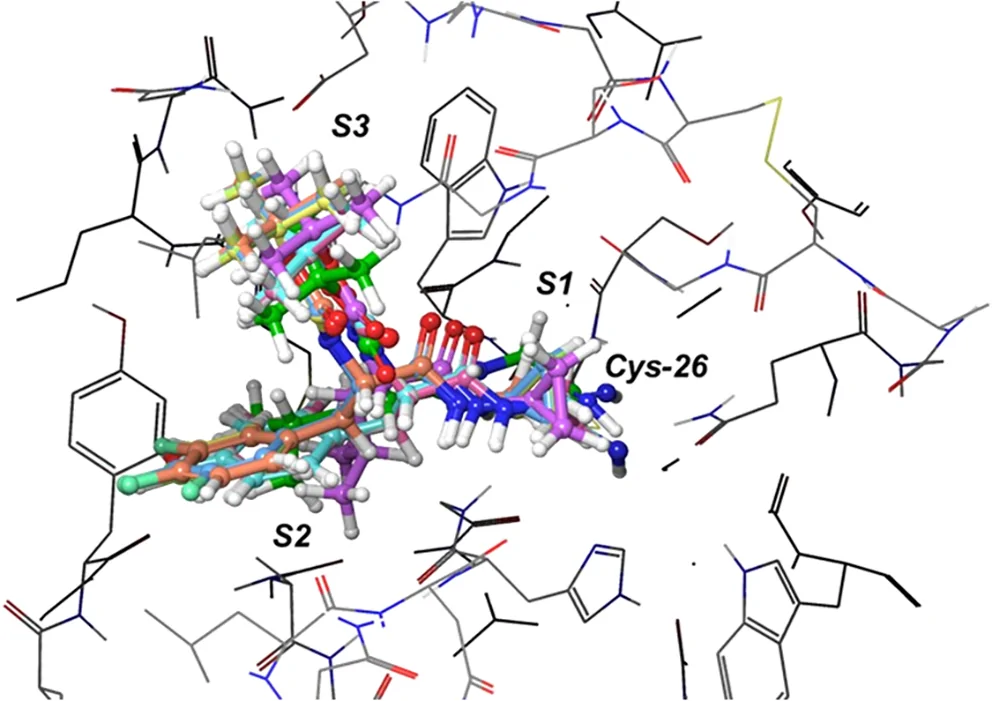
Covalent docking poses of compounds **1–9** within the CPB active site (3D representation).

### 2.2 Identification of Reversible Covalent CPB Inhibitors and *In Vitro* Antileishmanial Activity

The synthesized nitrile-based derivatives were initially evaluated for their inhibitory activity against CPB, a validated cysteine protease that plays a key role in parasite virulence and intracellular survival. To define their potential as reversible covalent inhibitor scaffolds, we assessed host-cell cytotoxicity, enzymatic inhibition of recombinant *Lm*CPB, and antileishmanial activity against intracellular amastigotes, the clinically relevant form of the parasite.

The cytotoxicity of the series of dipeptidyl nitrile derivatives was evaluated against murine peritoneal macrophages (CC_50_), as shown in Table 1. None of the compounds exhibited detectable cytotoxicity at or below the highest concentration tested (300 μM), indicating that their CC_50_ values were above 300 μM under the experimental conditions used. In contrast, the reference drug miltefosine exhibited a CC_50_ value of 88.2 ± 5.3 μM.

**Table 1:** Mammalian Cell Cytotoxicity (CC_50_), CPB Inhibition (IC_50_), and Anti-Amastigote Activity (EC_50_) against *Leishmania* spp., Expressed in μM^c^,^d^

				**EC**_**50**_ **(SI)**	
**Compound**	**CPB inhibition (%)**	**IC**_**50**_	**CC**_**50**_	***La***	***Li***
**1**	83.9 ± 2.2	4.3 ± 0.2	>300	4.6 ± 1.6 (∼**65**)	3.9 ± 0.5 (∼**77**)
**2**	90.9 ± 2.3	1.9 ± 0.8	>300	14.6 ± 0.3 (∼**21**)	>30 (ND)
**3**	78.4 ± 5.1	23.5 ± 1.0	>300	21.6 ± 1.0 (∼**14**)	>30 (ND)
**4**	86.2 ± 3.2	5.9 ± 0.4	>300	>30 (ND)	14.7 ± 3.2 (∼**20**)
**5**	70.6 ± 4.1	3.9 ± 0.3	>300	>30 (ND)	>30 (ND)
**6**	93.2 ± 0.9	0.8 ± 0.1	>300	23.0 ± 1.7 (∼**13**)	>30 (ND)
**7**	77.5 ± 3.5	16.4 ± 1.0	>300	12.3 ± 1.7 (∼**24**)	>30 (ND)
**8**	73.3 ± 4.8	12.5 ± 3.0	>300	>30 (ND)	18.8 ± 4.0 (∼**16**)
**9**	56.5 ± 2.5	>20	>300	>30 (ND)	>30 (ND)
**Miltefosine** ^a^	NE	NE	88.2 ± 5.3	5.4 ± 0.3 (∼**16**)	7.8 ± 0.1 (∼**11**)
**E64** ^b^	99.4 ± 5.0	<0.3	NE	NE	NE

a Miltefosine was used as a control for *L. amazonensis* (*L.a*) and *L. infantum* (*L.i*).

b E64 was used as a control inhibitor (standard) for CPB.

c Percentage of CPB inhibition at the highest concentration tested (20 μM).

d EC_50_ is Defined as the Effective Concentration Required to Reduce Intracellular Amastigote Viability by 50%. Data are presented as the mean ± standard deviation (SD) of three independent experiments. *La:* *L. amazonensis* *; Li:* *L. infantum*. The selectivity index (SI) was calculated as SI = CC_50_/EC_50_ and is shown in bold. NE: not evaluated; ND: not determined.

Compounds **1**, **2**, and **7** were described previously,^36^ exhibiting p*K_i_* values of 5.8, 5.8, and 6.3, respectively, against *Lm*CPB. In the present study, the inhibitory activity of the dipeptidyl nitrile derivatives was further evaluated against recombinant cysteine protease *Lm*CPB2.8ΔCTE^26^ using a dose–response enzymatic assay. All compounds demonstrated measurable inhibition of *Leishmania* CPB at 20 μM, although the magnitude of inhibition varied across the series.

Compounds **1** and **6** exhibited the highest inhibition percentages (approximately 84% and 93%, respectively), indicating stronger functional interference with enzyme activity. Notably, the previously reported p*K*_i_ values for compounds **1** and **2** (5.8) are consistent with their observed inhibitory profiles, supporting their moderate affinity for CPB. Although compound **7** displayed a slightly higher p*K_i_* (6.3), its inhibition under the present assay conditions was less pronounced, suggesting that equilibrium binding affinity does not necessarily translate into maximal inhibition at a fixed concentration.

The enzymatic inhibition data obtained at 20 μM (Table 1) revealed a clear structure–activity relationship (SAR) among the evaluated compounds. Most derivatives exhibited inhibition levels above 70%, whereas compounds **5** and **9** were among the least active, with 70.6% and 56.5% inhibition, respectively. In contrast, compounds **2** and **6** showed the highest inhibitory activity, with inhibitory percentages exceeding 90%. Despite this apparent SAR in the enzymatic assay, this trend was not consistently reflected in the phenotypic assays using intracellular amastigotes. Compounds **2** and **6**, although highly active against CPB, displayed only moderate antiamastigote activity, with EC_50_ values above 10 μM. Conversely, compounds **5** and **9**, which share a 3,4-difluorophenyl moiety at the R1 position, showed no detectable activity against either *Leishmania* species at the highest screening concentration tested (30 μM), which was selected as a rational screening threshold based on the established criteria for antileishmanial drug prioritization^47^.

Antiamastigote activity varied across the series, indicating that structural changes affected responses against the two *Leishmania* species. Compound **1** was the only derivative showing similar activity against *L. amazonensis* and *L. infantum*, with EC_50_ values of 4.6 ± 1.6 and 3.9 ± 0.5 μM, respectively. This compound combines an unsubstituted benzyl side chain with a cyanomethyl nitrile warhead and was the most active analog evaluated against both species.

The intracellular activity of compound **1** was further supported by its effect on the infection index of *Leishmania*-infected macrophages (Figure S2, Supporting Information). Treatment with compound **1** resulted in a concentration-dependent reduction in the infection index for both *L. amazonensis* and *L. infantum* compared with the respective untreated controls. This effect was accompanied by a progressive reduction in the number of intracellular amastigotes observed microscopically. These findings provide complementary phenotypic evidence that compound **1** reduces parasite burden within macrophages, consistent with the antiamastigote activity determined in the EC_50_ assays. The comparable response observed for both species further supports the broad intracellular activity of compound **1** within the present series (Figure S2).

Modification of the benzyl moiety markedly affected antiamastigote activity. Introduction of a para-hydroxy substituent, as in compound **3**, reduced activity against *L. amazonensis* approximately 5-fold relative to compound **1** (EC_50_ = 21.6 ± 1.0 μM) and resulted in no measurable activity against *L. infantum* at concentrations up to 30 μM. Although the present data set does not allow the physicochemical basis of this effect to be established, this result suggests that increased polarity or altered interactions associated with para-hydroxylation are not favorable for intracellular activity in this scaffold. Para-fluorination generated a different profile: compounds **4** and **8** retained moderate activity against *L. infantum* (EC_50_ = 14.7 ± 3.2 and 18.8 ± 4.0 μM, respectively), while no activity was detected against *L. amazonensis* at the highest concentration tested. Further fluorination was detrimental, since the 3,4-difluorophenyl derivatives **5** and **9** were inactive against both species, regardless of the nitrile warhead.

Replacing the aromatic side chain with the leucine-derived isobutyl group also altered the phenotypic profile. Compounds **2** and **7** showed moderate activity against *L. amazonensis* (EC_50_ = 14.6 ± 0.3 and 12.3 ± 1.7 μM, respectively) but no measurable activity against *L. infantum*. Interestingly, these compounds also showed the least favorable docking scores in the series, suggesting less favorable accommodation of the aliphatic side chain within the predominantly hydrophobic S2 region of CPB. Nevertheless, both compounds retained substantial enzymatic inhibition, emphasizing that docking score, enzyme inhibition, and intracellular efficacy should not be interpreted as directly interchangeable parameters.

Comparison of matched structural pairs further suggests that the effect of the nitrile warhead is context-dependent. The clearest example is provided by compounds **1** and **6**, which share the same unsubstituted benzyl side chain but differ in the nitrile warhead. Introduction of the 1-cyanocyclopropyl group markedly increased CPB inhibitory potency (IC_50_ = 0.8 μM for compound **6** vs 4.3 μM for compound **1**) but simultaneously reduced antiamastigote activity against *L. amazonensis* (EC_50_ = 23.0 vs 4.6 μM) and abolished measurable activity against *L. infantum*. Conversely, analogous warhead replacement had only modest effects in the leucine-derived pair **2/7** and in the para-fluorophenyl pair **4/8**, while both 3,4-difluorophenyl derivatives **5** and **9** remained inactive.

These data indicate that CPB inhibitory potency alone is insufficient to predict intracellular antiamastigote efficacy. The phenotypic activity appears to depend on the combined contribution of the side chain and nitrile warhead, in addition to factors not captured by the isolated enzyme assay, such as cellular permeability, intracellular stability, compound distribution within the macrophage–parasite system, and access to the parasitic target. Within the present series, the unsubstituted benzyl/cyanomethyl combination of compound **1** provided the most favorable balance between CPB inhibition (∼84%) and intracellular activity against both *Leishmania* species.

In addition, compound **1** demonstrated a high degree of selectivity toward both *Leishmania* species, with selectivity indices of approximately 65 for *L. amazonensis* and 77 for *L. infantum*. Against *L. infantum*, compound **1** was approximately 2-fold more potent than the reference drug miltefosine, as indicated by its lower EC_50_ value (3.9 ± 0.5 μM vs 7.8 ± 0.1 μM) and substantially higher selectivity index (∼77 vs ∼11). Together, these findings indicate that compound **1** combines greater antiamastigote potency with a more favorable selectivity profile than miltefosine, supporting its prioritization for subsequent in vivo evaluation.

In this context, cysteine proteases (CPs), including CPA, CPB, and CPC, have been widely described in the literature as key virulence factors in *Leishmania* spp., playing essential roles in parasite differentiation, survival, and host–pathogen interactions. Their expression is enriched in amastigotes, reinforcing their relevance in the clinically important intracellular stage. In particular, CPB has been reported as a multifunctional enzyme involved in immune evasion, including modulation of host signaling pathways and interference with antigen presentation. Additionally, its COOH-terminal extension (cyspep), released during pro-CPB maturation inside macrophages, has been shown to modulate host immune responses by altering cytokine profiles associated with Th1/Th2 balance, thereby favoring parasite persistence. Given the conserved role of CPs across *Leishmania* species, the observed activity of compound **1** against both *L. amazonensis* and *L. infantum* may be associated with interference in CP-mediated pathways. Supporting the relevance of CPs in species-specific biology, studies have shown that, in *L. donovani* and *L. major*, single nucleotide polymorphisms (SNPs) in genes encoding these enzymes can influence whether the infection presents a dermatotropic or viscerotropic profile. Consistently, *L. infantum* has been reported to express higher levels of CPB than species associated with cutaneous disease, such as *L. major* and *L. tropica*.^50,51^

To gain mechanistic insights into the biological activity of compound **1**, enzyme kinetics studies were performed to evaluate its effect on *Leishmania* cysteine protease B (CPB), a key enzyme involved in parasite survival and virulence. CPB exhibited classical Michaelis–Menten behavior, with a *K*_m_ of 14.1 μM in the absence of inhibitors, indicating that the purified enzyme retained its structural integrity and catalytic affinity for the fluorogenic substrate (Z-Phe-Arg-AMC).^52,53^ Kinetic analysis of compound **1**, as shown in Figure 2, revealed a mixed inhibition profile, while the corresponding Michaelis–Menten curves are presented in Figure S3.

**Figure 2:**
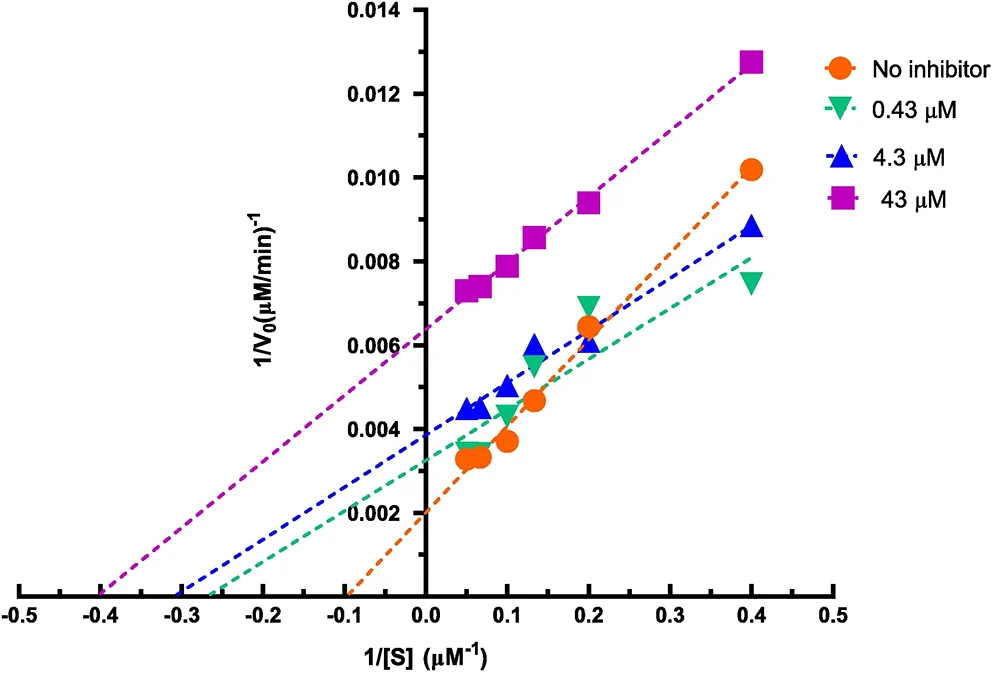
Double reciprocal Lineweaver–Burk plot showing the relationship between the reciprocal of the cysteine protease B enzyme activity (1/V_0_) and the reciprocal of the substrate concentration (1/[S]). The intercept of the plot with the abscissa gives the reciprocal of the Michaelis–Menten constant (–1/*K*_m_) in the absence (*w*/*o*) and in the presence of 0.43 μM, 4.3 μM; and 43 μM of compound **1**.

The kinetic parameters further support the mixed inhibition model, as compound **1** caused a reduction of approximately 76.8% in Vmax (from 669.4 to 155.5 RFU·min^–1^) and an 83.1% decrease in *K*_m_ (from 14.1 to 2.3 μM).^54^ A similar kinetic profile was observed for compounds **2** and **6** (Figures S4–S7). These data are consistent with a mixed inhibition mechanism, in which the inhibitor can interact with both the free enzyme and the enzyme–substrate complex. This behavior differs from classical competitive inhibition, in which *K*_m_ increases while Vmax remains unchanged. Importantly, the kinetic data provide mechanistic support for CPB inhibition by compound **1**; together with its strong intracellular antiamastigote activity and the concentration-dependent reduction in infection index, these findings are consistent with CPB being a potentially relevant molecular target of this compound.

### 2.3 ADME Profiling Identified Compound 1 As One of the Most Balanced Compounds in the Series

Compound **1** displayed a moderate lipophilicity, with an eLogD value of 2.49 at pH 7.4, together with kinetic solubility values of 61.05 μM at pH 2.0 and 51.61 μM at pH 7.4. This profile suggests that compound **1** maintains adequate aqueous solubility under both acidic and near-physiological conditions. In the PAMPA assay, compound **1** showed high passive permeability, with a Pe value of 18.66 × 10^–6^ cm/s and good recovery (91.55%), indicating that the permeability measurement was not substantially affected by nonspecific loss or membrane retention.

Metabolic stability studies further supported the favorable developability profile of compound **1**. In human liver microsomes, compound **1** exhibited a long half-life of 141.46 min, low intrinsic clearance (CLint = 19.6 μL/min/mg), and 75.38% of the parent compound remaining after 60 min. In the absence of NADPH, 98.19% of compound **1** remained after incubation, indicating minimal nonenzymatic degradation. In mouse liver microsomes, however, compound **1** showed lower metabolic stability, with a half-life of 58.74 min, moderate intrinsic clearance (CLint = 47.2 μL/min/mg), and 54.64% remaining after 60 min. These data indicate a species-dependent difference in microsomal metabolism, with faster turnover in mouse than in human microsomes.

The integrated ADME profile of compound **1** is summarized in Table 2. Overall, compound **1** combined balanced lipophilicity, acceptable kinetic solubility, high passive permeability, and favorable human microsomal stability, although moderate clearance in mouse liver microsomes suggests that in vivo exposure should be confirmed before efficacy studies are interpreted.

**Table 2:** *In Vitro* ADME Profile of Compound **1** ^a^

Parameter	Compound 1 value	Interpretation
eLogD pH 7.4	2.49	Balanced lipophilicity
Kinetic solubility pH 2.0/7.4	61.05/51.61 μM	Acceptable solubility at acidic and physiological pH
PAMPA Pe	18.66 × 10^–6^ cm/s	High passive permeability
PAMPA recovery	91.55%	Reliable assay recovery
HLM t_1_/_2_/CLint	141.46 min/19.6 μL/min/mg	Low human microsomal clearance
HLM remaining ± NADPH	75.38%/98.19%	Metabolism-dependent depletion; minimal nonenzymatic degradation
MLM t_1_/_2_/CLint	58.74 min/47.2 μL/min/mg	Moderate mouse microsomal clearance
MLM remaining ± NADPH	54.64%/99.49%	Faster mouse metabolism; minimal nonenzymatic degradation

a Kinetic solubility was determined at pH 2.0 and 7.4. Passive permeability was assessed using PAMPA. Metabolic stability was evaluated in human liver microsomes (HLM) and mouse liver microsomes (MLM) in the presence or absence of NADPH. CLint: intrinsic clearance; Pe: effective permeability; t_1_/_2_: half-life.

Although potency remains the primary driver for compound prioritization, the ADME data provide an important developability filter for this series. In this context, compound **1** emerged as a particularly balanced compound. Its moderate eLogD, maintained solubility across acidic and physiological pH, and high PAMPA permeability indicate a favorable interplay between lipophilicity, solubility, and passive diffusion. Importantly, compound **1** also showed low intrinsic clearance in human liver microsomes, suggesting that rapid oxidative metabolism is unlikely to be a major liability in the human microsomal system.

However, the lower stability observed in mouse liver microsomes highlights a potential translational caveat. The faster turnover in MLM may reduce systemic exposure in murine efficacy models and should therefore be considered before interpreting negative or suboptimal in vivo results as a lack of intrinsic pharmacological activity. This species-dependent metabolic behavior reinforces the need for in vivo PK evaluation, particularly if compound **1** is advanced to proof-of-concept studies in mice. Thus, compound **1** should be regarded not simply as the compound with favorable individual parameters, but as a candidate with a coherent and relatively low-risk early ADME profile, pending confirmation of exposure *in vivo*.

### 2.4 Determination of Toxicity in an Alternative Model of *Galleria mellonella*

Among the important steps in drug development is the pharmacokinetic characterization of candidate molecules, including the evaluation of absorption, distribution, metabolism, excretion, and physicochemical properties. Criteria such as the Rule of Five (Ro5) are widely used to identify compounds with favorable oral bioavailability profiles and acceptable safety parameters.^55,56^ In this regard, the ADME analysis performed in the present study indicated that compound **1** complies with these parameters, supporting its potential suitability for oral administration.

To further investigate *in vivo* toxicity using an alternative model, *G. mellonella* larvae were employed, representing an important strategy to reduce vertebrate animal use while enabling preliminary toxicity assessment. This model has gained increasing relevance due to its low cost, ease of handling, and innate immune responses that share functional similarities with those of mammals.^58,59^ Additionally, previous studies have demonstrated a strong correlation between toxicity findings in *G. mellonella* and rodent models when evaluating the same compound.^60^

Larval health status was assessed using the Health Index Scoring System (scores from 1 to 10), based on four parameters: mobility, cocoon formation, melanization, and survival. DMSO was used as a solubilizing agent, and its use at concentrations up to 6% was confirmed to be nontoxic under the experimental conditions.

Following administration of compound **1** at 2.8 mM (corresponding to 95 mg/kg/day), no signs of toxicity were observed. The larvae maintained normal mobility, cocoon formation, and melanization (Figure 3), with no mortality recorded (Figure S8). Together with the absence of hemolytic activity, these findings reinforce the favorable preliminary safety profile of compound **1** and support its progression to subsequent *in vivo* studies in mammalian models.

**Figure 3:**
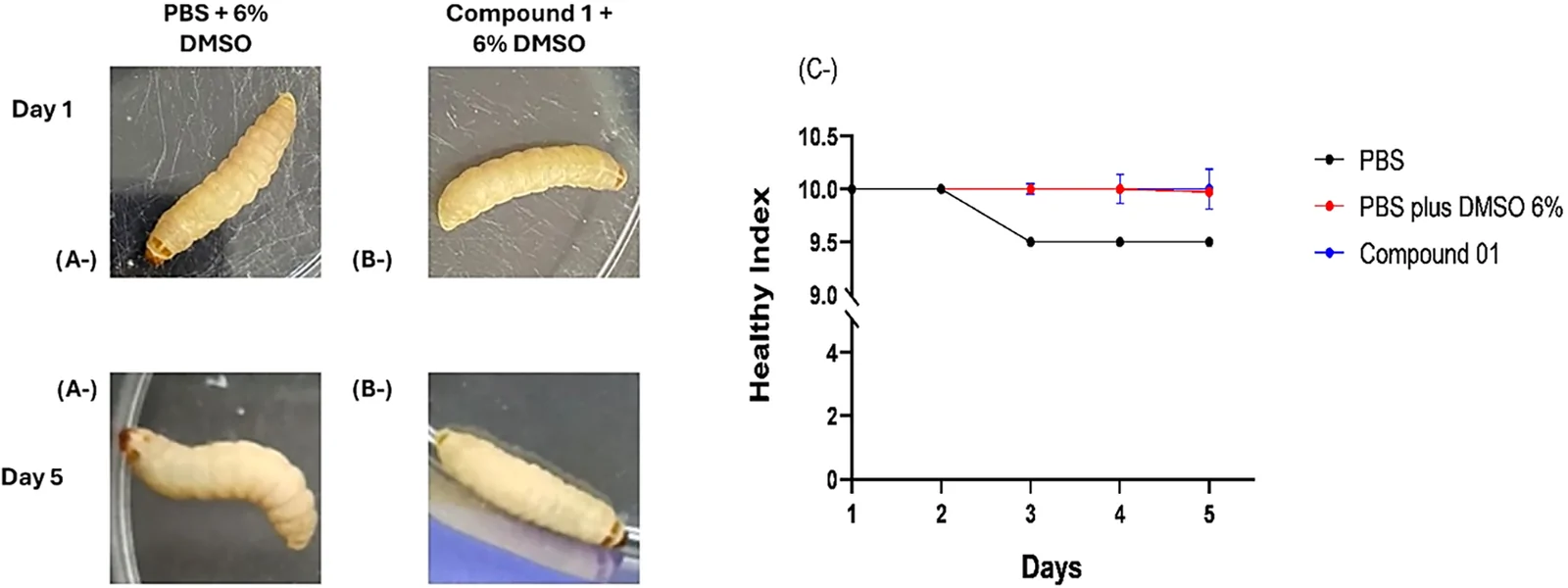
Determination of toxicity in an alternative model of *G. mellonella*. Follow up for 5 days after inoculation and observation of larval motility, cocoon formation, melanization, and survival. (A) Administration of PBS plus 6% DMSO vehicle; (B) compound **1** solubilized in 6% DMSO. (C) Health status of *G. mellonella* larvae following treatment with compound **1** over 5 days. Experiments were performed in triplicate, with *n* = 10 larvae per group. Data is expressed as mean ± SD. No statistically significant differences were observed among groups (*p* > 0.05).

### 2.5 Evaluation of *In Vivo* Efficacy and Safety in BALB/c Mice Infected with *L. infantum*

Based on its *in vitro* efficacy, CPB inhibitory activity, low cytotoxicity, favorable ADME profile, and absence of toxicity in *G. mellonella*, compound **1** was prioritized for oral evaluation in a murine model of visceral leishmaniasis.

BALB/c mice infected with *L. infantum* were orally treated for 5 days with compound **1** at a dose of 0.77 mg/kg/day, while miltefosine (Cayman Chemical; 7.7 mg/kg/day) was used as the reference drug in the control group.^26^ Parasite load was determined by limiting dilution and LDU index, followed by biochemical evaluation of hepatic and renal markers.^72,80,81,88^

Treatment with compound **1** resulted in reductions in parasite burden in the spleen of approximately 55% and 47%, as assessed by the LDU index and limiting dilution methods, respectively. In the liver, parasite burden was reduced by 44% and 41% according to the same methods, respectively (Figures 4 and 5). By comparison, miltefosine treatment resulted in reductions of 52% and 51% in the spleen and 65% and 32% in the liver, as determined by the LDU index and limiting dilution methods, respectively. Notably, compound **1** achieved these effects at a dose 10-fold lower than that of miltefosine, highlighting its promising *in vivo* antiparasitic activity.

**Figure 4:**
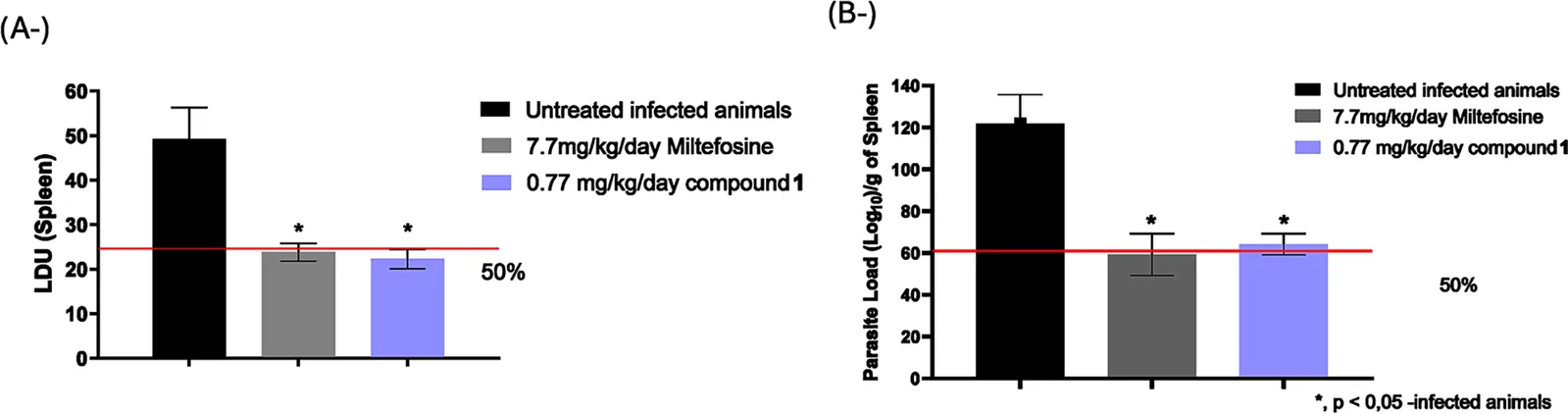
Quantification of parasite load in the spleen of BALB/c mice infected with *L. infantum*. (A) Parasite load determined using the Leishman–Donovan Units (LDU) method; and (B) parasite load determined using the limiting dilution method. Data are presented as the mean ± SD of infected animals treated orally by gavage for 5 consecutive days in the following groups: untreated infected animals; miltefosine (7.7 mg/kg/day); and compound **1** (0.77 mg/kg/day). The horizontal red line indicates 50% of the parasite load measured in the infected, untreated control group and serves as a reference for determining the level of parasite reduction achieved by each treatment. *Statistically significant differences compared with the infected, untreated control group (*p* < 0.05); the asterisk indicates that both miltefosine- and compound **1**-treated groups differed significantly from the infected, untreated group.

**Figure 5:**
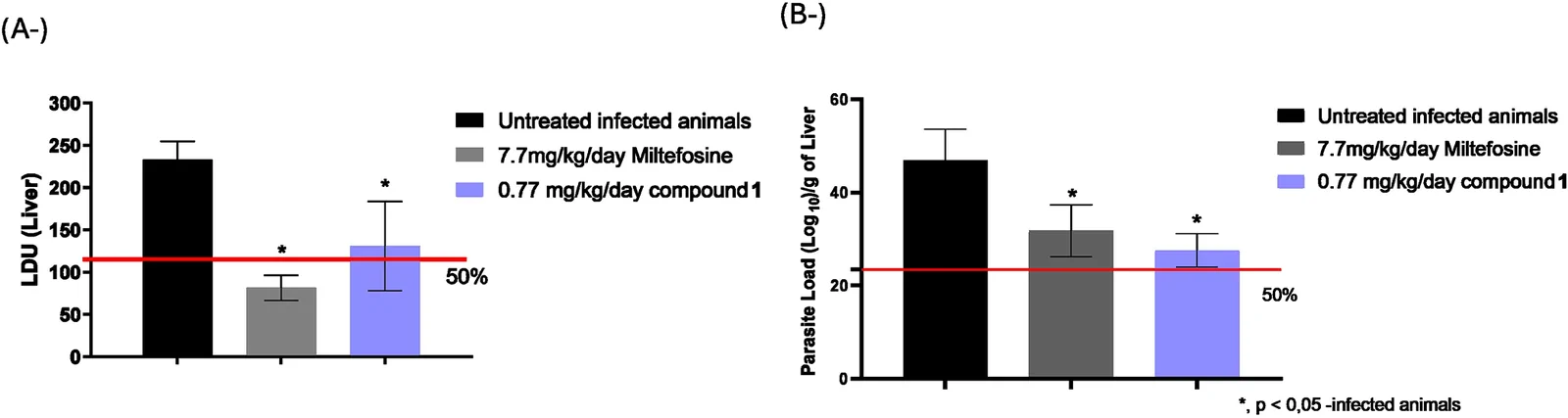
Quantification of parasite load in the liver of BALB/c mice infected with *L. infantum*. (A) Parasite load determined using the Leishman–Donovan Units (LDU) method; and (B) parasite load determined using the limiting dilution method. Data are presented as the mean ± SD of infected animals treated orally by gavage for 5 consecutive days in the following groups: untreated infected animals; miltefosine (7.7 mg/kg/day); and compound **1** (0.77 mg/kg/day). The horizontal red line indicates 50% of the parasite load measured in the infected, untreated control group and serves as a reference for determining the level of parasite reduction achieved by each treatment. *Statistically significant difference compared with the infected, untreated control group (*p* < 0.05); the asterisk indicates significant differences for both miltefosine- and compound **1**-treated groups.

Following treatment with compound **1**, in addition to parasite burden quantification, hepatic and renal biochemical markers were evaluated to assess potential systemic toxicity. Infected animals exhibited increased total bilirubin levels compared to healthy controls, consistent with disease-associated hepatic impairment. Notably, treatment with either miltefosine or compound **1** did not reverse this alteration. In parallel, alkaline phosphatase (ALP) activity was significantly reduced in infected animals, including treated groups, suggesting an infection-related hepatic dysfunction rather than a drug-induced effect (Figure 6A,B).

**Figure 6:**
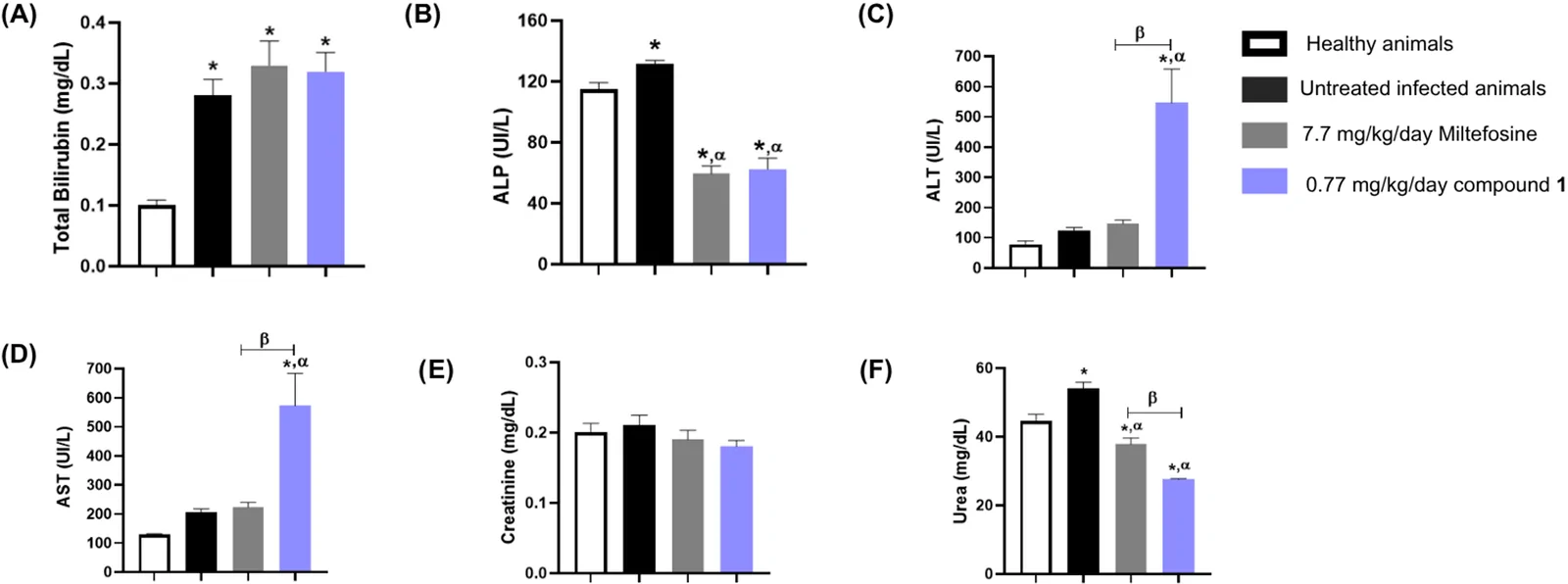
Plasma levels of hepatic and renal biomarkers in healthy and *L. infantum*-infected BALB/c mice after 5 days of treatment with compound **1** or miltefosine. (A) Total bilirubin; (B) ALP; (C) ALT; (D) AST; (E) Creatinine; (F) Urea. Data are expressed as mean ± SD *Statistically significant difference compared with healthy animals; α, statistically significant difference compared with infected untreated animals; β, statistically significant difference between treated groups (*p* < 0.05).

Regarding hepatocellular injury markers, ALT and AST levels were markedly elevated in the compound **1**-treated group compared to both healthy and untreated infected animals. These increases are indicative of hepatocellular stress or damage. However, since this effect was not observed in the untreated infected group, the contribution of compound **1** to these alterations cannot be excluded and warrants further investigation (Figure 6C,D).

Concerning renal function, creatinine levels remained stable across all experimental groups, indicating preserved glomerular function. In contrast, urea levels were elevated in infected untreated animals and significantly reduced in both treated groups, particularly in animals receiving compound **1.** This reduction may reflect a partial normalization of metabolic alterations associated with infection rather than direct renal impairment (Figure 6E,F).

Despite the promising antiparasitic efficacy observed *in vivo*, the alterations detected in hepatic biomarkers, particularly ALT and AST, indicate that further investigation is required to elucidate the underlying mechanisms and to optimize the safety profile of the compound.^61,62^ Importantly, these findings provide valuable guidance for future structural optimization, as compound **1** represents an intermediate scaffold in the development of more refined analogues with improved safety and reduced toxicity.

Hemolysis of some samples can significantly interfere with the interpretation of biochemical markers, including bilirubin, ALT, AST, and urea levels, potentially leading to misinterpretation of hepatic and renal function tests.^63–65^ Therefore, the hemolytic pattern observed in some blood samples collected by cardiac puncture during the *in vivo* study raised the possibility that the biochemical alterations observed could have been influenced by hemolysis rather than by the treatment itself. To investigate whether compound **1** could be responsible for this observation, its hemolytic activity was evaluated in vitro as a complementary assessment of its safety profile and potential to disrupt erythrocyte membrane integrity.

Compound **1** showed no detectable hemolytic activity even at the highest concentration tested (3.69 mM), corresponding to ten times the estimated plasma-equivalent concentration associated with the *in vivo* dose of 0.77 mg/kg/day (0.369 mM) (Figure S9). Serial dilutions of compound **1** likewise showed no detectable hemolytic activity. No significant hemolysis was observed for compound **1** compared with the Triton control, while neither PBS nor PBS plus DMSO induced detectable hemolytic activity. These findings indicate that compound **1** has a low potential to induce erythrocyte membrane disruption and suggest that the hemolytic pattern observed in some in vivo blood samples was unlikely to be directly associated with the treatment. Consequently, hemolysis is unlikely to have been a major confounding factor underlying the observed alterations in biochemical markers, although further mechanistic studies are warranted to clarify the origin of the hepatic and renal parameter changes. Hemolysis assays therefore provide a complementary evaluation of membrane-related toxicity and are particularly relevant for compounds intended for systemic administration.^57^

Collectively, the results of this study demonstrate a coherent translational progression from rational molecular design to *in vivo* validation. Molecular docking indicated productive accommodation of compound **1** within the S2 and S3 subsites of the CPB catalytic pocket, consistent with favorable interactions predicted for the dipeptidyl nitrile scaffold. This structural positioning was functionally supported by enzymatic inhibition data, which demonstrated that compound **1** acts as a mixed inhibitor of CPB. Furthermore, the correlation between IC_50_ and EC_50_ values reinforced CPB as a relevant intracellular target. The observed relationship between target engagement and antiparasitic activity against *L. infantum* and *L. amazonensis* strengthens the mechanistic consistency of the findings. Additionally, ADME analysis indicated compliance with drug-likeness parameters, supporting its potential suitability for oral administration.

The antiparasitic efficacy observed in the murine model supports CPB as a pharmacologically relevant intracellular target and demonstrates that the nitrile-based scaffold retains activity in a complex mammalian host environment, even at a dose 10-fold lower than miltefosine. Importantly, parasite burden was assessed using two complementary methodologies: limiting dilution and the Leishman-Donovan Unit (LDU) index. Limiting dilution quantifies viable parasites through serial dilution and culture-based outgrowth, providing a functional measure of parasite viability and replicative capacity.^72,80,81,88^ In contrast, the LDU index estimates parasite density microscopically relative to host cell nuclei, offering a direct morphometric assessment of tissue parasitism. While limiting dilution is highly sensitive for detecting viable organisms, LDU provides spatial and histopathological context within infected organs. The combined use of both methods strengthens the robustness of the efficacy data by integrating viability-based and morphometric quantification approaches, reducing methodological bias and increasing translational reliability. This dual approach is supported by previous studies, including,^66–68^ which describe the application and complementarity of these techniques in experimental visceral leishmaniasis models.

Although biochemical alterations suggestive of hepatic stress were observed, renal parameters remained largely preserved, and hemolytic activity was excluded as a confounding factor. Furthermore, the absence of toxicity in the alternative *G. mellonella* model, together with selective *in vitro* activity, indicates that compound **1** maintains a favorable therapeutic window under the tested conditions. In this context, the biochemical findings provide valuable insight in guide rational structural refinement and safety optimization.

Notably, this study represents the first *in vivo* evaluation of nitrile-based dipeptidyl CPB inhibitors within this chemical class.^31,35,36,69,70^ While this novelty strengthens the translational relevance of the findings, it also underscores the need for continued structural optimization and expanded safety assessment. Overall, compound **1** emerges as a promising intermediate scaffold that integrates target validation, cellular efficacy, *in vivo* activity, and preliminary safety profiling, providing a rational foundation for the development of more refined analogues with improved solubility, reduced potential hepatic burden, and enhanced pharmacological balance.

## 3 Conclusions

This study describes the design and biological evaluation of a series of nitrile-based dipeptidyl compounds targeting *Leishmania* cysteine protease B (CPB). The compounds demonstrated CPB inhibitory activity and, importantly, this study provides the first evidence of their phenotypic antileishmanial activity against intracellular *Leishmania* amastigotes and their *in vivo* evaluation. Among the series, compound **1** showed the most favorable overall biological profile, exhibiting potent inhibition of recombinant *Lm*CPB2.8ΔCTE, mixed inhibition kinetics, activity against intracellular *L. infantum* and *L. amazonensis* amastigotes, and a favorable selectivity profile. Its stability under serum and acidic conditions, together with the absence of detectable toxicity in *G. mellonella* and hemolytic activity, further supported its selection for exploratory *in vivo* evaluation.

Oral administration of compound **1** to *L. infantum*-infected BALB/c mice resulted in a significant reduction in parasite burden in both the liver and spleen, demonstrating measurable *in vivo* antileishmanial activity at a dose 10-fold lower than that used for the reference drug miltefosine. Although the magnitude of parasite reduction was moderate and alterations in some hepatic biochemical markers were observed, no evidence of renal dysfunction was detected. Importantly, the absence of detectable hemolytic activity at a concentration substantially higher than that used *in vivo* suggests that the hemolytic pattern observed in some blood samples was unlikely to be directly related to compound **1**.

Overall, the present findings establish nitrile-based dipeptidyl compounds as a promising chemical framework for targeting *Leishmania* CPB and provide initial evidence that this pharmacophore can translate into phenotypic and *in vivo* antileishmanial activity. Compound **1** represents a valuable starting scaffold for further medicinal chemistry optimization aimed at improving potency, efficacy, and safety. Future studies addressing structural optimization, pharmacokinetic behavior, and the mechanisms underlying its *in vivo* activity will be important to further define the therapeutic potential of this series.

## 4 Experimental Section

### 4.1 General

All the reagents were of analytical purity and purchased from Acros, Alfa Aesar, Oakwood Chemical, Sigma-Aldrich, and Synth, with no need for additional purification. Sensitive reactions were carried out under an inert atmosphere (N_2_). TLC was carried out on aluminum sheets (plates) with silica gel 90 Å Merck (visualization with fluorescence indicator under UV at λ = 254 nm). Column chromatography was conducted on Sigma-Aldrich 60 Å silica gel (40–60 μm, 230–400 mesh) (Sigma-Aldrich, St Louis, MO) using ethyl acetate, *n*-hexane, and methanol as eluents (mobile phase).

^1^H and ^13^C NMR spectra were recorded on Bruker Fourier 300 (300 MHz for ^1^H and 75 MHz for ^13^C) and Bruker Avance III 600 (600 MHz for ^1^H and 150 MHz for ^13^C) spectrometers. Two-dimensional experiments (HSQC and HMBC) were performed on the Avance III 600 system. Chemical shifts (δ) are reported in parts per million (ppm) relative to the solvent residual peak (DMSO-*d*_6_: δH = 2.50 ppm, δC = 39.52 ppm). Coupling constants (*J*) are given in hertz (Hz). Multiplicities are denoted as s (singlet), d (doublet), t (triplet), q (quartet), dd (double doublet), ddd (double double doublet), and m (multiplet). High-resolution mass spectra (HRMS, ESI^+^) were acquired on a Bruker Maxis Impact Q-TOF spectrometer (Bruker, Billerica, MA). The purity of compound **1** was determined by HPLC using a Waters chromatograph equipped with a photodiode array (PDA) detector (Waters Corporation, MA). The mobile phase consisted of water (A) and acetonitrile (B), with a gradient elution starting at 95:5 (A/B) and linearly increasing to 5:95 (A/B) over 15 min. For further details, please refer to the Supporting Information, where the spectra for all analytical methods are described.

### 4.2 General Procedure for the Synthesis of Dipeptidyl Nitrile Derivatives

Dipeptidyl nitrile derivatives were obtained following a procedure adapted from Avelar et al. (2015).^31^ The route A (Scheme 1) describes the general synthesis of compounds **1–9**. The Boc-protected amino acids: Boc-l-phenylalanine, Boc-l-leucine, Boc-l-tyrosine, Boc-L-4-fluorophenylalanine, and Boc-L-3,4-difluorophenylalanine, as well as the amino-nitrile warheads aminoacetonitrile and aminocyclopropanecarbonitrile, were obtained from commercial suppliers.

#### 4.2.1 General Procedure

Boc-L-amino acids (1.13 mmol) in a solution of anhydrous dimethylformamide (DMF, 5 mL) were treated with *N*,*N*-Diisopropylethylamine (DIPEA, 2.25 mmol) and 1-[Bis­(dimethylamino)­methylene]-1H-1,2,3-triazolo­[4,5-*b*]­pyridinium 3-oxide hexafluorophosphate (HATU, 1.47 mmol). Subsequently, the respective amino-nitriles warheads (1.47 mmol) were added to the reaction medium. The reaction was maintained at room temperature, under an inert atmosphere (N_2_), for 48 h. After this time, 50 mL of water was added to the reaction medium, and the resulting solution was washed with ethyl acetate (3 × 50 mL) in a separatory funnel. The organic phase was collected and concentrated under reduced pressure. The resulting products were purified by column chromatography with hexane and ethyl acetate as eluents (50:50 v/v, isocratic mode).

#### 4.2.2 (S)-*tert*-Butyl (1-((Cyanomethyl)­amino)-1-oxo-3-phenylpropan-2-yl)­carbamate - **1**

White solid; yield 40%; ^1^H NMR (600 MHz, DMSO-*d*_6_) δ 8.66 (t, *J* = 5.7 Hz, 1H, N–H amide), 7.30–7.23 (m, 4H, phenyl ring), 7.22–7.17 (m, 1H, phenyl ring), 7.07 (d, *J* = 8.5 Hz, 1H, N–H carbamate), 4.19–4.11 (m, 1H, aliphatic H), 4.14 (d, *J* = 5.7 Hz, 2H, CH_2_ nitrile), 2.96 (dd, *J* = 13.7, 4.7 Hz, 1H, CH_2_ phenyl ring), 2.76 (dd, *J* = 13.7, 10.3 Hz, 1H, CH_2_ phenyl ring), 1.30 (s, 9H, Boc group). ^13^C NMR (150 MHz, DMSO) δ 172.39, 155.27, 137.89, 129.15, 128.06, 126.26, 117.51, 78.13, 55.57, 37.14, 28.12, 27.11. HRMS (ESI^+^, methanol) calculated: C_16_H_21_N_3_O_3_ [M^+^H]^+^ = 304.1656, found: 304.1652. Purity by HPLC: peak area >96% (Figures S10–S14, Supporting Information).

#### 4.2.3 (S)-*tert*-Butyl (1-((Cyanomethyl)­amino)-4-methyl-1-oxopentan-2-yl)­carbamate - **2**

White solid; yield 35%; ^1^H NMR (600 MHz, DMSO-*d*_6_) δ 8.55 (t, *J* = 5.7 Hz, 1H, N–H amide), 7.00 (d, *J* = 8.2 Hz, 1H, N–H carbamate), 4.10 (dd, *J* = 5.6, 1.9 Hz, 2H, CH_2_ nitrile), 3.99–3.92 (m, 1H, aliphatic H), 1.62–1.53 (m, 1H, CH_2_ isopropyl), 1.49–1.41 (m, 1H, CH_2_ isopropyl), 1.37 (s, 9H, Boc group), 1.36–1.34 (m, 1H, H-isopropyl), 0.87 (d, *J* = 6.6 Hz, 3H, CH_3_-isopropyl), 0.84 (d, *J* = 6.6 Hz, 3H, CH_3_-isopropyl). ^13^C NMR (150 MHz, DMSO) δ 173.35, 155.41, 117.62, 78.10, 52.47, 40.40, 28.19, 27.09, 24.25, 22.91, 21.40. HRMS (ESI^+^, methanol) calculated: C_13_H_23_N_3_O_3_ [M^+^H]^+^ = 270.1812, found: 270.1814. (Figures S15–S18).

#### 4.2.4 (S)-*tert*-Butyl (1-((Cyanomethyl)­amino)-3-(4-hydroxyphenyl)-1-oxopropan-2-yl)­carbamate - **3**

White solid; yield 12%; ^1^H NMR (600 MHz, DMSO-*d*_6_) δ 9.16 (s, 1H, OH), 8.59 (t, *J* = 5.6 Hz, 1H, N–H amide), 7.01 (d, *J* = 8.5 Hz, 2H, phenyl ring), 6.96 (d, *J* = 8.4 Hz, 1H, N–H carbamate), 6.64 (d, *J* = 8.5 Hz, 2H, phenyl ring), 4.11 (d, *J* = 5.6 Hz, 2H, CH_2_ nitrile), 4.07–4.01 (m, 1H, aliphatic H), 2.82 (dd, *J* = 13.8, 4.9 Hz, 1H, CH_2_ phenyl ring), 2.63 (dd, *J* = 13.8, 9.8 Hz, 1H, CH_2_ phenyl ring), 1.31 (s, 9H, Boc group).^13^C NMR (150 MHz, DMSO) δ 172.55, 155.80, 155.26, 130.06, 127.83, 117.51, 114.87, 78.11, 55.92, 36.42, 28.15, 27.08. HRMS (ESI^+^, methanol) calculated: C_16_H_21_N_3_O_4_ [M^+^H]^+^ = 320.1605, found: 320.1609. (Figures S19–S22).

#### 4.2.5 (S)-*tert*-Butyl (1-((Cyanomethyl)­amino)-3-(4-fluorophenyl)-1-oxopropan-2-yl)­carbamate - **4**

White solid; yield 67%, ^1^H NMR (600 MHz, DMSO-*d*_6_) δ 8.65 (t, *J* = 5.7 Hz, 1H, N–H amide), 7.27 (dd, *J* = 8.2, 5.8 Hz, 2H, phenyl ring), 7.12–7.02 (m, 3H), 4.18–4.08 (m, 3H), 2.94 (dd, *J* = 13.7, 4.7 Hz, 1H, CH_2_ phenyl ring), 2.73 (dd, *J* = 13.7, 10.2 Hz, 1H, CH_2_ phenyl ring), 1.29 (s, 9H, Boc group). ^13^C NMR (150 MHz, DMSO) δ 172.31, 161.05 (d, *J*_C–F_ = 241.6 Hz), 155.34, 134.08, 131.07 (d, *J*_C–F_ = 7.9 Hz), 117.57, 114.81 (d, *J*_C–F_ = 21.0 Hz), 78.22, 55.66, 36.38, 28.16, 27.16. HRMS (ESI^+^, methanol) calculated: C_16_H_20_FN_3_O_3_ [M^+^H]^+^ = 322.1562, found: 322.1567. (Figures S23–S26).

#### 4.2.6 (S)-*tert*-Butyl (1-((Cyanomethyl)­amino)-3-(3,4-difluorophenyl)-1-oxopropan-2-yl)­carbamate - **5**

White solid; yield 42%,^1^H NMR (600 MHz, DMSO-*d*_6_) δ 8.65 (t, *J* = 5.7 Hz, 1H, N–H amide), 7.38–7.25 (m, 2H, phenyl ring), 7.13 (d, *J* = 8.7 Hz, 1H, N–H carbamate), 7.10–7.06 (m, 1H, phenyl ring), 4.19–4.08 (m, 3H), 2.96 (dd, *J* = 13.7, 4.7 Hz, 1H, CH_2_ phenyl ring), 2.72 (dd, *J* = 13.7, 10.5 Hz, 1H, CH_2_ phenyl ring), 1.29 (s, 9H, Boc group).^13^C NMR (150 MHz, DMSO-*d*_6_) δ 171.98, 155.28, 149.75 (d, *J*_C–F_ = 12.5 Hz), 149.02 (d, *J*_C–F_ = 12.4 Hz), 148.13 (d, *J*_C–F_ = 12.5 Hz), 147.41 (d, *J*_C–F_ = 12.5 Hz), 135.75, 126.05, 118.10 (d, *J*_C–F_ = 16.7 Hz), 117.49, 116.95 (d, *J* = 16.7 Hz), 78.22, 55.31, 36.25, 28.04, 27.11. HRMS (ESI^+^, methanol) calculated: C_16_H_19_F_2_N_3_O_3_ [M^+^H]^+^ = 340.1467 found: 340.1467. (Figures S27–S30).

#### 4.2.7 (S)-*tert*-Butyl (1-((1-Cyanocyclopropyl)­amino)-1-oxo-3-phenylpropan-2-yl)­carbamate - **6**

White solid; yield 37%,^1^H NMR (600 MHz, DMSO-*d*_6_) δ 8.85 (s, 1H, N–H amide), 7.29–7.15 (m, 5H, phenyl ring), 7.00 (d, *J* = 8.3 Hz, 1H, N–H carbamate), 4.05 (m, 1H, aliphatic H), 2.87 (dd, *J* = 13.6, 5.7 Hz, 1H, CH_2_ phenyl ring), 2.75 (dd, *J* = 13.6, 9.3 Hz, 1H, CH_2_ phenyl ring), 1.45–1.40 (m, 2H, cyclopropyl nitrile), 1.31 (s, 9H), 1.00–0.94 (m, 2H, cyclopropyl nitrile). ^13^C NMR (150 MHz, DMSO) δ 173.03, 155.22, 137.63, 129.26, 128.10, 126.36, 120.73, 78.20, 55.46, 37.28, 28.15, 19.66, 15.69. HRMS (ESI^+^, methanol) calculated: C_18_H_23_N_3_O_3_ [M^+^H]^+^ = 330.1812 found: 330.1814. (Figures S31–S34).

#### 4.2.8 (S)-*tert*-Butyl (1-((1-Cyanocyclopropyl)­amino)-4-methyl-1-oxopentan-2-yl)­carbamate - **7**

White solid; yield 19%. ^1^H NMR (600 MHz, DMSO-*d*_6_) δ 8.80 (s, 1H, N–H amide), 6.92 (d, *J* = 8.1 Hz, 1H, N–H carbamate), 3.86 (ddd, *J* = 9.7, 8.1, 5.1 Hz, 1H, aliphatic H), 1.58–1.51 (m, 1H, H-isopropyl), 1.48–1.43 (m, 2H, cyclopropyl nitrile), 1.37 (s, 9H, Boc group), 1.10–1.05 (m, 2H, cyclopropyl nitrile), 0.85 (d, *J* = 6.6 Hz, 3H, CH_3_-isopropyl); 0.83 (d, *J* = 6.6 H, 3H,CH_3_-isopropyl).^13^C NMR (150 MHz, DMSO) δ 174.07, 155.38, 120.82, 78.08, 52.41, 40.34, 28.18, 24.24, 22.90, 21.52, 15.74, 15.61. HRMS (ESI^+^, methanol) calculated: C_15_H_25_N_3_O_3_ [M^+^H]^+^ = 296.1974 found: 296.1974. (Figures S35–S38).

#### 4.2.9 (S)-*tert*-Butyl (1-((1-Cyanocyclopropyl)­amino)-3-(4-fluorophenyl)-1-oxopropan-2-yl)­carbamate - **8**

White solid; yield 60%.^1^H NMR (600 MHz, DMSO-*d*_6_) δ 8.86 (s, 1H, N–H amide), 7.27–7.21 (m, 2H, phenyl ring), 7.11–7.06 (m, 2H, phenyl ring), 7.03 (d, *J* = 8.3 Hz, 1H, N–H carbamate), 4.02 (ddd, *J* = 9.7, 7.3, 5.4 Hz, 1H, aliphatic H), 2.86 (dd, *J* = 13.6, 5.4 Hz, 1H, CH_2_ phenyl ring), 2.73 (dd, *J* = 13.6, 9.6 Hz, 1H, CH_2_ phenyl ring), 1.49–1.40 (m, 2H, cyclopropyl nitrile), 1.30 (s, 9H, Boc group), 1.02–0.99 (m, 2H, cyclopropyl nitrile). ^13^C NMR (151 MHz, DMSO) δ 172.85, 160.99 (d, *J*_C–F_ = 241.4 Hz), 155.18, 133.80, 131.03 (d, *J*_C–F_ = 7.8 Hz) 120.67, 114.72 (d, *J*_C–F_ = 21.0 Hz), 78.13, 55.44, 36.34, 28.08, 15.65. HRMS (ESI^+^, methanol) calculated:C_18_H_22_FN_3_O_3_ [M^+^H]^+^ = 348.1723 found: 348.1721. (Figures S39–S42).

#### 4.2.10 (S)-*tert*-Butyl (1-((1-Cyanocyclopropyl)­amino)-3-(3,4-difluorophenyl)-1-oxopropan-2-yl)­carbamate - **9**

White solid; yield 40%,^1^H NMR (600 MHz, DMSO-*d*_6_) δ 8.87 (s, 1H, N–H amide), 7.36–7.29 (m, 1H, phenyl ring), 7.29–7.24 (m, 1H, phenyl ring), 7.09–7.03 (m, 2H), 4.09–3.97 (m, 1H, 1H, aliphatic H), 2.89 (dd, *J* = 13.6, 5.2 Hz, 1H, CH_2_ phenyl ring), 2.72 (dd, *J* = 13.6, 9.8 Hz, 1H, CH_2_ phenyl ring), 1.49–1.43 (m, 2H, cyclopropyl nitrile), 1.29 (s, 9H, Boc group), 1.06–1.01 (m, 2H, cyclopropyl nitrile). ^13^C NMR (151 MHz, DMSO) δ 172.63, 155.19, 149.73 (d, *J*_C–F_ = 12.3 Hz), 149.03 (d, *J*_C–F_ = 12.3 Hz), 148.11 (d, *J*_C–F_ = 12.5 Hz), 147.41 (d, *J*_C–F_ = 12.3 Hz), 135.55, 126.05, 120.66, 118.13 (d, *J*_C–F_ = 16.7 Hz), 116.95 (d, *J*_C–F_ = 16.7 Hz). 78.20, 55.20, 36.28, 28.04, 19.68, 15.69. HRMS (ESI^+^, methanol) calculated: C_18_H_21_F_2_N_3_O_3_ [M^+^H]^+^ = 366.1629 found: 366.1615. (Figures S43–S46).

### 4.3 Molecular Docking

All computational analyses were carried out within the Maestro (Schrödinger) molecular modeling environment, following the methodology previously described.^26^ Three-dimensional structures were generated using the *LigPrep* workflow. Protonation states at pH 5.5 ± 1.0—consistent with the conditions used in CPB enzymatic assays—were predicted with *Epik*,^71^ and the OPLS3 force field was applied throughout the preparation. The structure of the target protein, PDB code: 6P4E, with a resolution of 1.35 Å^36^ was obtained from the Protein Data Bank and prepared using the *Protein Preparation Wizard*. During this process, hydrogen atoms were initially removed and then reintroduced, missing residues were modeled, and all residues were optimized according to the specified pH range. A docking grid was generated to encompass all potential binding sites, with the catalytic residues CYS26 and HIS164 set as deprotonated and protonated, respectively. The resulting grid box, centered on the catalytic cysteine, had dimensions of 15 Å. Molecular docking studies were conducted using *Covalent Docking Mode* and the *Pose Prediction mode*, generating 3 poses per ligand along with postdocking minimization. All other parameters were kept at their default settings. Validation of the docking protocol was performed based on the RMSD value obtained (0.15 Å), which resulted from multiple minimization steps during the protein preparation.

### 4.4 Parasite Cultures and Macrophage Isolation

Promastigote forms of *L. amazonensis* (MHOM/BR/71973/M2269) were propagated at 26 °C in liver infusion tryptose (LIT) medium, enriched with 10% heat-inactivated fetal bovine serum (FBS) (Nova Biotecnologia) and 1% antibiotic solution containing streptomycin (10 mg/mL) and penicillin (10,000 U/mL) (Nova Biotecnologia), using 25 cm^2^ sterile culture flasks (TPP). For *L. infantum* (MHOM/MA67ITMAP263), parasites were cultivated under the same temperature conditions in M199 medium supplemented with 10% heat-inactivated FBS, 1% antibiotic mix (streptomycin 10 mg/mL and penicillin 10,000 U/mL) (Nova Biotecnologia), and 10% sterile male human urine. The culture conditions were adapted from the methodology previously described.^27,72^

Murine peritoneal macrophages were harvested following the methodology previously described.^72^ Briefly, male Swiss mice (6–8 weeks old) received an intraperitoneal injection of 3% thioglycolate solution (HIMEDIA). After 72 h, cells were collected by injecting phosphate-buffered saline (PBS, pH 7.4) into the peritoneal cavity, followed by gentle abdominal massage and aspiration of the peritoneal fluid using a sterile syringe. The harvested cells were seeded in RPMI 1640 medium (Sigma-Aldrich) supplemented with 10% heat-inactivated FBS (Nova Biotecnologia), 25 mM HEPES (Sigma-Aldrich), 2 mM l-glutamine (Sigma-Aldrich), and 1% penicillin/streptomycin (Nova Biotecnologia), and maintained at 37 °C with 5% CO_2_ in either 96-well or 24-well plates, depending on the experimental requirements.

### 4.5 Evaluation of Cytotoxicity on Peritoneal Macrophages

Peritoneal macrophages were obtained as described above according to.^72^ After isolation, cells were seeded into 96-well plates at a density of 5 × 10^5^ cells/mL in RPMI-1640 medium (Sigma-Aldrich) and incubated at 37 °C with 5% CO_2_ for 6 h to allow adhesion. Following incubation, nonadherent cells were removed by discarding the supernatant, and adherent macrophages were treated with RPMI-1640 medium (Sigma-Aldrich) containing test compounds at concentrations ranging from 300 to 2.34 μM. Wells containing only RPMI-1640 medium (Sigma-Aldrich) served as untreated controls. Plates were then incubated under the same conditions for 24 h. Cell viability was assessed using the MTT assay. After discarding the supernatant, 6 mM MTT­(Sigma-Aldrich) and 0.7 mM PMS (Sigma-Aldrich) in PBS were added to each well, followed by incubation for 75 min maintained under light-restricted conditions. Formazan crystals were solubilized using ethanol and isopropanol/PBS (1:1, v/v), and absorbance was measured at 570 nm using a UV/vis spectrophotometer (Tecan). The 50% cytotoxic concentration (CC_50_) was determined by converting optical density values to percentage inhibition and performing nonlinear regression analysis. Results are expressed as mean ± SD from two independent experiments, with each experiment conducted in triplicate.

### 4.6 Evaluation of Antiamastigote Activity

The antiamastigote activity of the compounds was evaluated following the methodology previously described,^73,74^ with some modifications detailed below. Murine peritoneal macrophages were harvested and maintained for 6 h in RPMI-1640 medium (Sigma-Aldrich) at 37 °C with 5% CO_2_, distributed in 24-well plates to allow adherence onto coverslips placed within the wells. After adhesion, cells were infected with stationary-phase promastigotes of the respective *Leishmania* species at a parasite-to-macrophage ratio of 5:1 for *L. amazonensis* and 10:1 for *L. infantum*. Infections were allowed to proceed for 18 h at 37 °C in a humidified atmosphere containing 5% CO_2_. After infection, wells were washed with PBS to remove noninternalized parasites, and cells were treated for 24 h with compounds at serial dilutions ranging from 30 μM to 1.875 μM (1:2 ratio). Subsequently, the supernatant was removed, and coverslips were air-dried at room temperature. Cells were fixed with methanol and stained with 10% Giemsa (MERCK) in PBS for 8 min. The EC_50_ for antiamastigote activity was determined by counting the number of internalized amastigotes per 100 macrophages under light microscopy. Treated samples were compared to untreated infected controls. The infection index was calculated by multiplying the percentage of infected macrophages by the mean number of intracellular amastigotes *per* infected macrophage and was used to evaluate treatment effects at different concentrations.^89^ The results are presented in the Supporting Information. All experiments were conducted in triplicate, including both biological and technical replicates, and the data are presented as mean ± SD.

### 4.7 Calculation of the Selectivity Index (SI)

The selectivity index (SI) was calculated as the ratio of the 50% cytotoxic concentration (CC_50_) in murine peritoneal macrophages to the 50% effective concentration (EC_50_) against intracellular *Leishmania* amastigotes (SI = CC_50_/EC_50_). The SI was used as an indicator of compound selectivity and safety, with a value of ≥ 10 considered indicative of a favorable selectivity profile and consistent with the hit and lead selection criteria established by the *DNDi.* According to the criteria proposed by Katsuno et al. (2015), an EC_50_ value below 10 μM against intracellular amastigotes, together with an SI of ≥ 10, supports the prioritization of compounds for further antileishmanial drug development.^47^

### 4.8 Evaluation of Inhibitory Activity against the CPB Enzyme

The *C*-terminally truncated recombinant cysteine protease *Lm*CPB2.8ΔCTE was expressed, and its enzymatic inhibition was evaluated according to the methodology previously described,^26^ with minor modifications. The enzymatic inhibition assay was conducted in black 96-well microplates using a reaction mixture composed of 100 mM sodium acetate buffer (pH 5.5), supplemented with 200 mM NaCl (SYNTH), 0.01% Triton X-100 (Sigma-Aldrich), 2 nM of the recombinant cysteine protease *Lm*CPB2.8ΔCTE (rCPB2.8), and 2 mM dithiothreitol (DTT) (Acros Organics), in a final volume of 196 μL. The enzyme was preincubated at 30 °C for 2 min, after which 2 μL of the test molecules, prepared at various concentrations (1.25–20 μM), were added. Following a brief incubation, 2 μL of the fluorogenic substrate Z-Phe-Arg-7-amido-4-methylcoumarin (Z-FR-AMC, 5 μM; Calbiochem) was added to initiate the reaction.

Fluorescence generated by substrate hydrolysis—indicative of enzymatic activity—was monitored for 2 min at excitation/emission wavelengths of 380/460 nm using a microplate reader (Tecan). Control wells included reactions without enzyme (to assess basal fluorescence) and without inhibitors (to determine maximal enzymatic activity). The residual enzyme activity was calculated by comparing fluorescence values of wells containing test molecules to those of the uninhibited controls, with subtraction of background fluorescence in both cases. Molecules that demonstrated ≥ 50% inhibition at 20 μM were further evaluated in dose–response assays across a concentration range of 1.25–20 μM. The half-maximal inhibitory concentration (IC_50_-CPB) was determined through nonlinear regression analysis using GraphPad Prism. Results are expressed as mean ± SD from two independent experiments, with each experiment conducted in triplicate.

### 4.9 Determination of the CPB Enzyme Inhibition Profile

The enzyme kinetics assay aims to elucidate the inhibition mechanism of *Leishmania* cysteine protease B enzyme, indicating the possibility of competitive, mixed, or uncompetitive inhibition. The methodology previously described^75^ was used with minor modifications. In summary, the assay is performed in 96-well black plates, using a reaction medium initially composed of: 100 mM sodium acetate buffer (pH 5.5), supplemented with 200 mM NaCl (SYNTH), 0.01% Triton X-100 (Sigma-Aldrich), recombinant cysteine protease *Lm*CPB2.8ΔCTE 2 nM (rCPB2.8), and 2 mM dithiothreitol (DTT) (Acros Organics), in a final volume of 98 μL. A volume of 2 μL of the inhibitor at a fixed concentration was added for each reading. As an evaluation strategy, the inhibitor concentration range was analyzed at three points: the IC_50_ value, a concentration ten times higher, and a concentration ten times lower. Different concentrations (20 μM; 15 μM; 10 μM; 7.5 μM; 5 μM; 2.5 μM; 1 μM and 0.5 μM) of the fluorogenic substrate Z-Phe-Arg-7-amido-4-methylcoumarin (Z-FR-AMC, Calbiochem) were added in a final volume of 100 μL to initiate the reaction. The reaction rate was measured using an Infinite 200 PRO microplate reader (Tecan) through the fluorescence generated by the substrate hydrolysis, indicative of the enzymatic activity monitored through excitation/emission at 380/460 nm. It was possible to calculate the initial substrate hydrolysis rates using GraphPad Prism software based on the linear regression coefficient of the Relative Fluorescence Unit (mRFU) data adjustment as a function of time (min). A control measurement in the absence of inhibitor (*K*_m_) was also performed for each plate. Finally, the data were expressed using a double reciprocal plot (Lineweaver–Burk diagram), also analyzing the *K*_m_ and Vmax values to determine the type of inhibition that occurred.

### 4.10 *In Vitro* ADME Assays

The *in vitro* ADME assays were carried out according to previously reported methodologies, with minor adaptations.^76,77^

### 4.11 Parallel Artificial Membrane Permeability Assay (PAMPA)

The passive permeability of the selected compounds was evaluated by PAMPA using a 96-well donor/receiver plate system. For the gastrointestinal (GI)-PAMPA assay, 10 mM stock solutions of the compounds were prepared in DMSO and diluted with PBS buffer (pH 6.6, 10 mM). After filtration through a 0.45 μm PVDF filter, aliquots of the resulting solutions were added to the donor plate previously coated with L-α-phosphatidylcholine in dodecane, while the receiver plate contained PBS (pH 7.4)/DMSO (95:5). The donor plate was placed over the receiver plate and incubated under stirring (50 rpm) for 8 h at room temperature in a sealed vessel. After incubation, the contents of the receiver plate were transferred to a UV plate and analyzed using a SpectraMax 5 microplate reader (Molecular Devices). The optical density values obtained for the receiver plate were compared with those of the initial compound solutions, and permeability values were determined using calibration with reference compounds of known permeability. All measurements were performed in triplicate and in at least two independent experiments. For the blood–brain barrier (BBB)-PAMPA assay, compounds were prepared in PBS (pH 7.4)/ethanol, the donor plate was coated with porcine brain lipid in dodecane, and the donor/receiver sandwich was incubated for 2 h 45 min at room temperature under sealed conditions. Permeability values were determined analogously using appropriate reference standards.^76,77^

### 4.12 Metabolic Stability in Human and Mouse Liver Microsomes (HLM and MLM)

The metabolic stability of selected compounds was evaluated in human and mouse liver microsomes in the presence of an NADPH-generating system at pH 7.4 and 37 °C. Test compounds were incubated with liver microsomal protein from the species of interest, and reactions were initiated by the addition of NADPH after the collection of the T0 sample. Aliquots were collected at predetermined time points and quenched with acetonitrile/methanol containing an internal standard. Samples were analyzed by LC-MS/MS, and peak area ratios (analyte/internal standard) were converted to the percentage of compound remaining, with the signal at time zero set to 100%. Half-life values were calculated as *t*_1/2_ = ln(2)/*k*, and intrinsic clearance was calculated as CLint = *k* × 1000/protein concentration, expressed as μL/min/mg protein, where *k* corresponds to the slope obtained from the plot of percentage remaining versus incubation time. Data were considered qualified when the half-life exceeded four times the last time point analyzed. In additional microsomal stability experiments, the incubation medium comprised mouse liver microsomes, phosphate buffer (pH 7.4), and an NADPH-generating system containing NADP^+^, MgCl_2_, glucose-6-phosphate, and glucose-6-phosphate dehydrogenase. Reactions were stopped by the addition of chilled methanol and acetonitrile, followed by protein precipitation, centrifugation, filtration through a PVDF membrane, and HPLC-PDA analysis. Control incubations included samples without the test compound, without the NADPH-generating system, and without the microsomal fraction to distinguish enzymatic turnover from nonenzymatic instability.^76,77^

### 4.13 Experimental Lipophilicity Determination [eLogD7.4]

Experimental lipophilicity was determined by LC-MS/MS using a Prominence UFLC system (Shimadzu, Kyoto, Japan) coupled to an LCMS-8045 triple quadrupole mass spectrometer equipped with an electrospray ionization source. Chromatographic separation was carried out on a Supelco Ascentis Express RP-Amide column using 5% methanol in 10 mM ammonium acetate buffer (pH 7.4) as mobile phase A and methanol as mobile phase B under gradient elution. Test compounds were prepared from stock solutions in a 1:1 mixture of mobile phases A and B, with DMSO concentration kept below 2%. A calibration curve was generated using eight commercial standards spanning a log­(D) range from −1.86 to 6.10. The retention times of the standards were plotted against their known log­(D) values, and the resulting linear equation was used to calculate the experimental log­(D) [eLogD7.4] values of the compounds.^76,77^

### 4.14 Kinetic Solubility

Kinetic solubility was determined by LC-MS/MS using a Prominence UFLC system (Shimadzu, Kyoto, Japan) coupled to an LCMS-8045 triple quadrupole mass spectrometer equipped with an electrospray ionization source. Stock solutions of test compounds and controls (10 mM in DMSO) were transferred in duplicate to 96-well incubation plates, and PBS at pH 2.0 or 7.4 was added to give a final compound concentration of 250 μM while maintaining the DMSO concentration below 2.5%. Plates were sealed and shaken for 24 h at 25 °C. After centrifugation, the supernatants were quantified by LC-MS/MS using calibration curves prepared individually for each compound. Chromatographic analysis was performed on a Supelco Ascentis Express C18 column using water containing 0.05% formic acid as mobile phase A and acetonitrile containing 0.05% formic acid as mobile phase B under gradient elution. Experimental concentrations were calculated from the calibration curves and used as the kinetic solubility values for each compound at both pH conditions.^76,77^

### 4.15 Evaluation of *In Vivo* Systemic Toxicity in the *G. mellonella* Model

The evaluation of *in vivo* systemic toxicity in *G. mellonella* was performed according to the methodology previously described,^78^ with minor modifications. Light-colored larvae without melanization, measuring 2–3 cm in length and weighing between 180–300 mg, were selected and incubated at 37 °C without food for 24 h prior to the experiment. The larvae were randomly divided into three groups of ten individuals each and injected into the right proleg using an insulin syringe with 20 μL of the respective solutions: PBS (G1), PBS with 6% DMSO (G2), and compound **1** (2.8 mM) in 6% DMSO (G3). After treatment, the larvae were maintained at 37 °C in Petri dishes and monitored daily for 5 days. Health status was evaluated based on the Health Index Scoring System (1–10), which considers larval mobility, cocoon formation, melanization, and survival.^79^ The results were statistically analyzed using GraphPad Prism software in at least three independent experiments.

### 4.16 Evaluation of *In Vivo* Efficacy and Safety of Compound 1 in a Murine Model of Visceral Leishmaniasis

The *in vivo* antileishmanial efficacy of compound **1** was evaluated using a murine model of visceral leishmaniasis. Male BALB/c mice (6 weeks old, 27 ± 1 g; CEMIB-UNICAMP) were infected intraperitoneally with 100 μL (1 × 10^8^) stationary-phase *L. infantum* promastigotes (MHOM/MA67ITMAP263). After 15 days of infection progression, animals were treated by oral gavage once daily for five consecutive days. Compound **1**, evaluated in an exploratory *in vivo* assay, was administered at 0.77 mg/kg/day, corresponding to one-tenth of the reference dose of miltefosine (7.7 mg/kg/day; Cayman Chemical). This experimental design aimed to determine whether the precursor chemical scaffold retained antileishmanial activity at a low dose, providing a proof-of-concept for the potential of the proposed pharmacophore. Miltefosine was administered following the same treatment schedule at 7.7 mg/kg/day as the reference drug.^26^ Negative controls consisted of infected, untreated mice and infected mice treated with PBS as the vehicle control. Working solutions were freshly prepared in PBS prior to administration. Miltefosine-treated animals served as the positive control group. At the end of treatment, mice were euthanized, and liver and spleen tissues were harvested for parasite burden quantification. In addition, cardiac blood was also collected posteuthanasia to assess systemic toxicity. Biochemical markers of hepatic and renal function—AST, ALT, ALP, creatinine, urea, and total bilirubin—were analyzed. Imprints of tissues collected were prepared on glass slides, stained with Giemsa, and analyzed under light microscopy. Parasite load was determined by Leishman Donovan Units (LDU), calculated as the number of amastigotes per 1,000 host cell nuclei per gram of tissue, and by the limiting dilution methodology as previously described.^72,80,81,88^ Results for parasite burden were expressed as mean ± SEM.

### 4.17 Determination of Hemolytic Activity

Hemolytic activity can be expressed as the ability to lyse 50% of red blood cells (HC_50_); it was determined from blood collected in a hematology tube containing EDTA. The protocol followed was described previously.^82^ In summary, red blood cells were washed 3 times with PBS buffer, then centrifuged, the supernatant discarded, and the cells resuspended in PBS at a concentration of 1% (g/v). Different experimental conditions related to compound **1** were investigated: Samples were incubated at 37 °C for 1 h, then centrifuged. The supernatant was collected and transferred to a 96-well microplate. Hemolytic activity in human blood after inoculation with compound **1** at 3.69 mM, corresponding to ten times the value used in the *in vivo* evaluation (0.77 mg/kg/day or 0.369 mM), was evaluated. Triton was used as the positive control, while the vehicle (PBS containing 6% DMSO) was used as the negative control. The analysis was performed by measuring the absorbance at 540 nm in a plate reader (Tecan). The entire assay was performed in duplicate. The Research Ethics Committee approved this project under the number 90291518.4.0000.5426.

### 4.18 Ethics Statement

Experiments using animals were authorized by the Animal Experimentation Ethics Committee of the São Paulo State University (UNESP), the School of Pharmaceutical Sciences (CEUA 03/19), in accordance with the guidelines of the National Council for the Control of Animal Experimentation (CONCEA) and the Sociedade Brasileira de Ciência de Animais de Laboratório (SBCAL).

### 4.19 Statistical Analysis

Statistical analyses were evaluated using one-way analysis of variance followed by Tukey’s multiple comparisons test (using GraphPad InStat software). Differences were considered significant when *P* < 0.05.

## Supplementary Material



## Notes

Supporting results from the molecular docking study; infection indices for *L. infantum* and *L. amazonensis* amastigotes treated with compound **1**; enzyme kinetics for compounds **2** and **6**; determination of the hemolytic activity of compound **1**; toxicity in *G. mellonella*; and NMR analysis of the nitrile-based series (PDF)
