# Supplementary material for: Nitrile-Based Leishmania Cysteine Protease B Inhibitors with In Vivo Efficacy against Visceral Leishmaniasis
Source: ACS Omega. 2026 Oct 22;11(43):66899–915. doi: 10.1021/acsomega.6c06826 (PMC13737875; doi:10.1021/acsomega.6c06826)
Supplement: Supplementary file 1 [file ao6c06826_si_001.pdf]

SUPPORTING INFORMATION

Nitrile-Based *Leishmania* Cysteine Protease B Inhibitors with *In Vivo* Efficacy against Visceral Leishmaniasis

Jhonatan Santos de Lima[1,‡]; Michele Ferreira da Silva Mela[1,‡]; Juliana Romano Lopes[2]; João Lucas Bruno Prates [2]; Natalia Caroline Costa Coelho[1]; Ana Clara Lunardi Yagi[1]; Rafael Consolin Chelucci[3]; Simone Michelan Duarte[3]; Leonardo Luiz Gomes Ferreira[3]; Adriano Defini Andricopulo[3]; Gustavo Chagas Paulino[2]; João Victor Marcelino De Souza[4]; Bruna Cardinali Lustri Morgado[5]; Paulo Ricardo da Silva Sanches[5]; Eduardo Maffud Cilli[4]; Jean Leandro Dos Santos[2,*]; Angela Maria Arenas Velásquez[1,6,*]; Marcia Aparecida Silva Graminha[1,*]

[1]Department of Clinical Analysis, School of Pharmaceutical Sciences, São Paulo State University (UNESP), Araraquara 14800-903, Brazil;

[2]Department of Drugs and Medicines, School of Pharmaceutical Sciences, São Paulo State University (UNESP), Araraquara 14800-903, Brazil;

[3]Laboratory of Medicinal and Computational Chemistry, Physics Institute of São Carlos, University of São Paulo (USP), São Carlos 13563-120, SP, Brazil;

[4]Department of Biochemistry and Organic Chemistry, Institute of Chemistry, São Paulo State University (UNESP), Araraquara 14800-060, Brazil;

[5]Department of Biological Sciences, School of Pharmaceutical Sciences, São Paulo State University (UNESP), Araraquara 14800-903, Brazil;

[6]Latin American Institute of Life and Nature Sciences, Federal University of Latin American Integration (UNILA), Foz do Iguaçu, Brazil, 85870-650

‡**Authors contributed equally**

*Corresponding author: MASG, AMAV, and JLDS.

Supplementary results from the molecular docking study; infection indices for *Leishmania infantum* and *Leishmania amazonensis* amastigotes treated with compound **1**; enzyme kinetics for compounds **2** and **6**; determination of the hemolytic activity of compound **1**; toxicity in *Galleria mellonella*; and NMR analysis of the nitrile-based series.

RESULTS AND DISCUSSION

Synthesis and Molecular Docking of Dipeptidyl Nitrile Derivatives

Table S1. Docking Score (DS) and Molecular Mechanics Generalized Born Surface Area (MMGBSA) values calculated for the best binding pose of each compound (**1–9**), and for controls: K777 and E64.

Compound	DS	MMGBSA
**1**	-6.290	-60.63
**2**	-3.819	-59.89
**3**	-6.313	-61.27
**4**	-6.709	-61.94
**5**	-6.549	-58.78
**6**	-6.427	-62.82
**7**	-4.541	-61.51
**8**	-6.408	-64.10
**9**	-6.173	-64.72
**K777**	-7.155	-74.13
**E64**	-7.098	-80.17

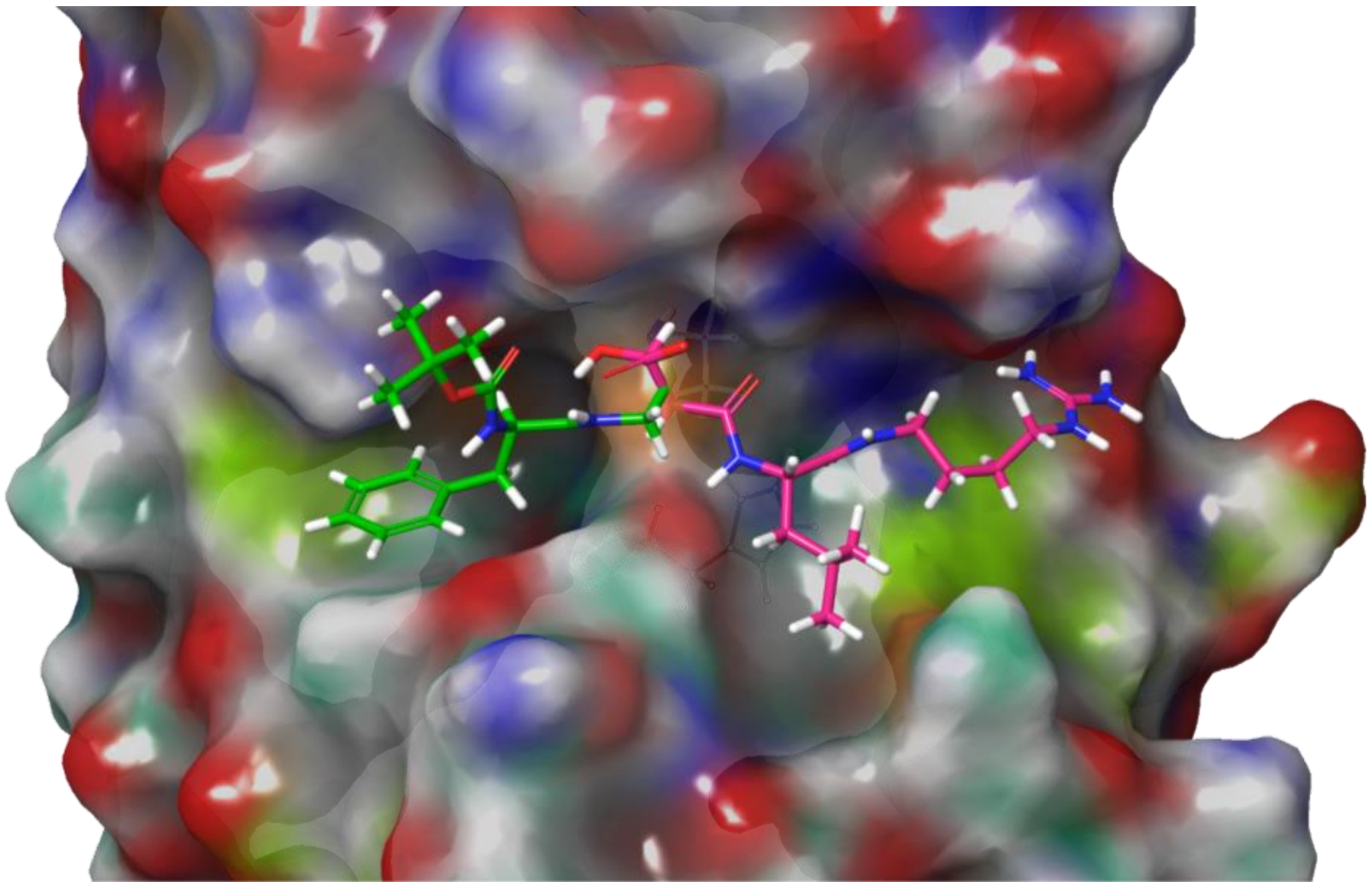

Figure S1. The 3D representation shows that the best docking poses of compound **1** (green) and E64 (pink) interact with different regions of the CPB protease. Compound **1** is restricted to the S1, S2, and S3 regions, whereas E64 interacts with the S1, S1′, and S4 regions.

Identification of Reversible Covalent CPB Inhibitors and *In Vitro* Antileishmanial Activity

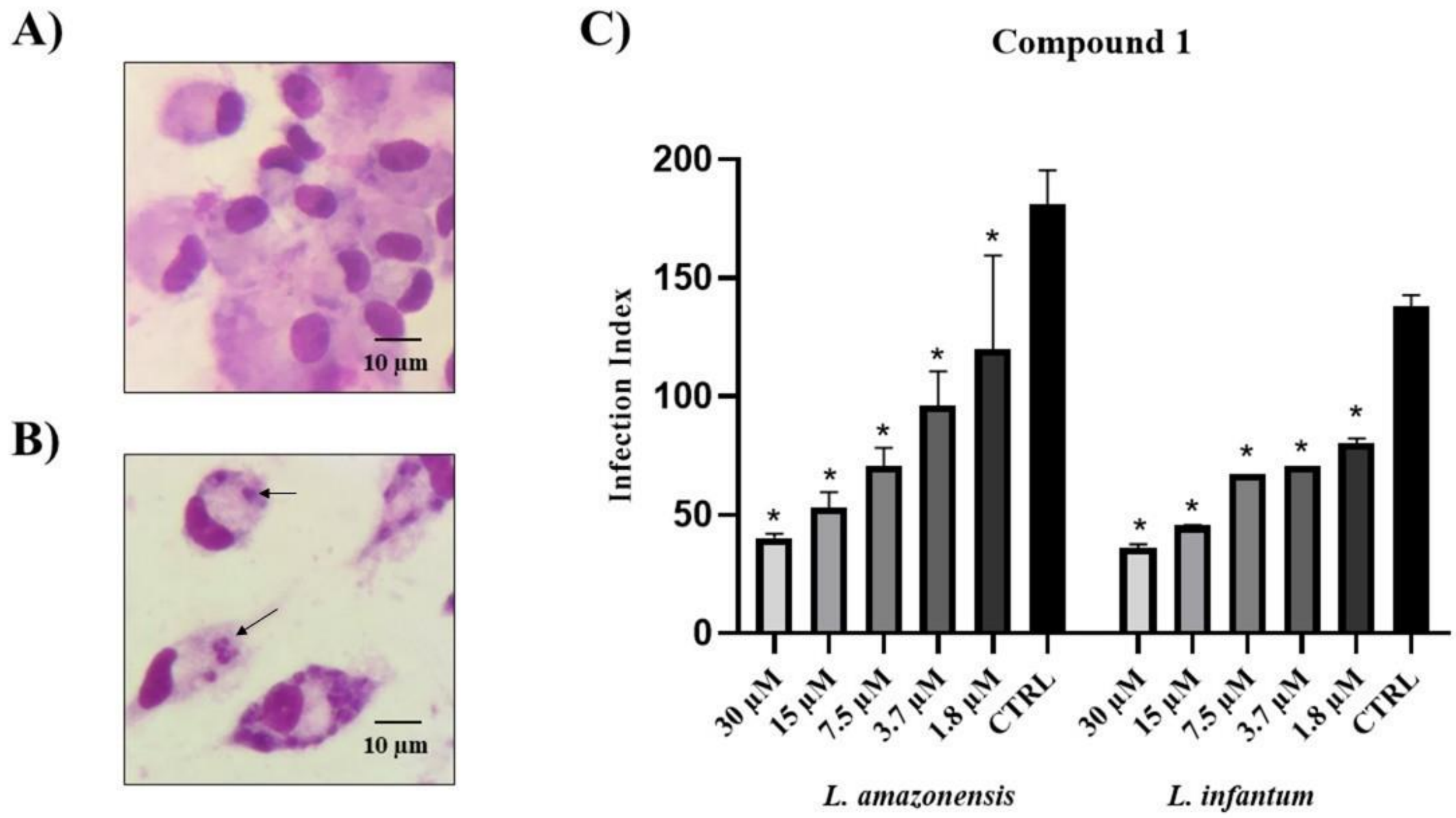

Figure S2. Representative microscopic images and infection index of *Leishmania*-infected macrophages. (A) Representative image of a non-infected macrophage. (B) Representative image of a macrophage infected with *Leishmania*, showing intracellular amastigotes (arrows). Scale bar: 10 µm. (C) Infection index of macrophages infected with *L. infantum* and *L. amazonensis* following treatment with compound **1** at different concentrations. Data are presented as the mean ± SD of two independent experiments. Statistical significance was determined by two-way ANOVA. *Statistically significant differences compared with untreated *Leishmania*-infected macrophages (CTRL) are indicated. A p-value < 0.05 was considered statistically significant.

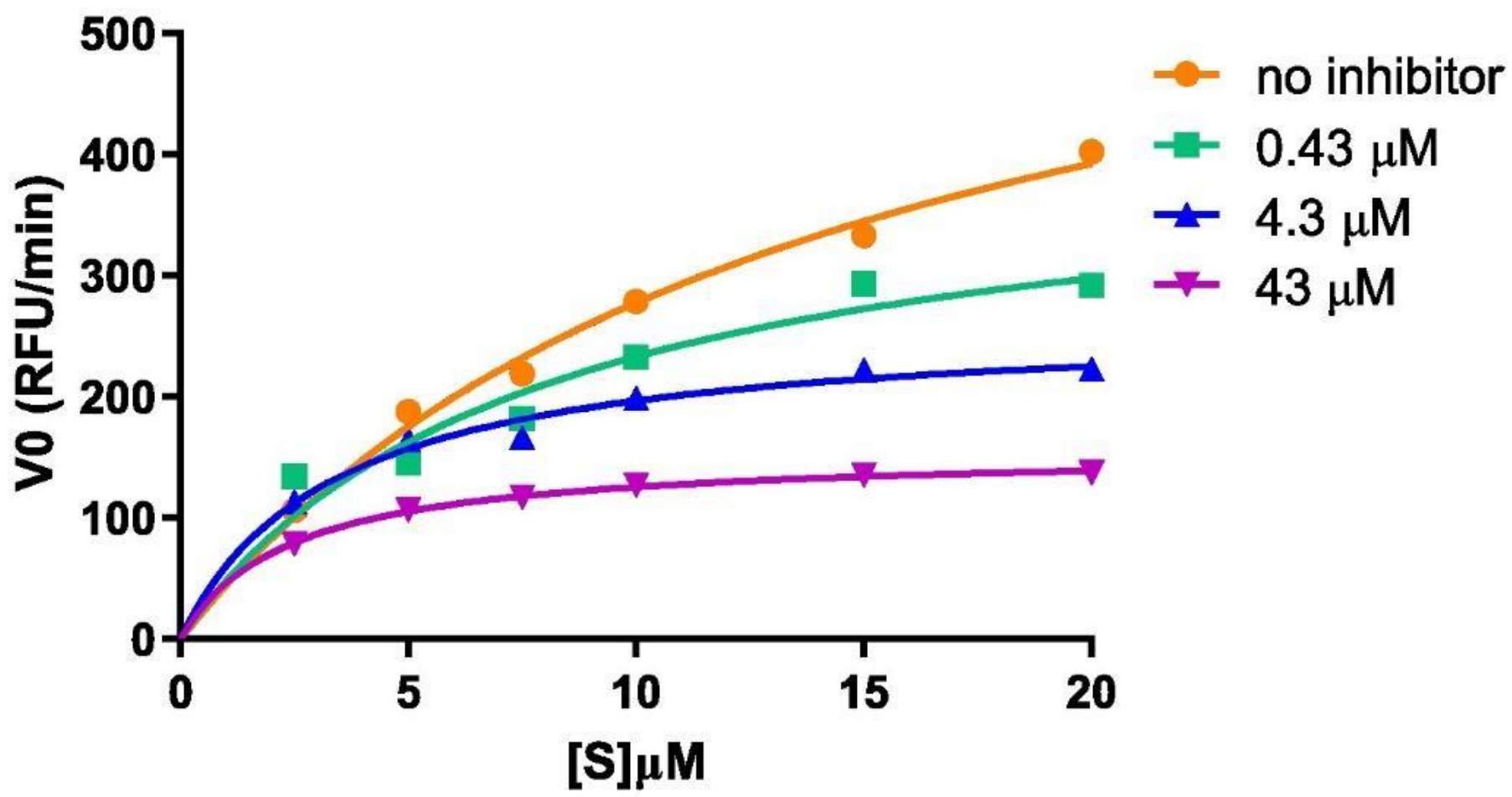

Figure S3. Michaelis-Menten kinetic profile of *Leishmania* cysteine protease B (CPB) enzyme activity. Initial reaction velocity is plotted as a function of substrate (Z-Phe-Arg-AMC) concentration [S]. The characteristic hyperbolic curve represents the enzyme saturation kinetics the presence of 0.43 μM, 4.3 μM; and 43 μM of compound **1**.

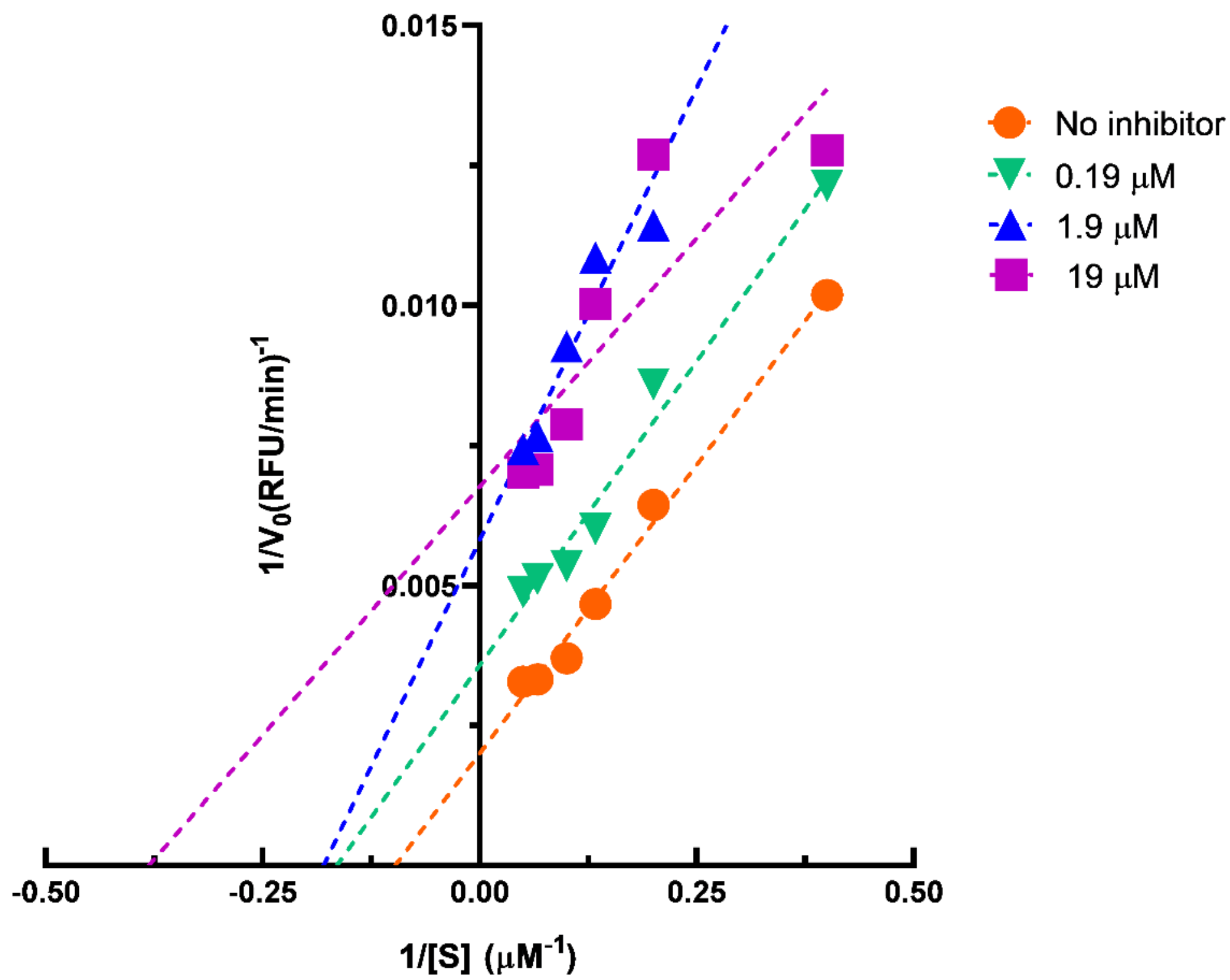

Figure S4. Double reciprocal Lineweaver-Burk plot showing the relationship between the reciprocal of the cysteine protease B enzyme activity ($1/V_0$) and the reciprocal of the substrate concentration (1/[S]). The intercept of the plot with the abscissa gives the reciprocal of the Michaelis-Menten constant (l/K_m) in the absence (*w/o*) and in the presence of 0.19 µM; 1.9 µM; and 19 µM of compound **2**.

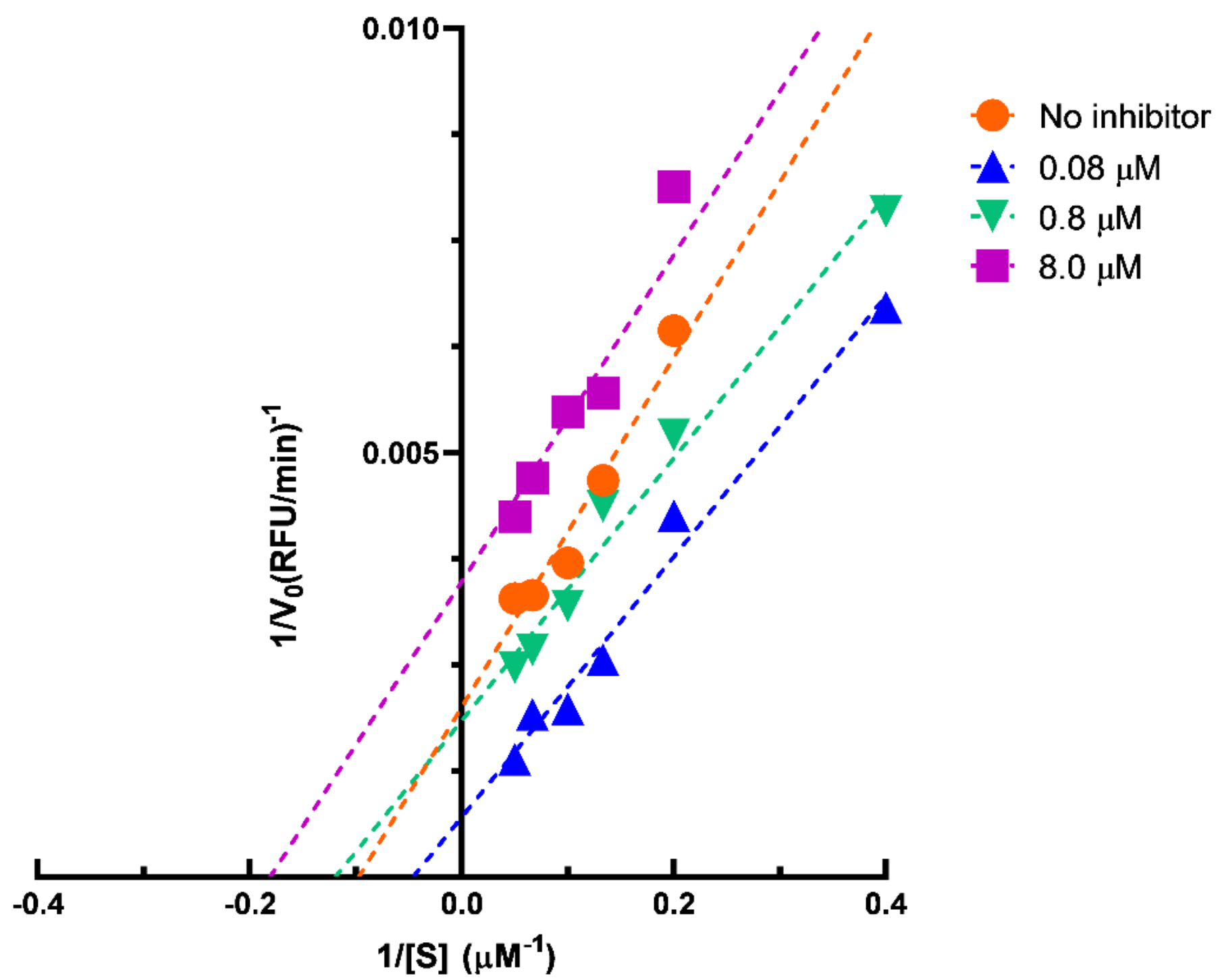

Figure S5. Double reciprocal Lineweaver-Burk plot showing the relationship between the reciprocal of the cysteine protease B enzyme activity ($1/V_0$) and the reciprocal of the substrate concentration (1/[S]). The intercept of the plot with the abscissa gives the reciprocal of the Michaelis-Menten constant (l/K_m) in the absence (*w/o*) and in the presence of 0.08 µM, 0.8 µM; and 8 µM of compound **6**.

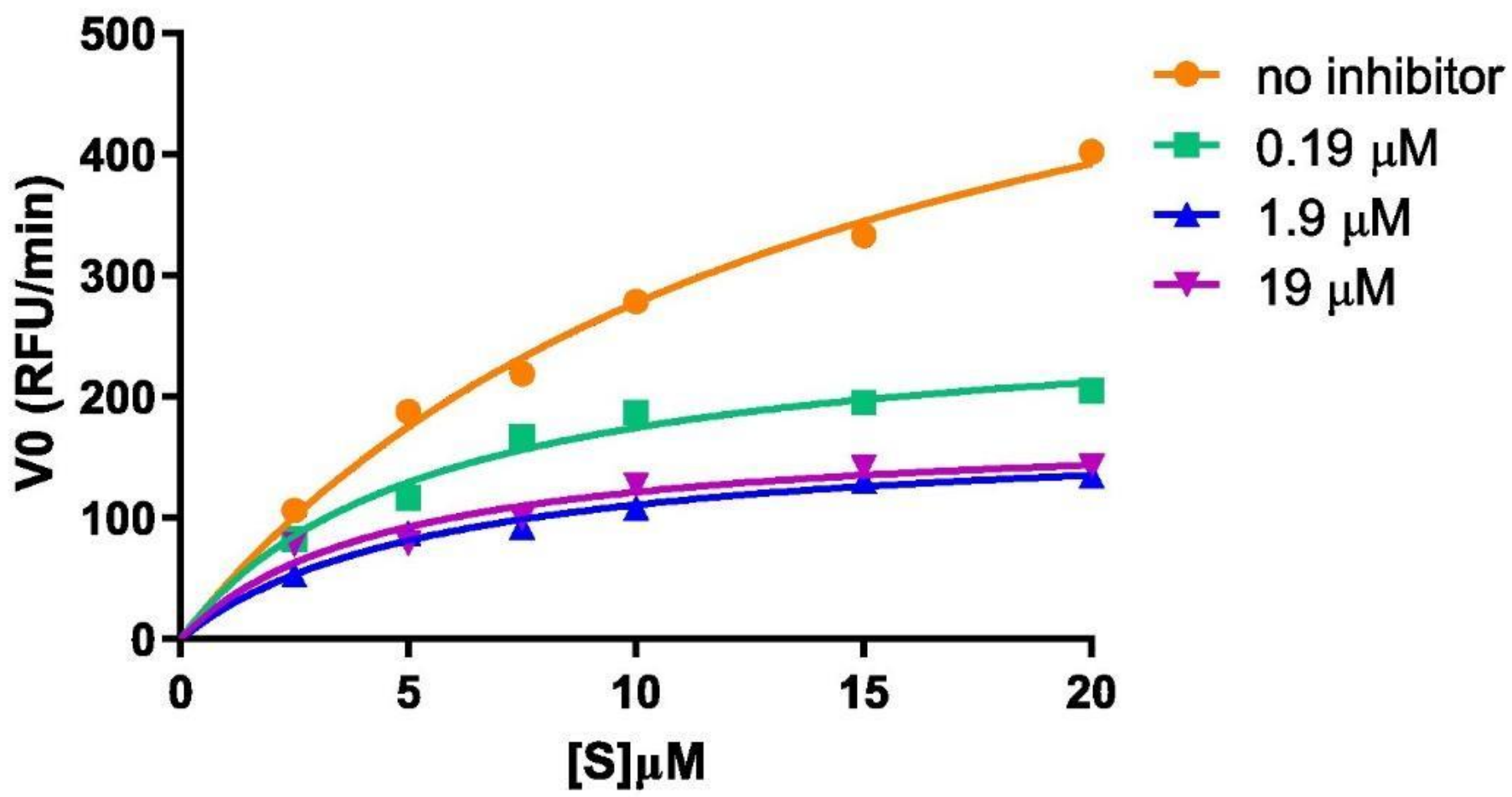

Figure S6. Michaelis-Menten kinetic profile of *Leishmania* cysteine protease B (CPB) enzyme activity. Initial reaction velocity is plotted as a function of substrate (Z-Phe-Arg-AMC) concentration [S]. The characteristic hyperbolic curve represents the enzyme saturation kinetics the presence of 0.19 µM, 1.9 µM; and 19 µM of compound **2**.

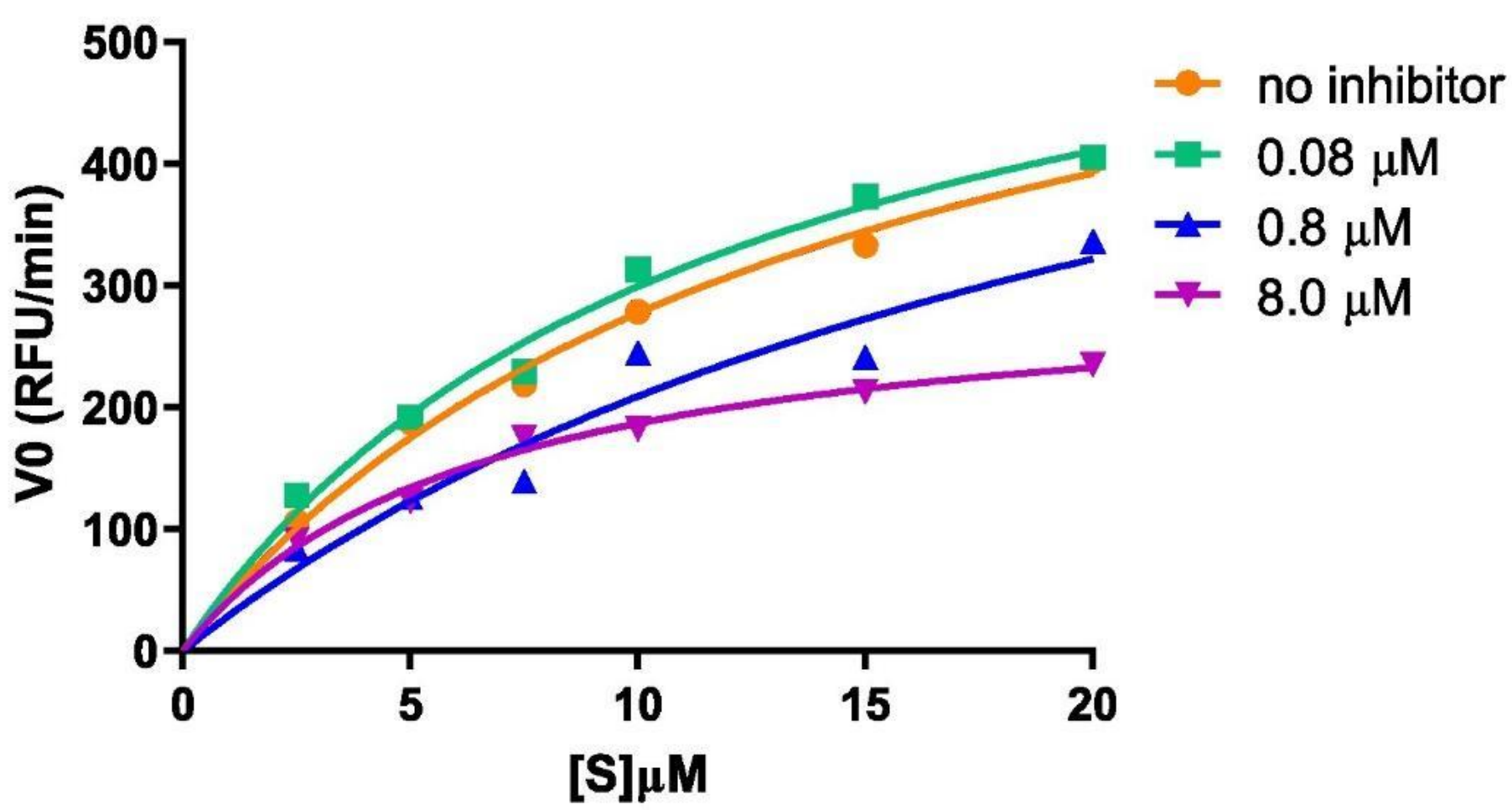

Figure S7. Michaelis-Menten kinetic profile of *Leishmania* cysteine protease B (CPB) enzyme activity. Initial reaction velocity is plotted as a function of substrate (Z-Phe-Arg-AMC) concentration [S]. The characteristic hyperbolic curve represents the enzyme saturation kinetics the presence of 0.08 µM, 0.8 µM; and 8 µM of compound **6**.

Determination of toxicity in an alternative model of *Galleria mellonella*

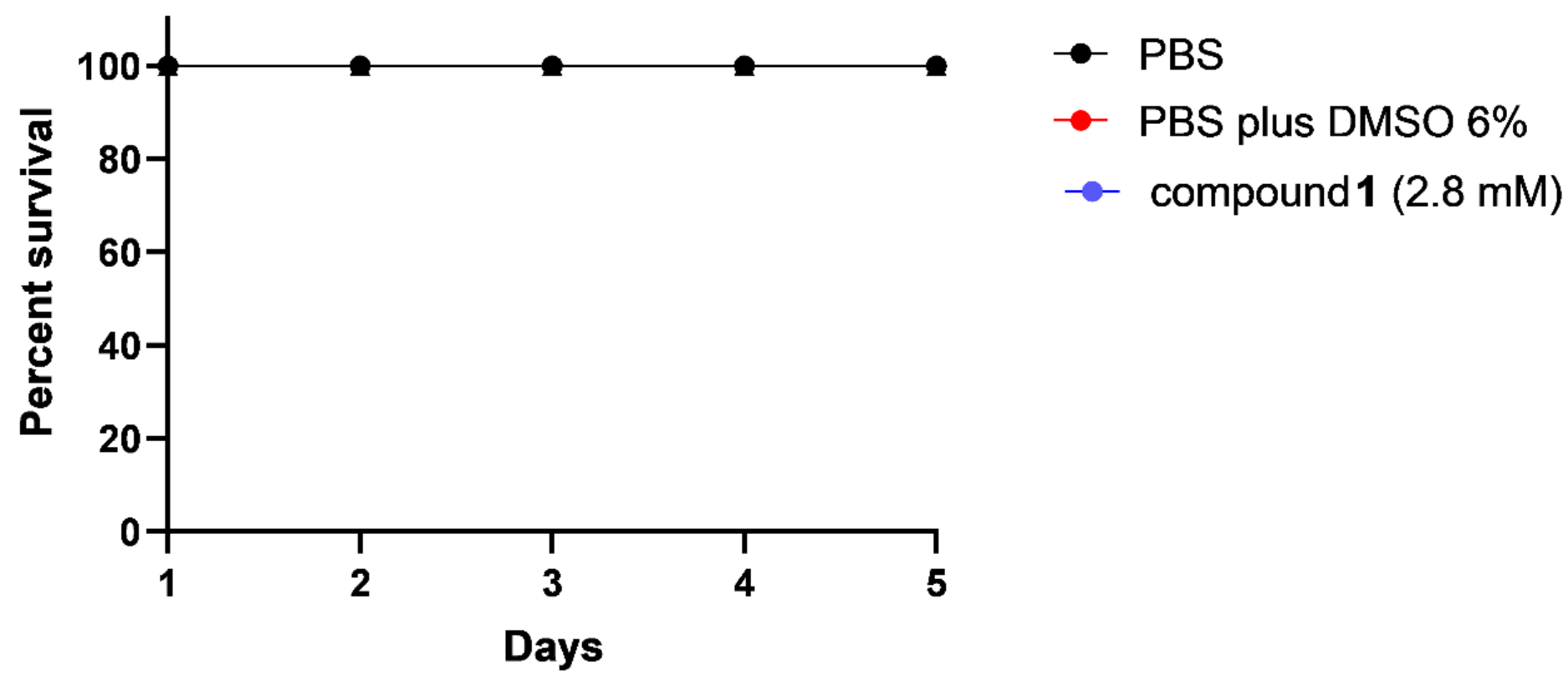

Figure S8. Percentage survival of *Galleria mellonella* larvae following treatment with different therapies over a 5-day period. Compound 1 was administered at 2.8 mM, solubilized in 6% DMSO, in a final volume of 20 µL per larva. Data are presented as the mean ± SD of three independent experiments, with n = 10 larvae per treatment group in each experiment. No statistically significant differences were observed among groups ($p > 0.05$).

Evaluation of *in vivo* efficacy and safety in BALB/c mice infected with *L. infantum*

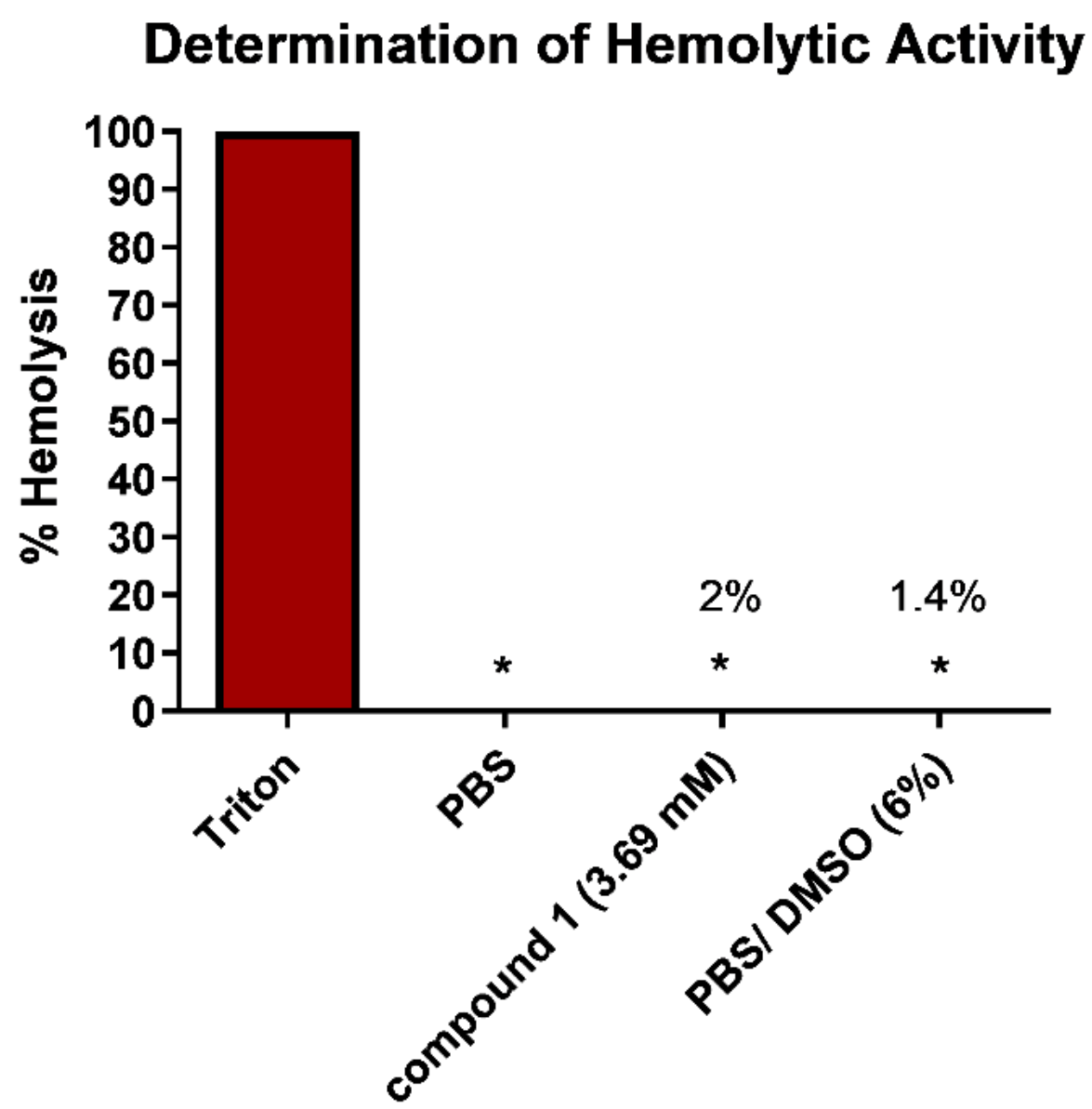

Figure S9. Hemolytic activity in human blood after exposure to compound 1 at 3.69 mM, corresponding to ten times the concentration used in the *in vivo* evaluation (0.77 mg/kg/day; equivalent to 0.369 mM). Data are presented as the mean ± SD of two independent experiments. *Statistically significant differences compared with the positive control (Triton) were observed for compound **1**, PBS, and PBS containing 6% DMSO ($p < 0.05$).

EXPERIMENTAL SECTION

General Procedure for the Synthesis of Dipeptidyl Nitrile Derivatives

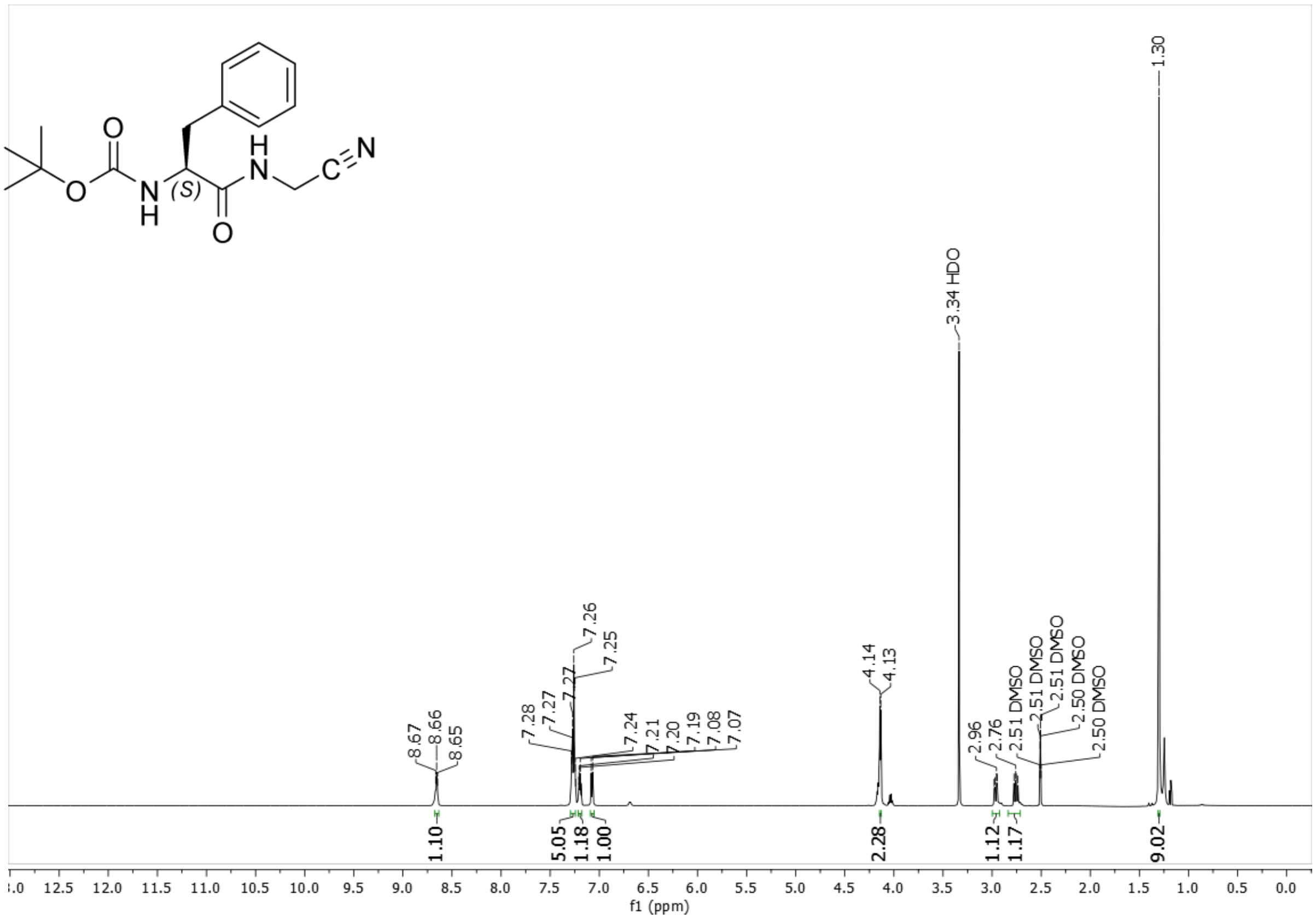

Figure S10. ^{1}H-NMR Spectrum of compound **1** (600 MHz DMSO-*d*6).

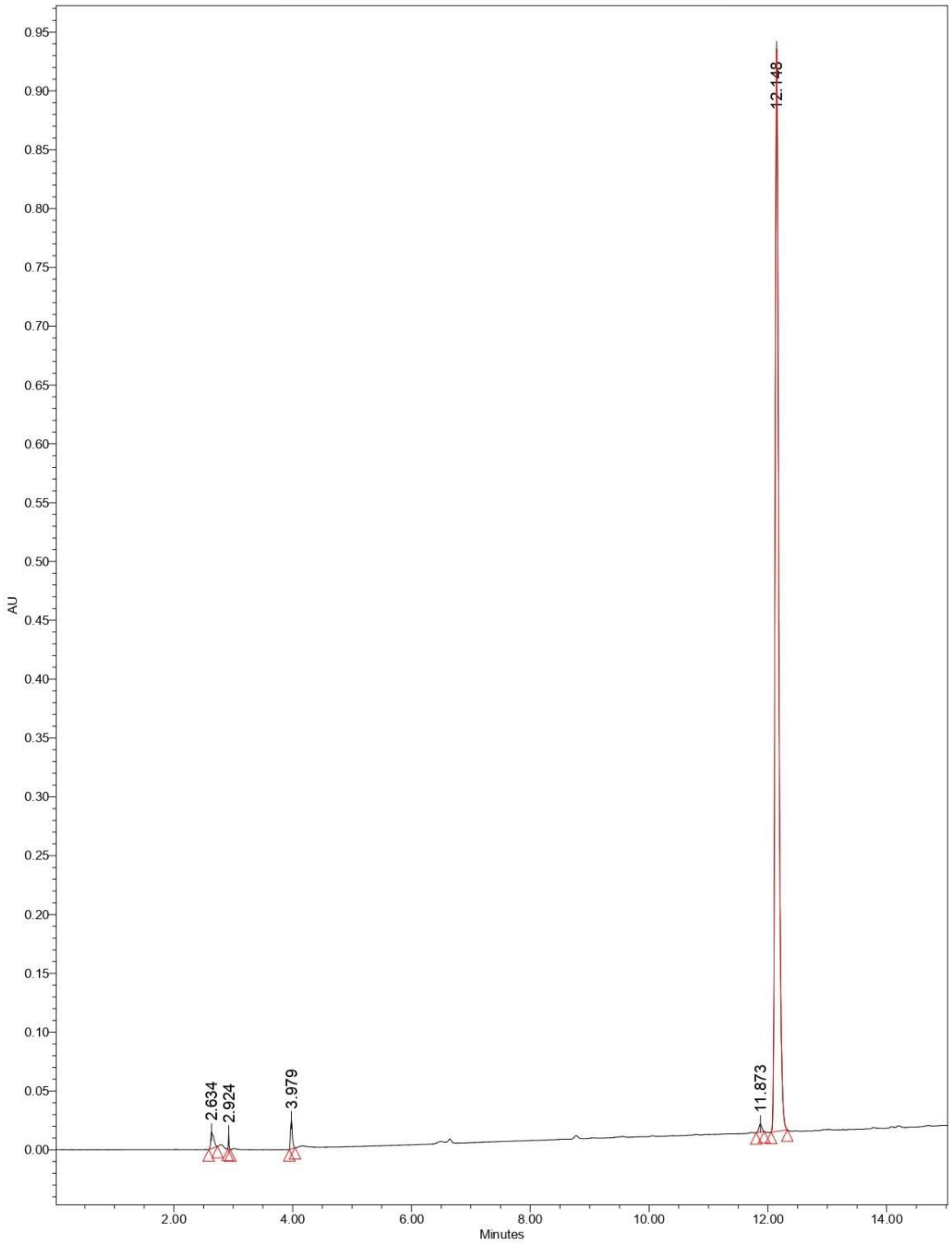

	Name	Retention Time (min)	Purity1 Angle	Purity1 Threshold	PDA/FLR Match1 Spect. Name	PDA/FLR Match1 Angle	PDA/FLR Match1 Threshold	PDA/FLR Match1 Lib. Name	Area (µV*sec)	% Area	Height (µV)
1		2.634							49381	1.22	14030
2		2.924							10742	0.27	13460
3		3.979							52142	1.29	24851
4		11.873							23408	0.58	6688
5		12.148							3911099	96.65	920419

Figure S11. HPLC–PDA chromatogram of compound **1** for purity analysis (R.t = 12.11 min).

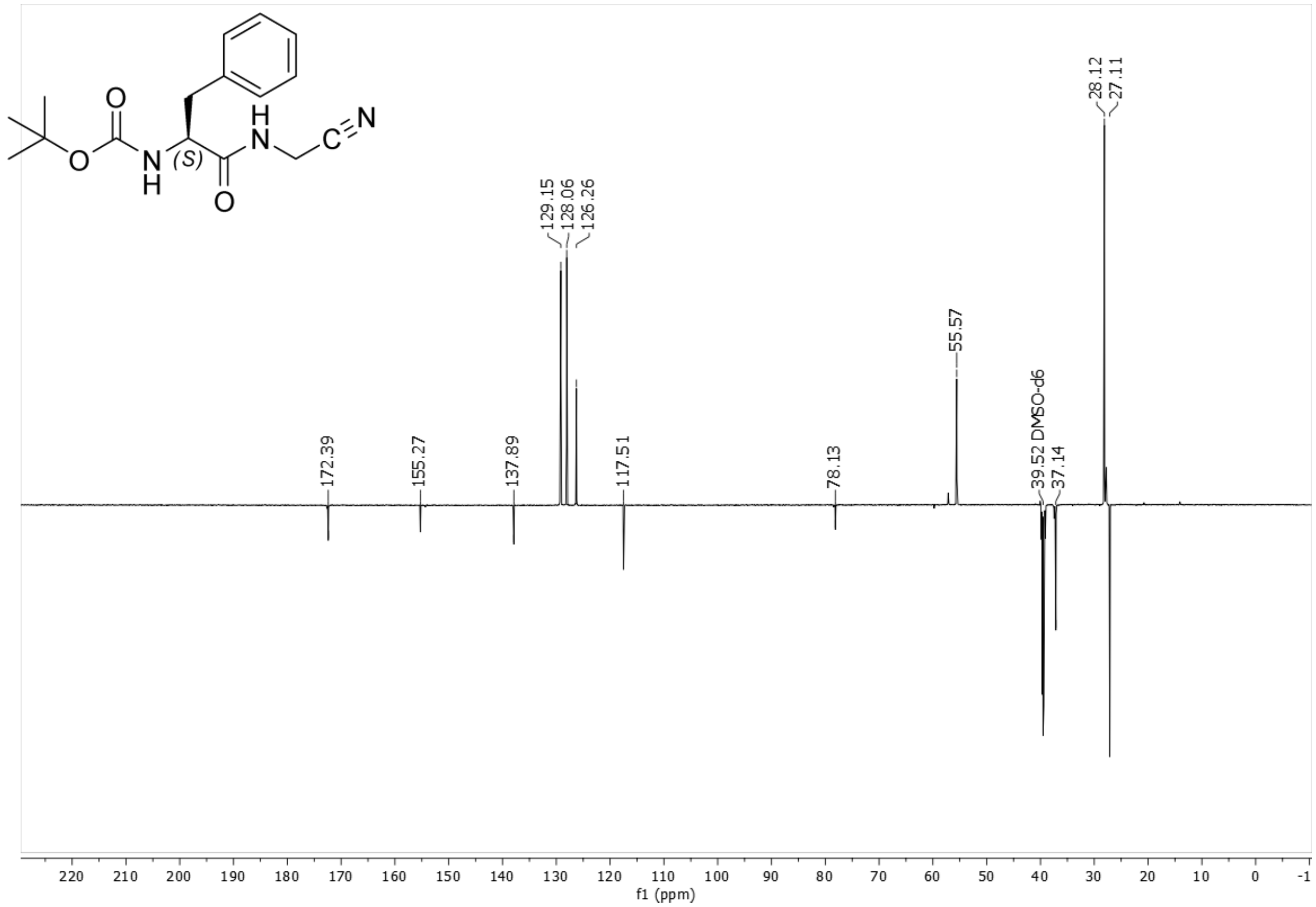

Figure S12. DEPTQ-NMR Spectrum of compound **1** (150 MHz DMSO-*d*6).

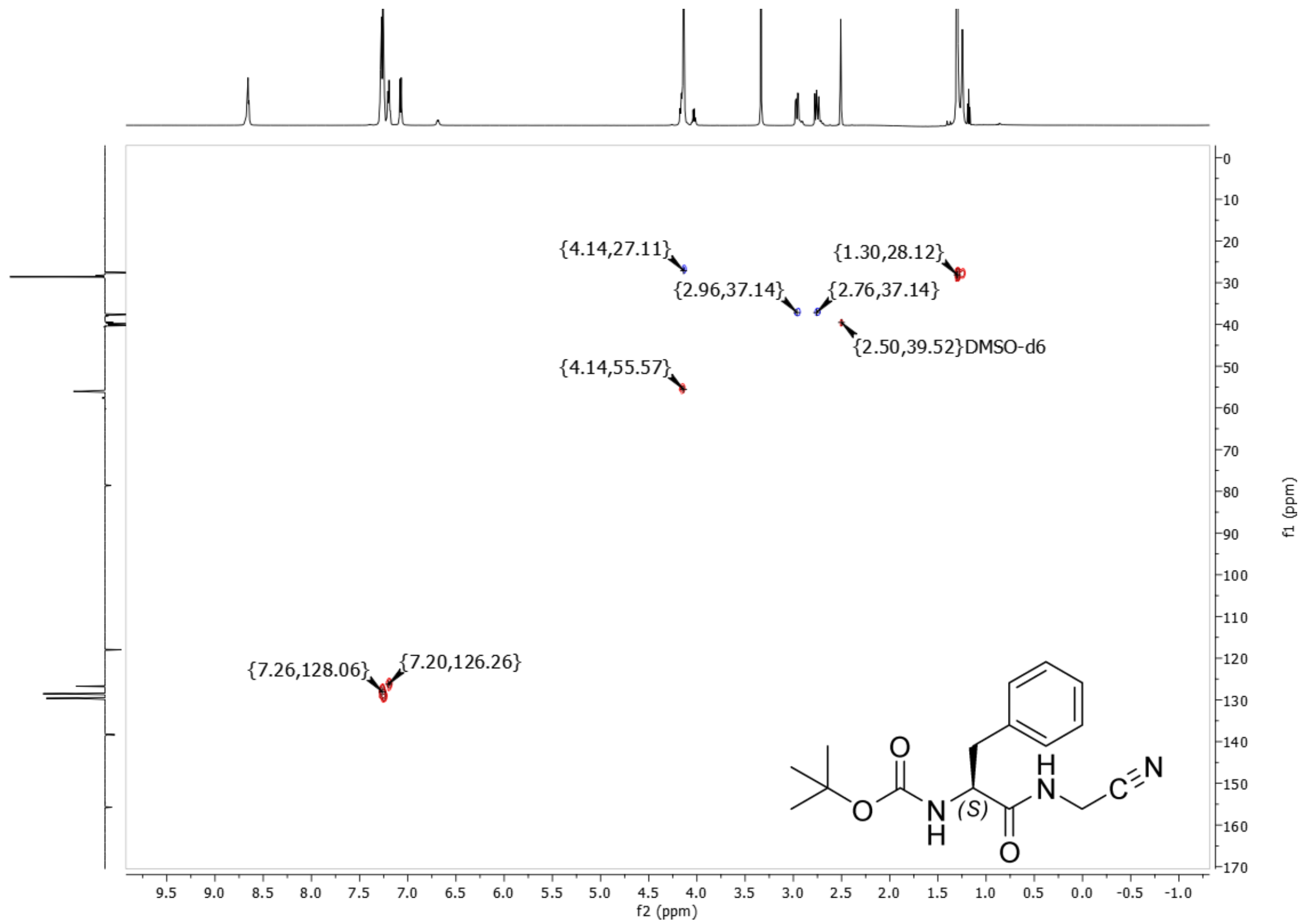

Figure S13. HSQC-NMR Spectrum of compound **1** (150 MHz DMSO-*d*6).

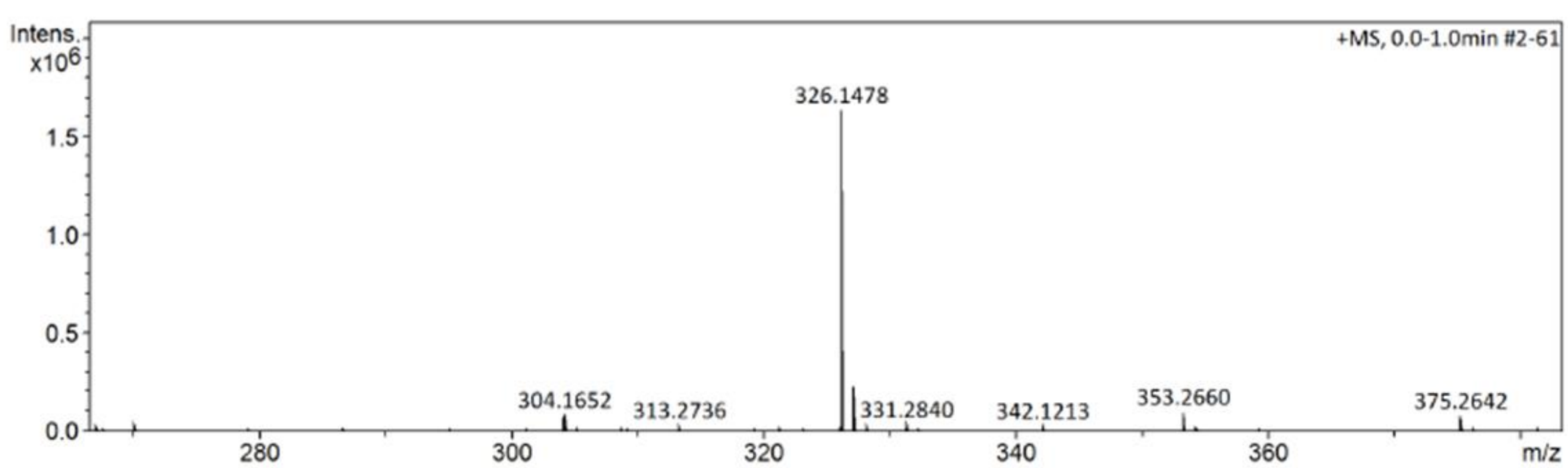

Figure S14. HRMS Spectrum of compound **1** (ESI^{+}).

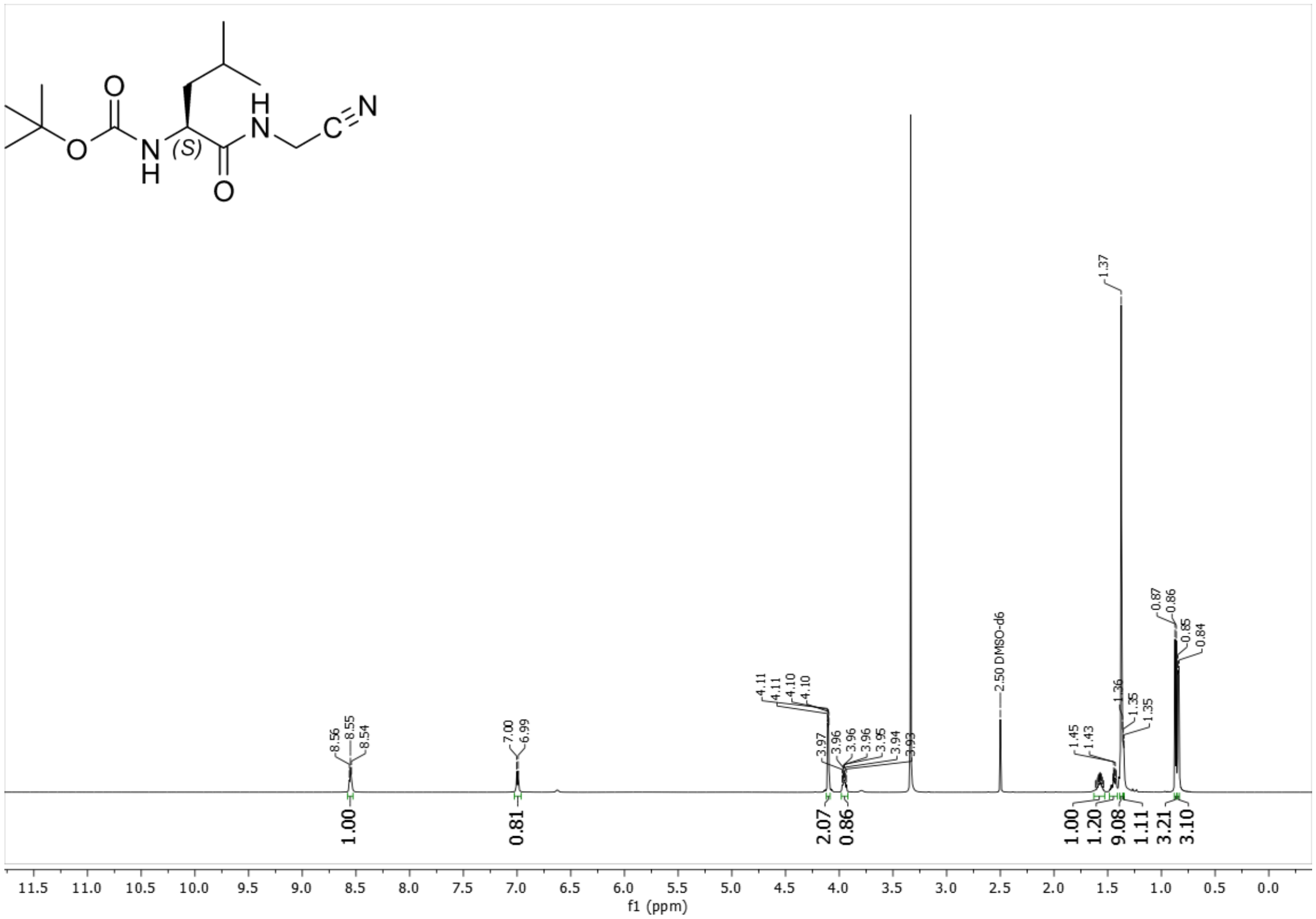

Figure S15. ^{1}H-NMR Spectrum of compound **2** (600 MHz DMSO-*d*6).

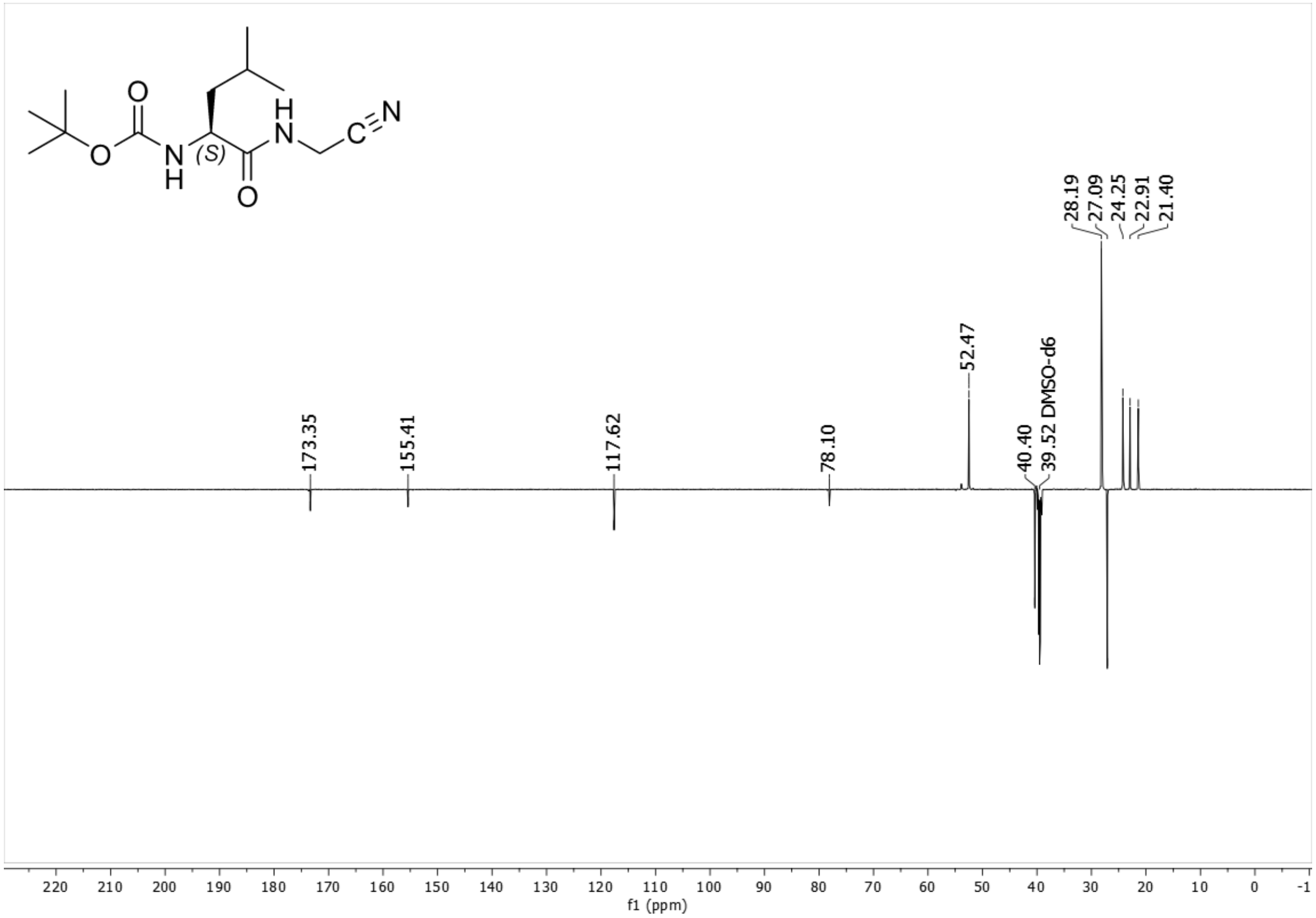

Figure S16. DEPTQ-NMR Spectrum of compound **2** (150 MHz DMSO-*d*6).

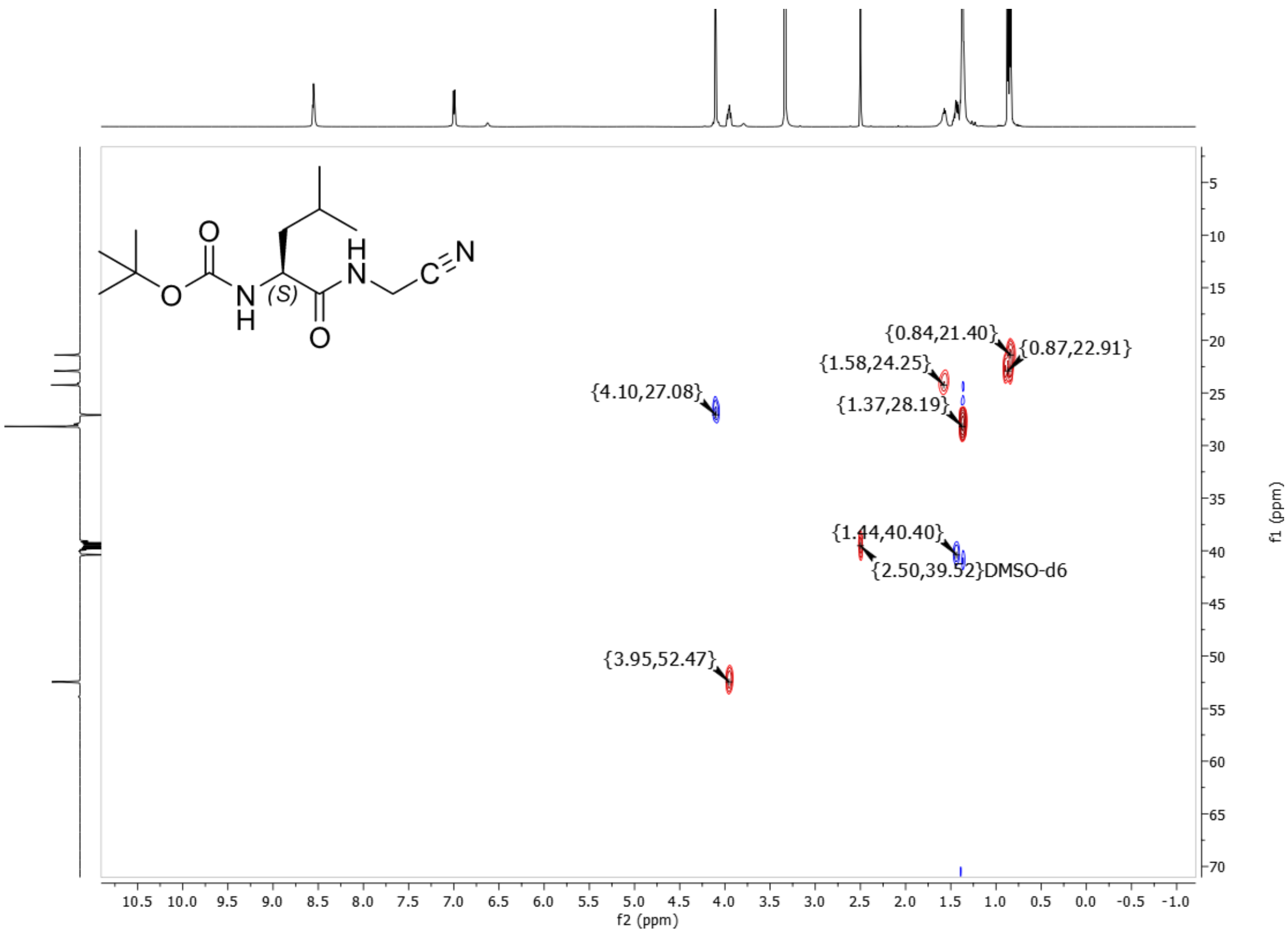

Figure S17. HSQC-NMR Spectrum of compound **2** (150 MHz DMSO-*d*6).

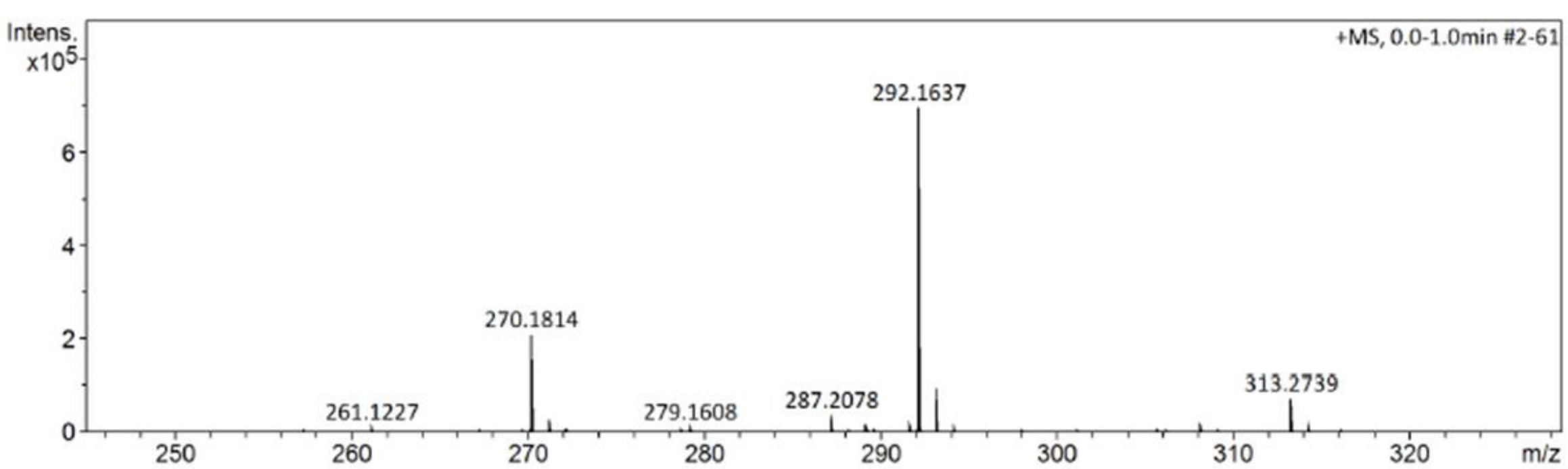

Figure S18. HRMS Spectrum of compound **2** (ESI$^+$).

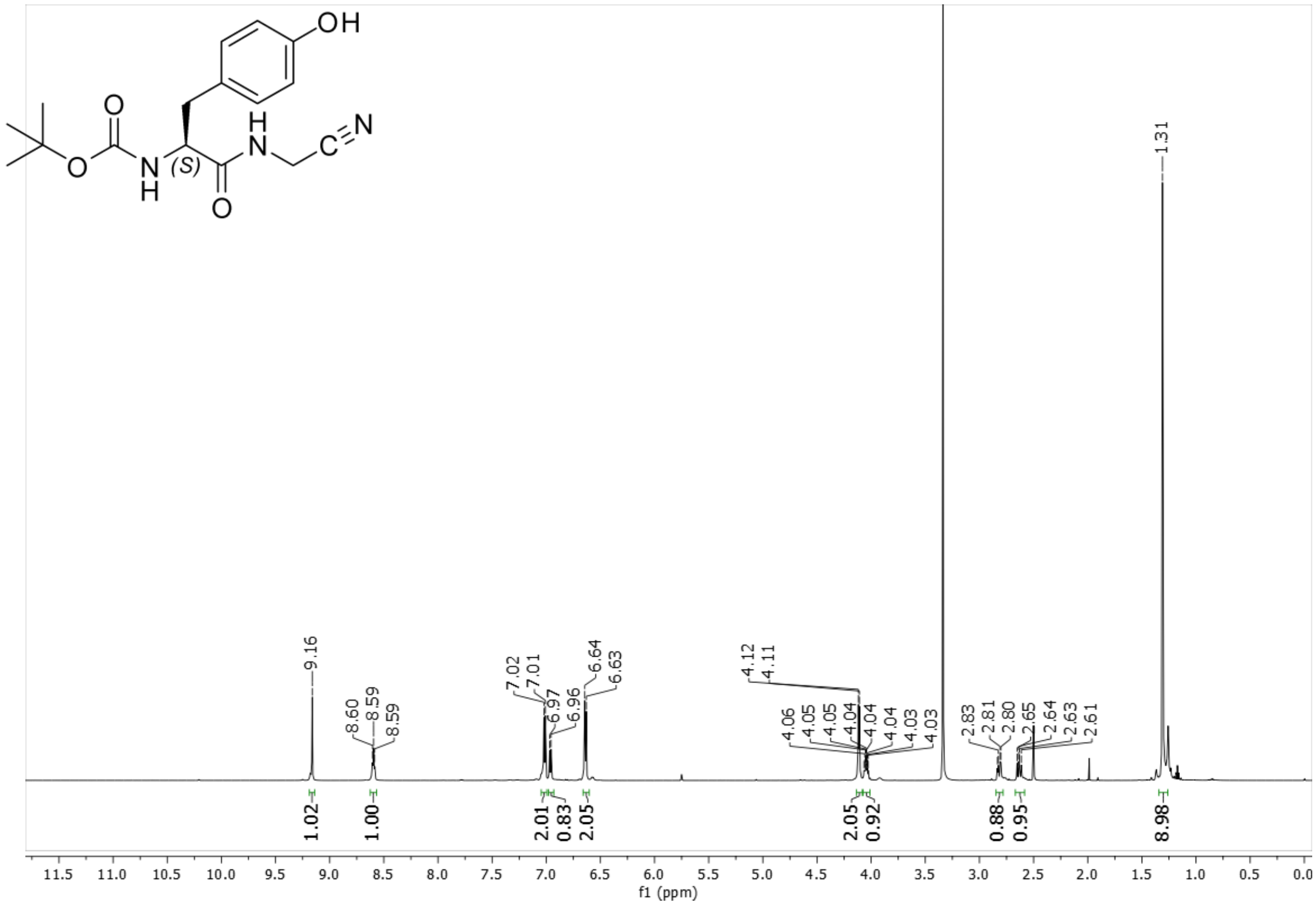

Figure S19. ^{1}H-NMR Spectrum of compound **3** (600 MHz DMSO-*d*6).

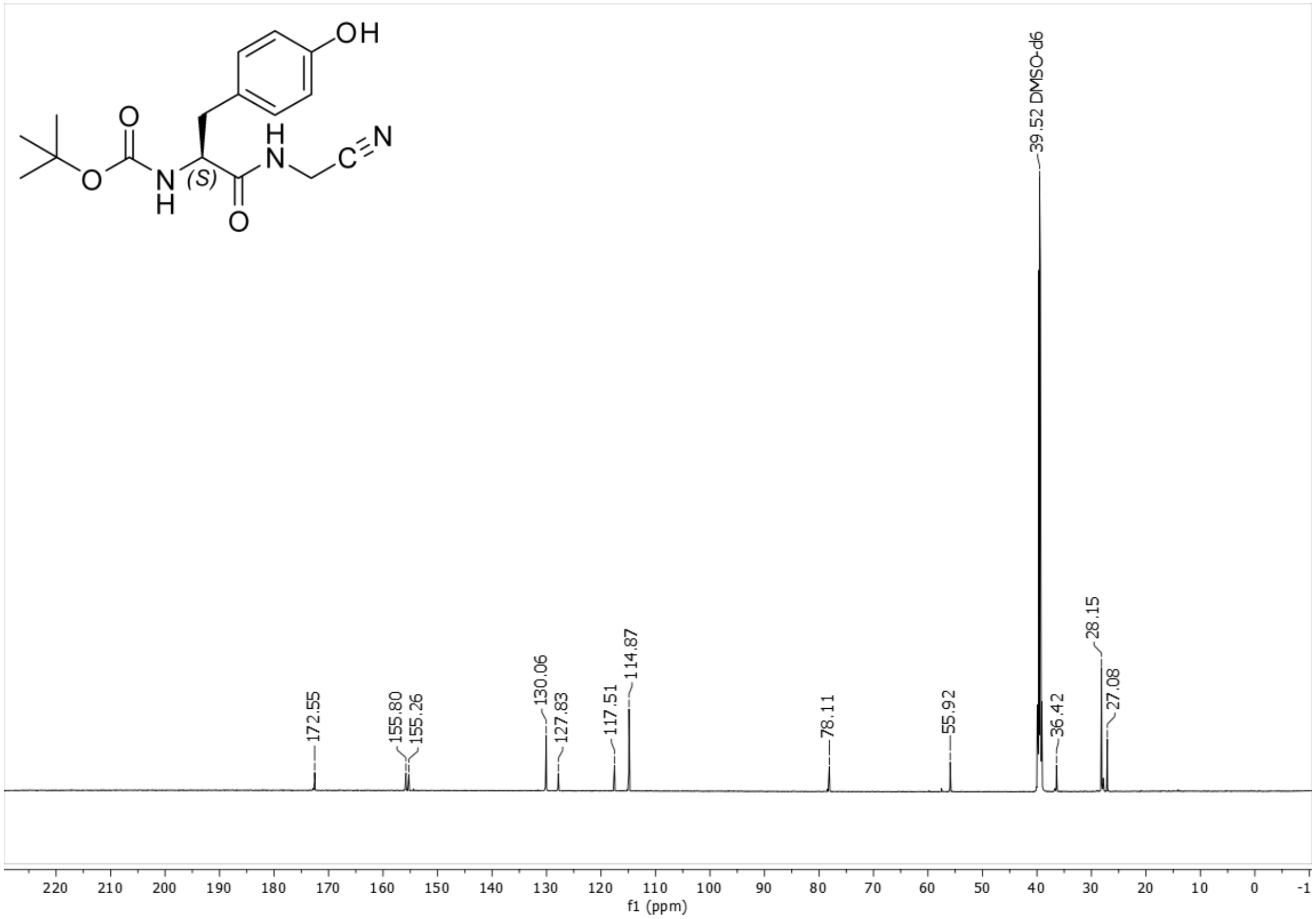

Figure S20. DEPTQ-NMR Spectrum of compound **3** (150 MHz DMSO-*d*6).

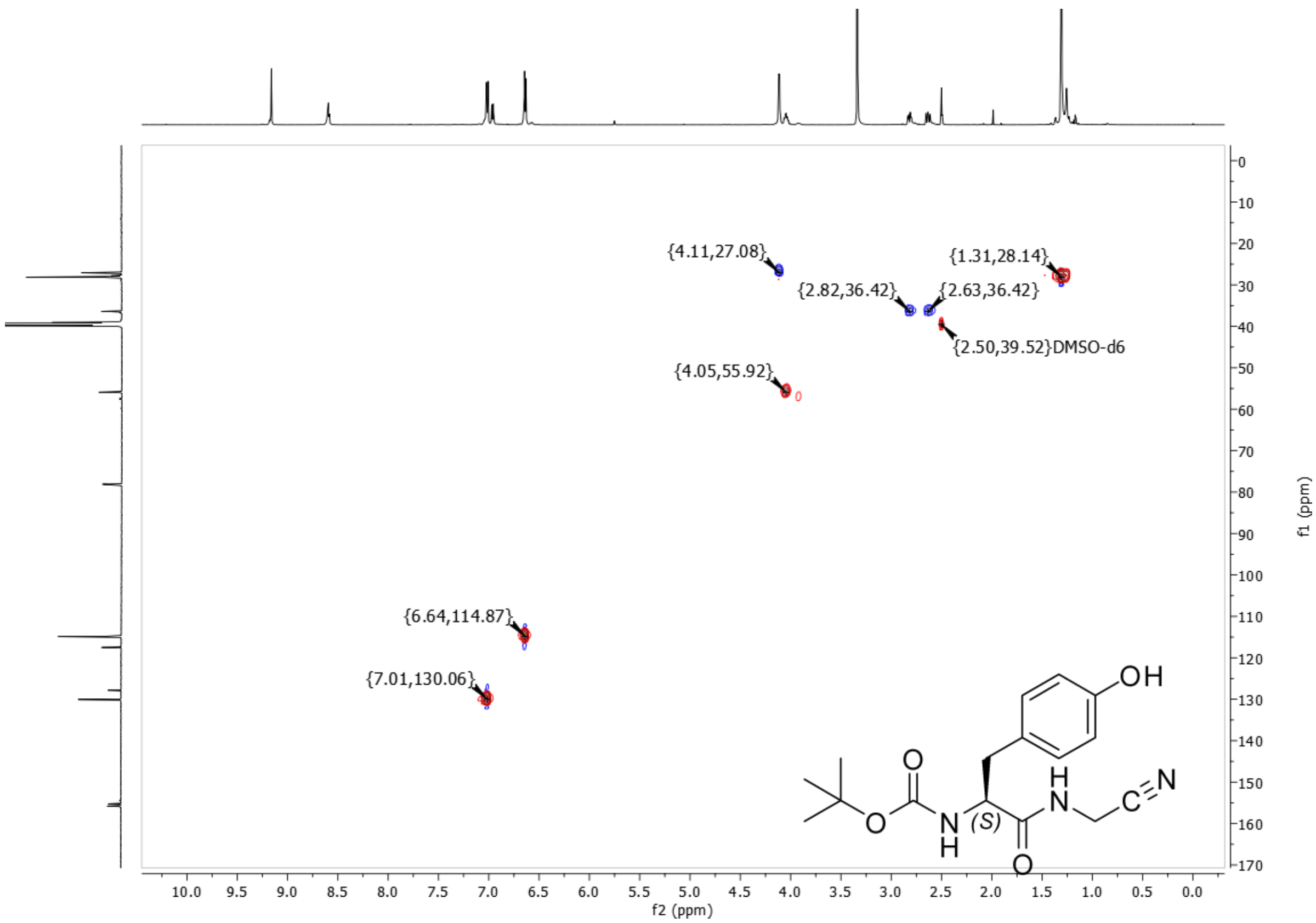

Figure S21. HSQC-NMR Spectrum of compound **3** (150 MHz DMSO-*d*6).

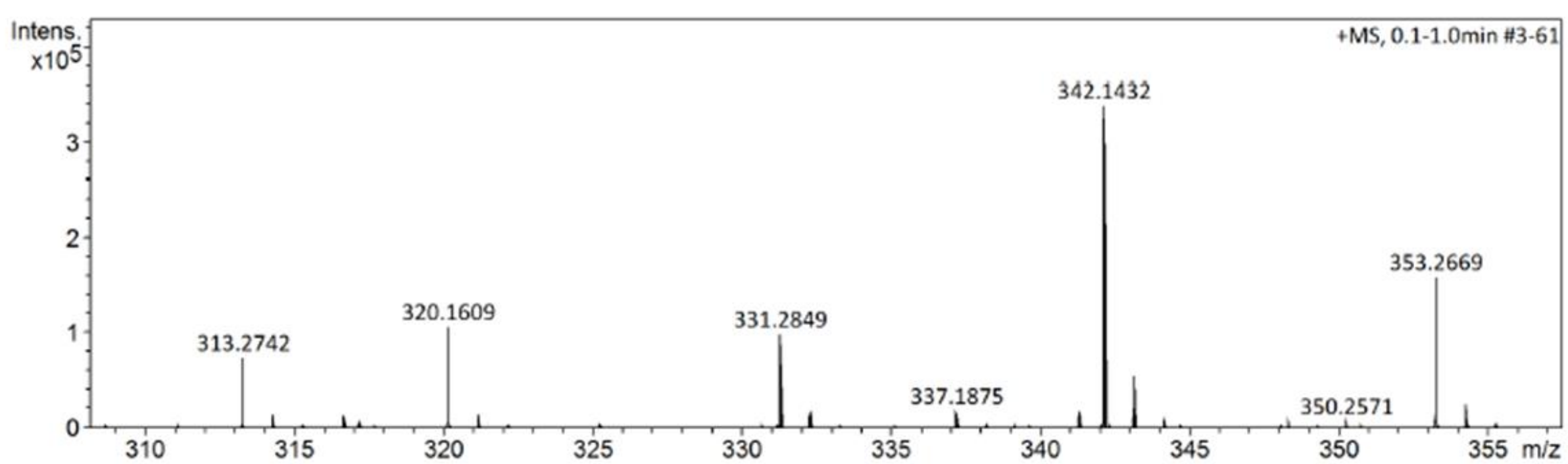

Figure S22. HRMS Spectrum of compound **3** (ESI^{+}).

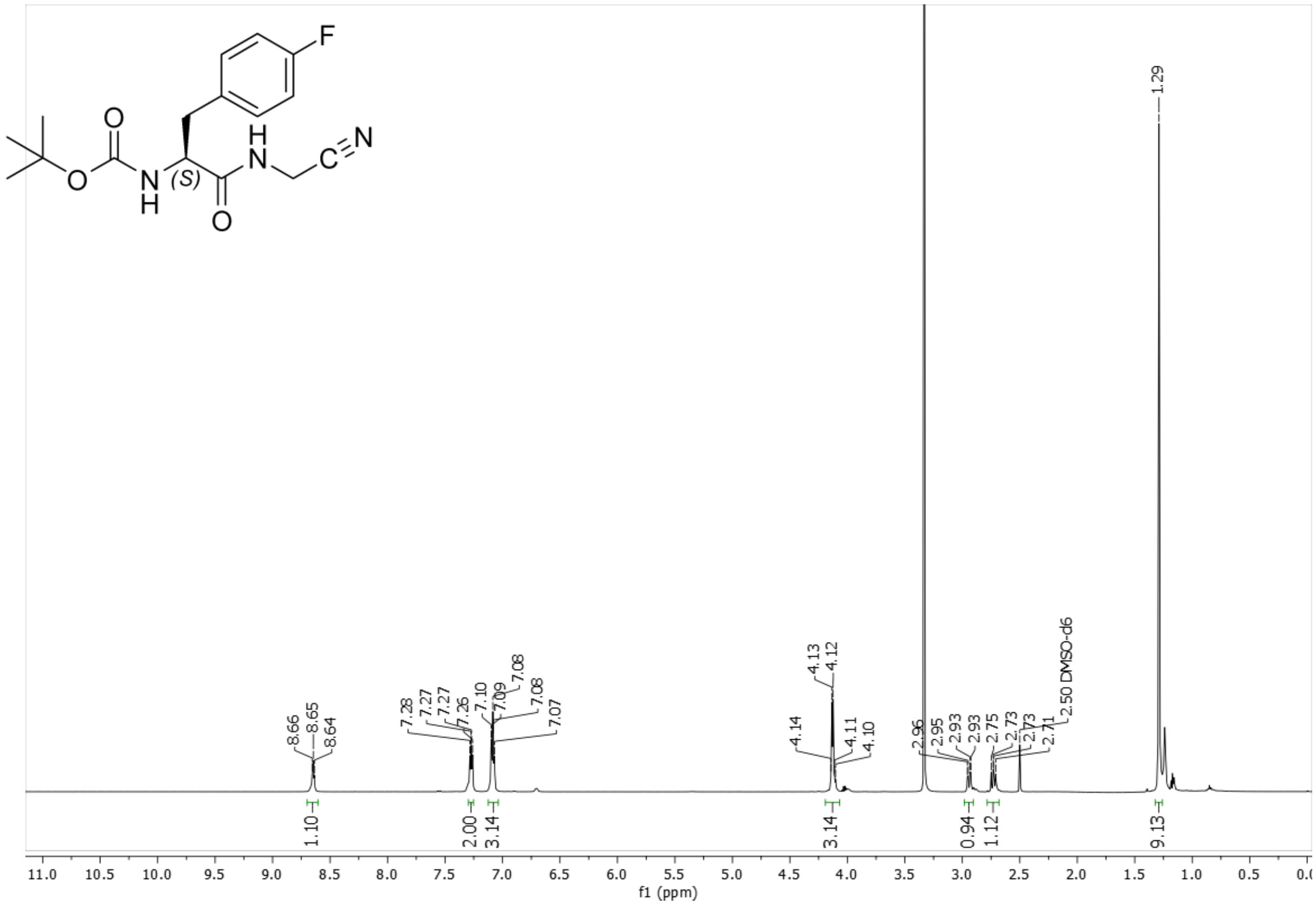

Figure S23. ^{1}H-NMR Spectrum of compound **4** (600 MHz DMSO-*d*6).

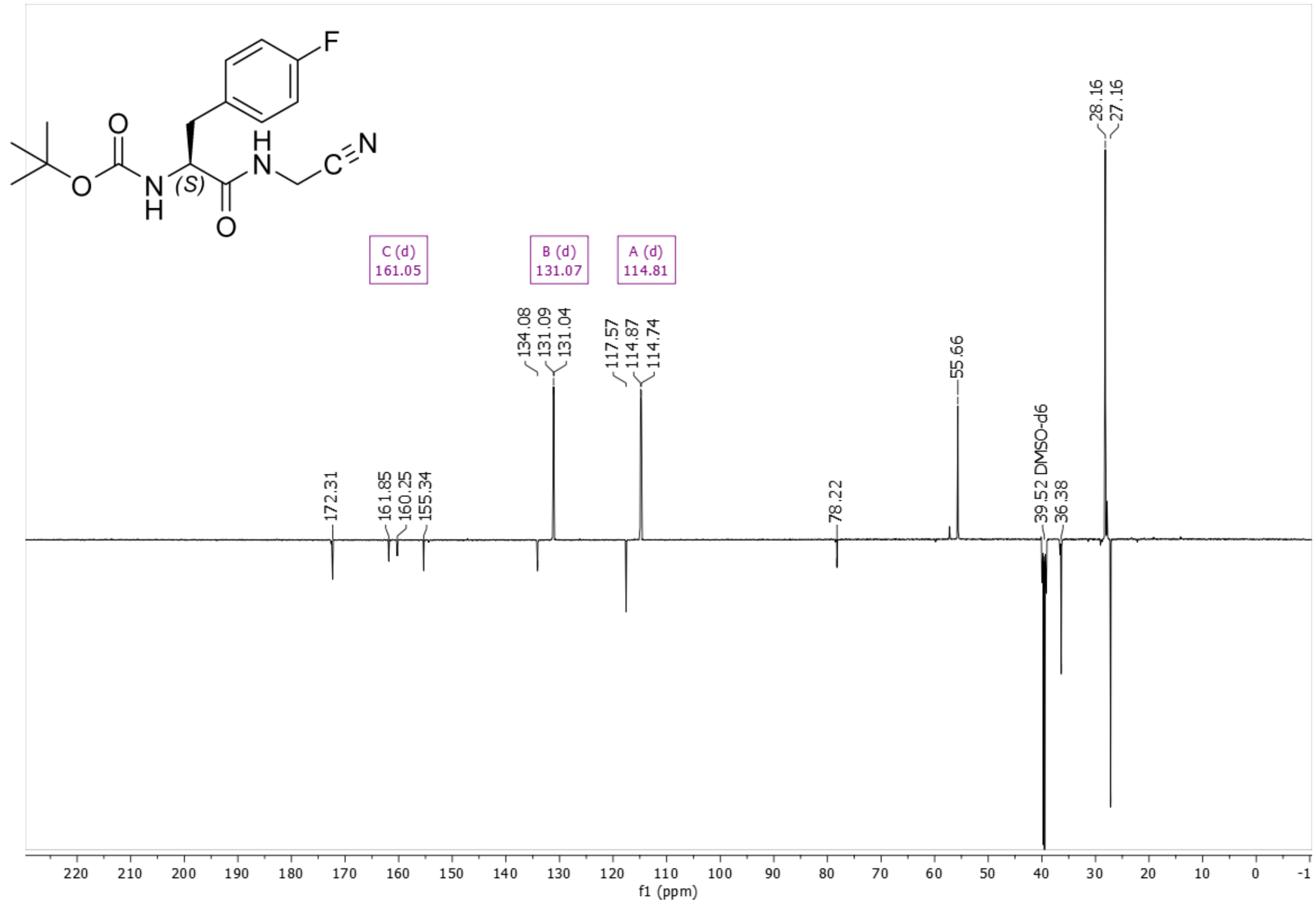

Figure 24. DEPTQ-NMR Spectrum of compound **4** (150 MHz DMSO-*d*6).

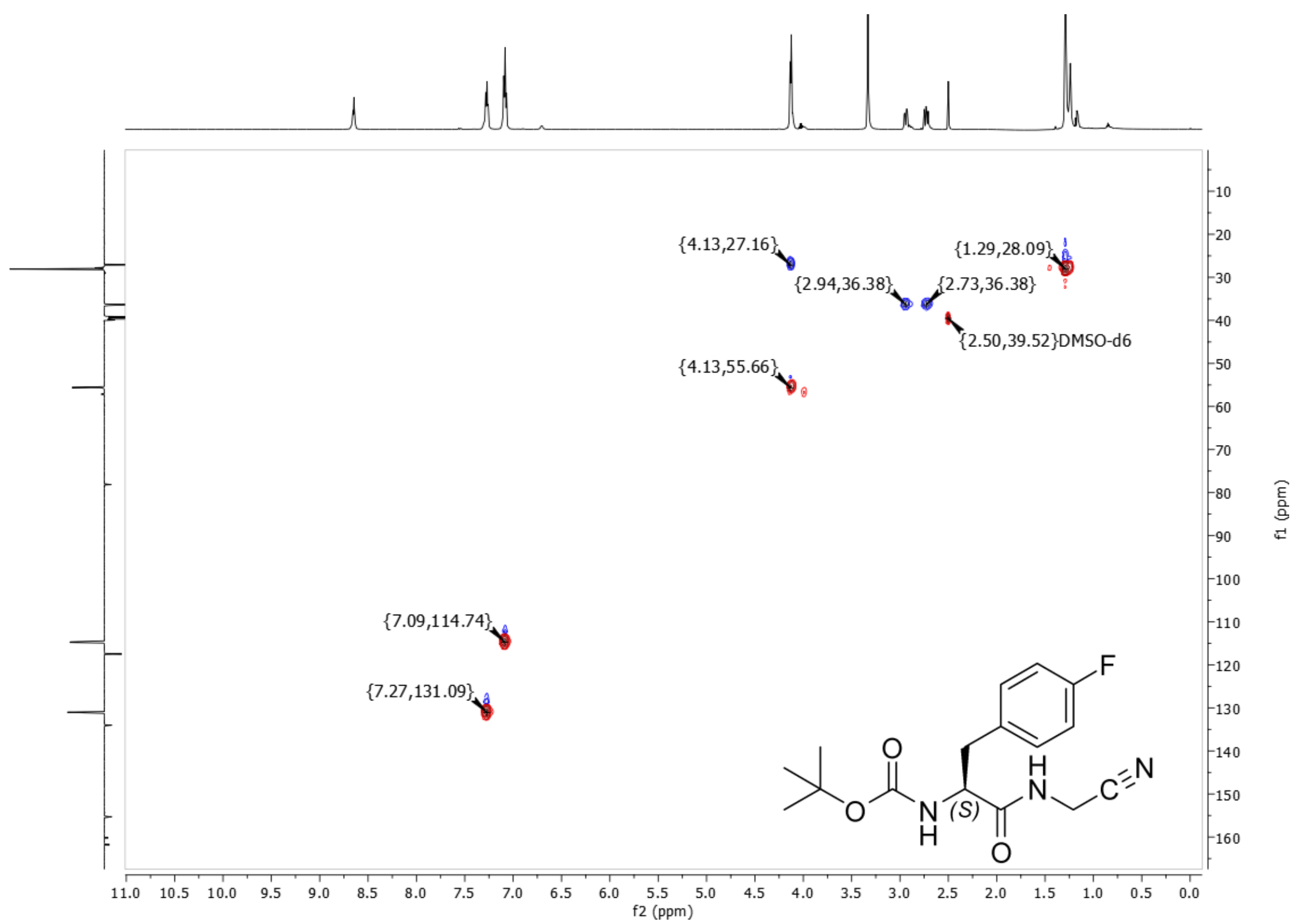

Figure S25. HSQC-NMR Spectrum of compound **4** (150 MHz DMSO-*d*6).

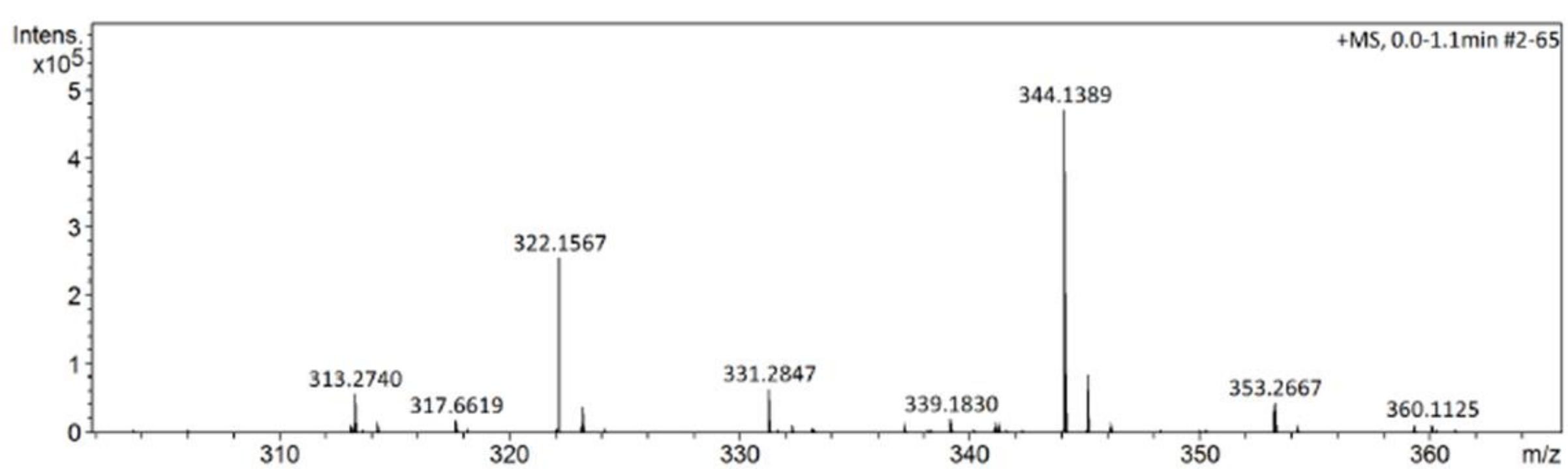

Figure S26. HRMS Spectrum of compound **4** (ESI$^+$).

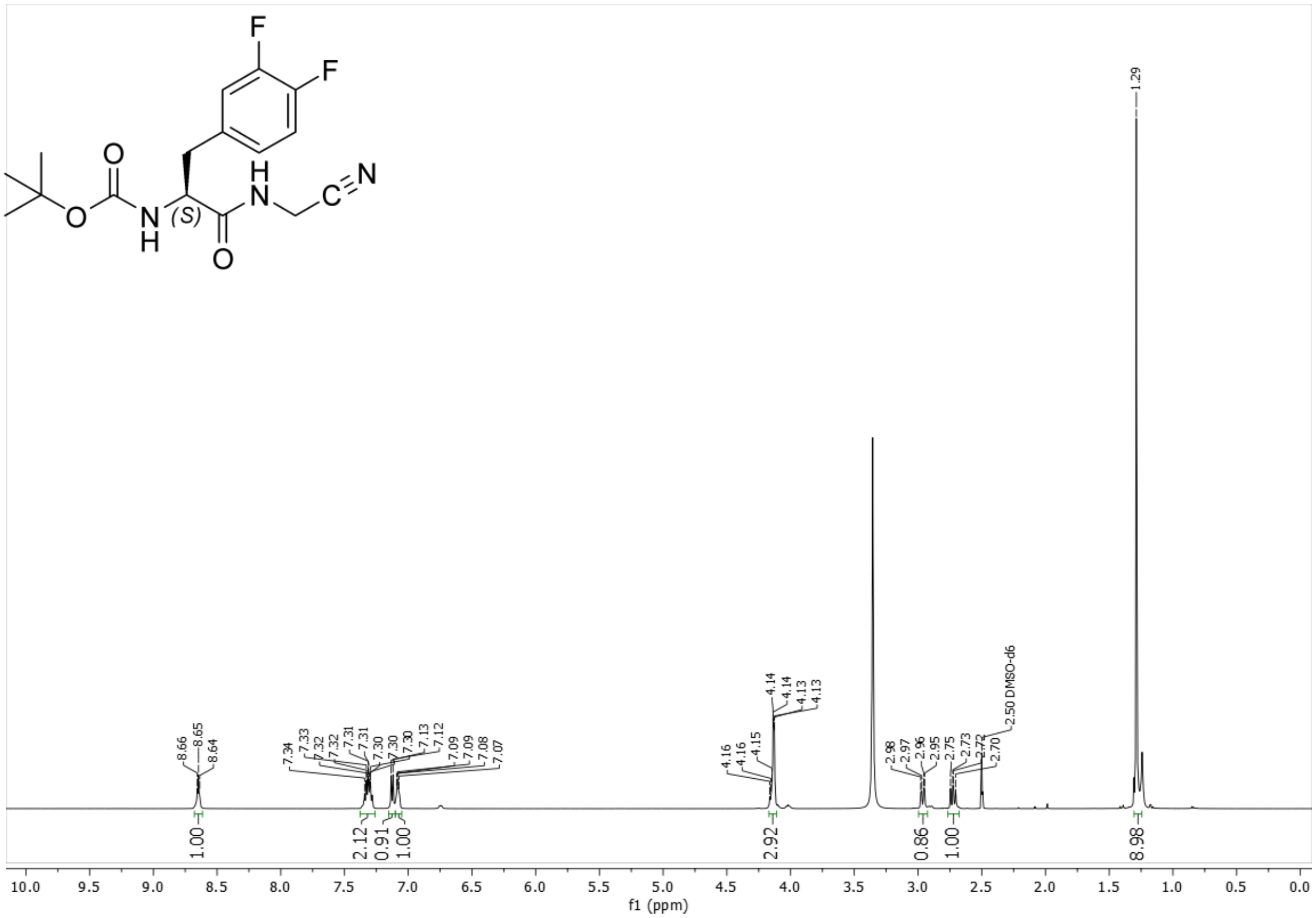

Figure S27. ^{1}H-NMR Spectrum of compound **5** (600 MHz DMSO-*d*6).

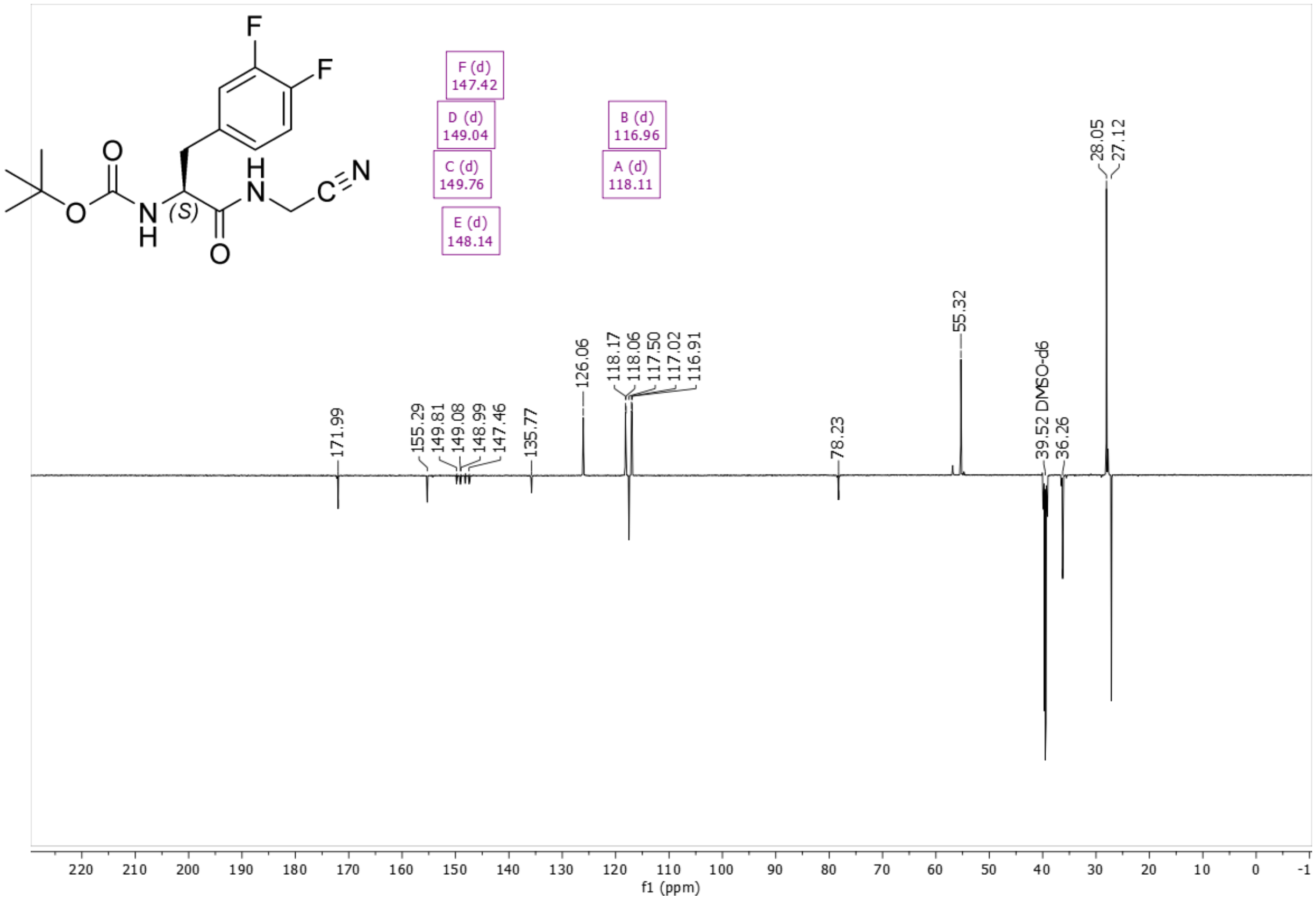

Figure S28. DEPTQ-NMR Spectrum of compound **5** (150 MHz DMSO-*d*6).

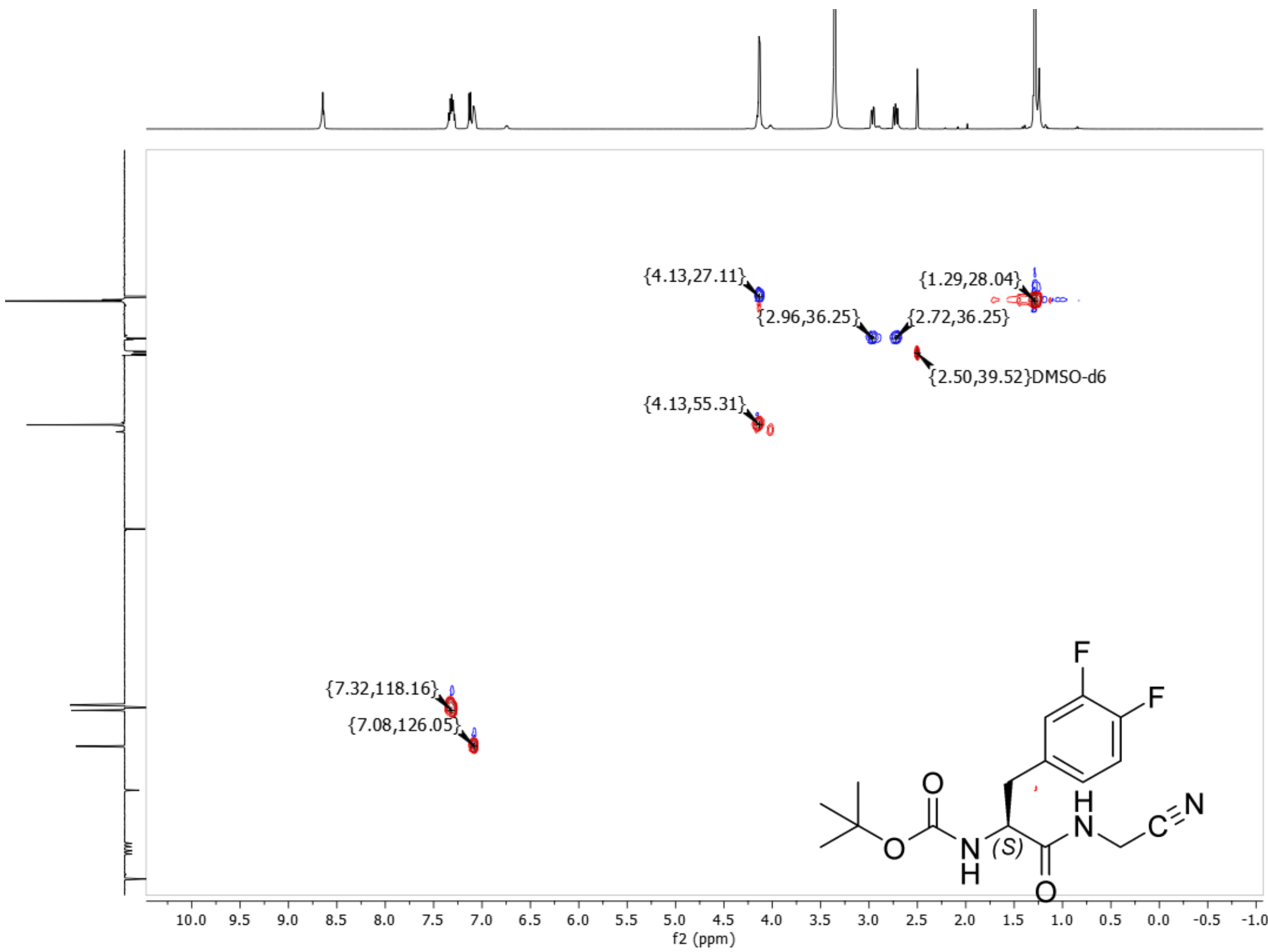

Figure S29. HSQC-NMR Spectrum of compound **5** (150 MHz DMSO-*d*6).

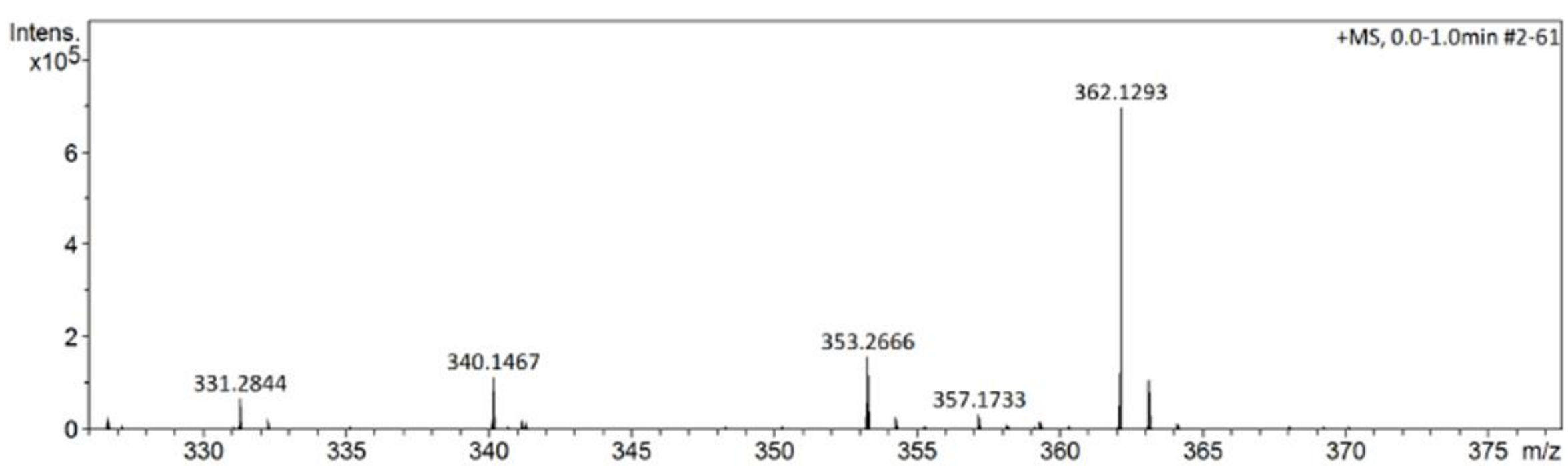

Figure S30. HRMS Spectrum of compound **5** (ESI$^+$).

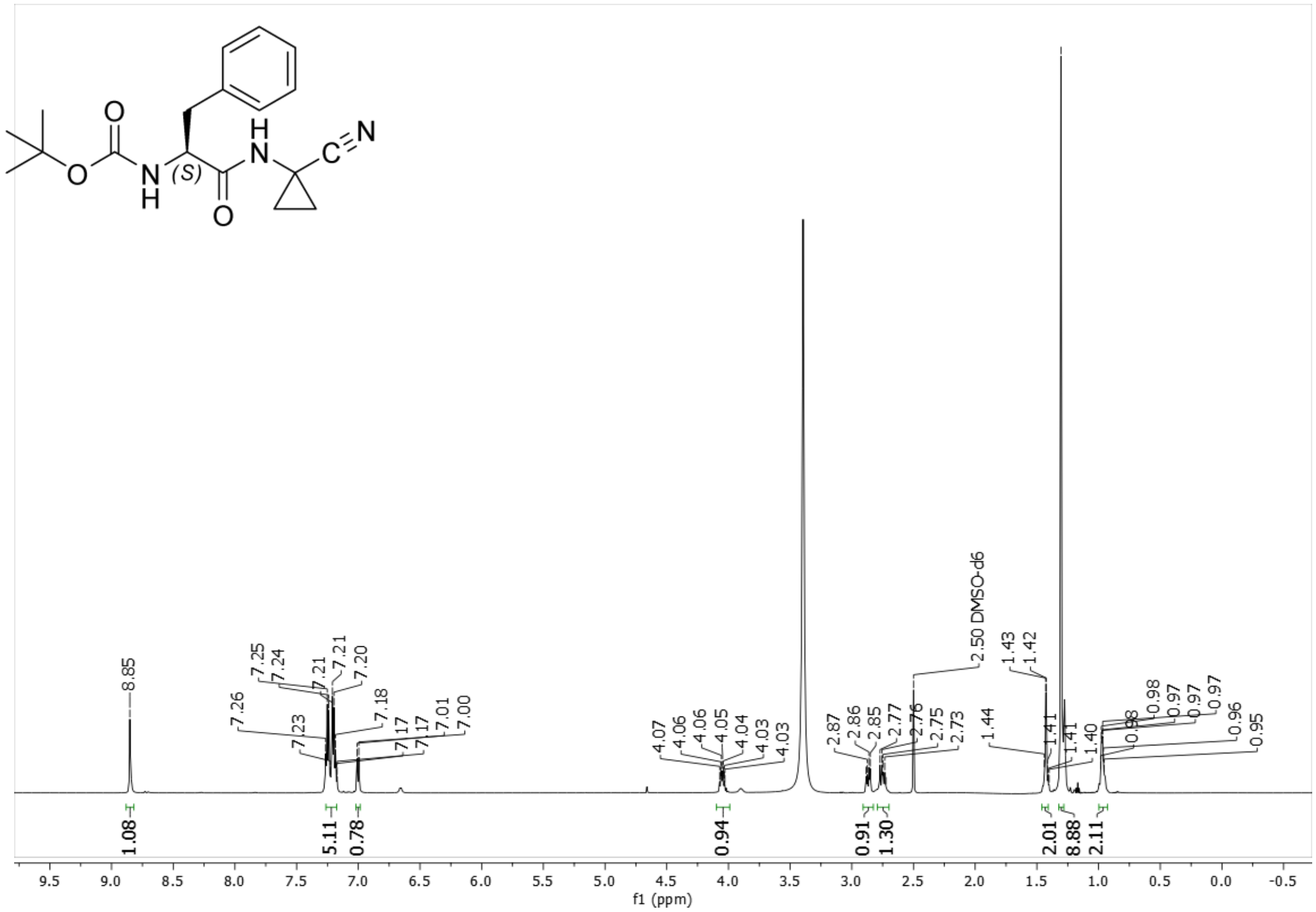

Figure S31. ^{1}H-NMR Spectrum of compound **6** (600 MHz DMSO-*d*6).

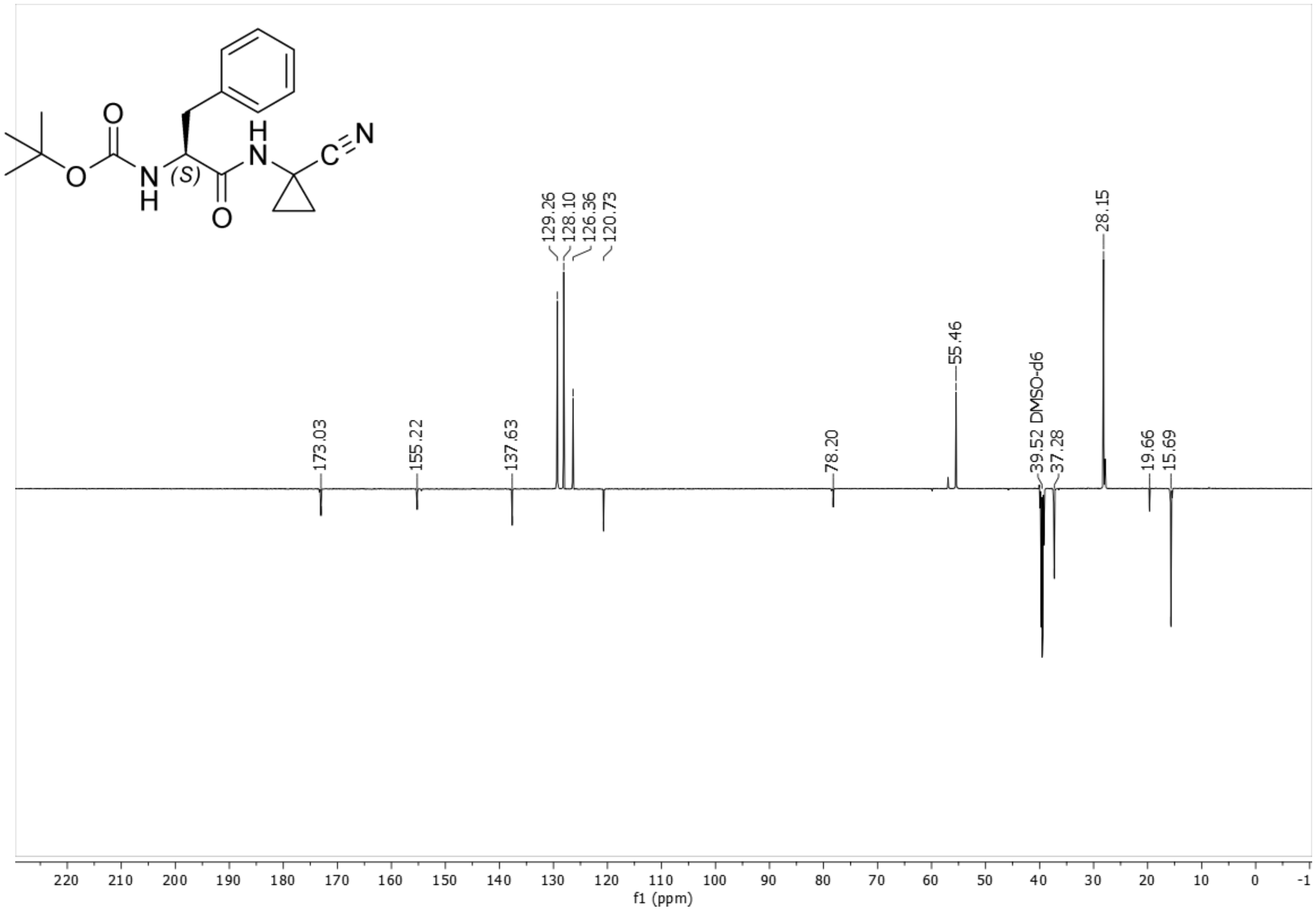

Figure S32. DEPTQ-NMR Spectrum of compound **6** (150 MHz DMSO-*d*6).

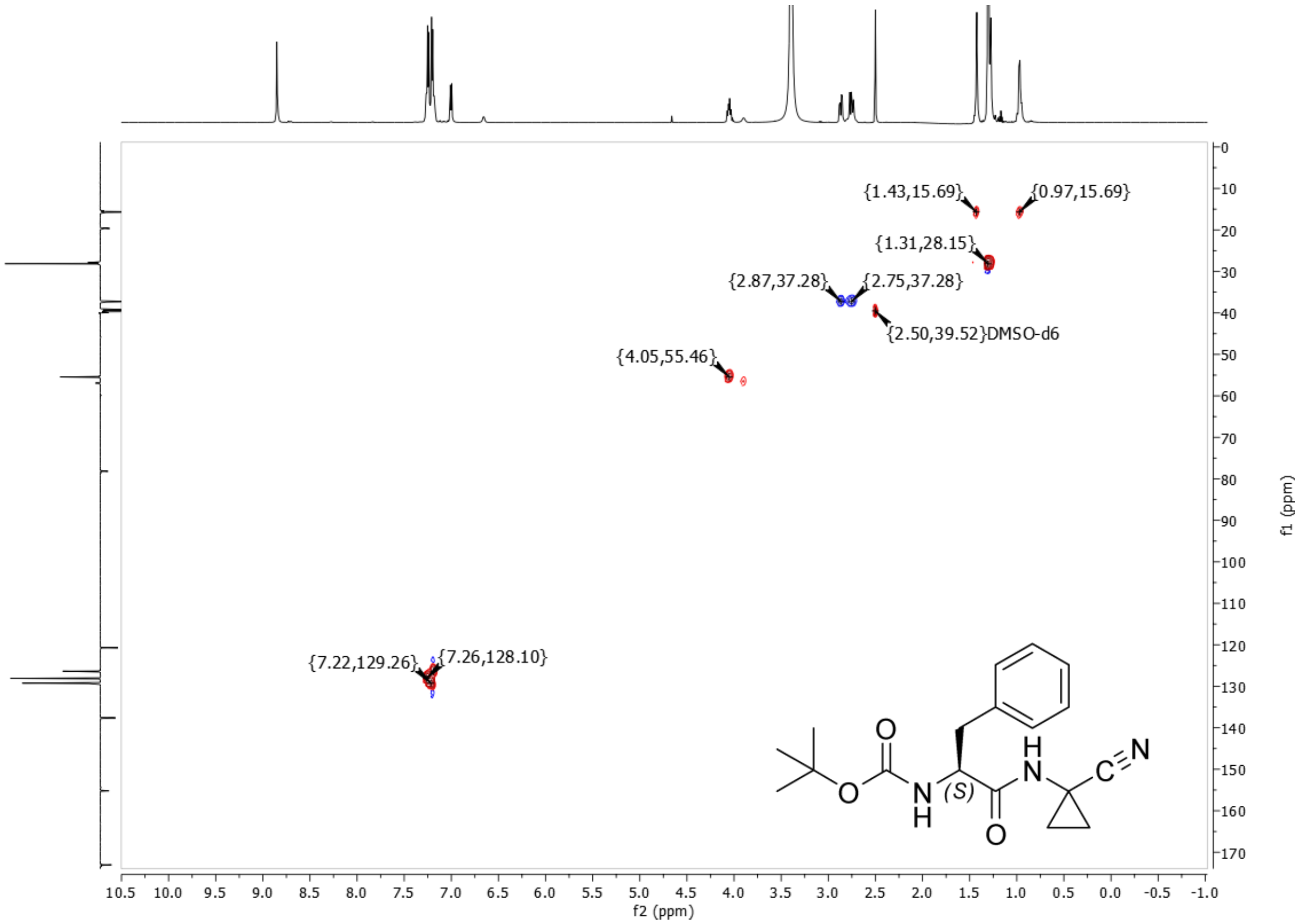

Figure S33. HSQC-NMR Spectrum of compound **6** (150 MHz DMSO-*d*6).

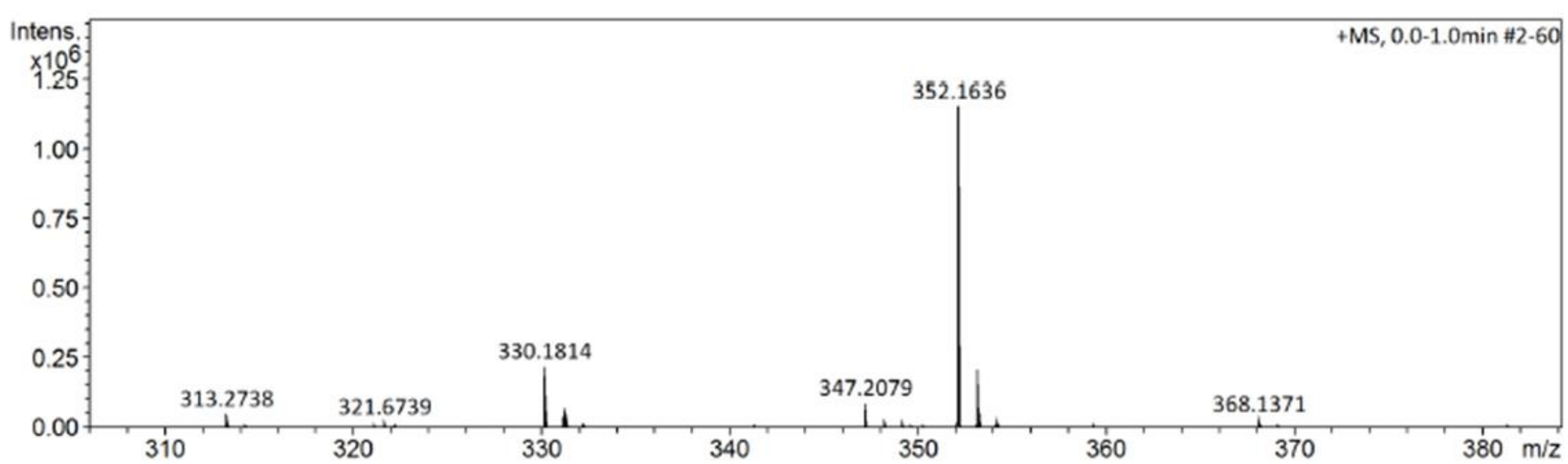

Figure S34. HRMS Spectrum of compound **6** (ESI$^+$).

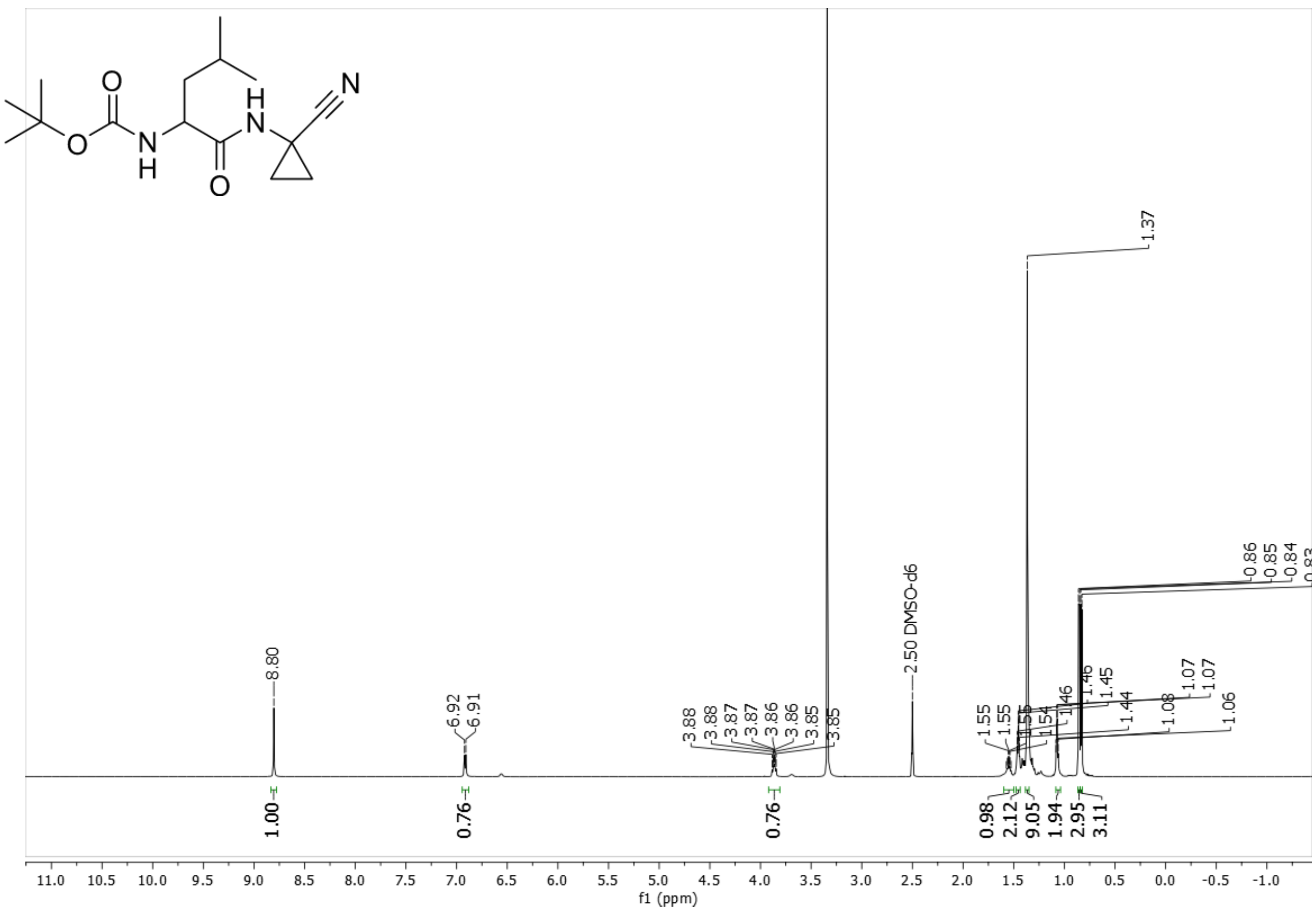

Figure S35. ^{1}H-NMR Spectrum of compound **7** (600 MHz DMSO-*d*6).

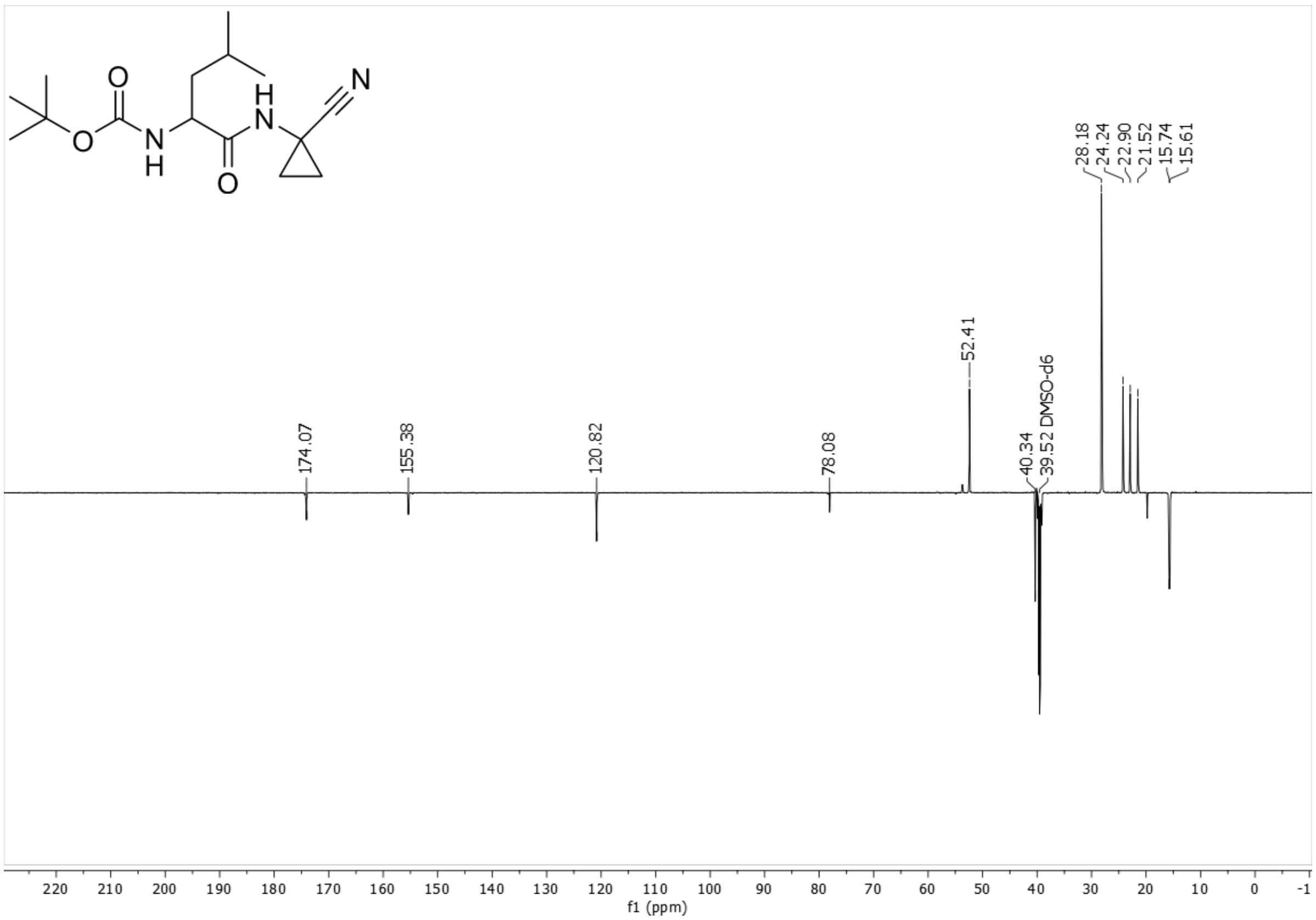

Figure S36. DEPTQ-NMR Spectrum of compound **7** (150 MHz DMSO-*d*6).

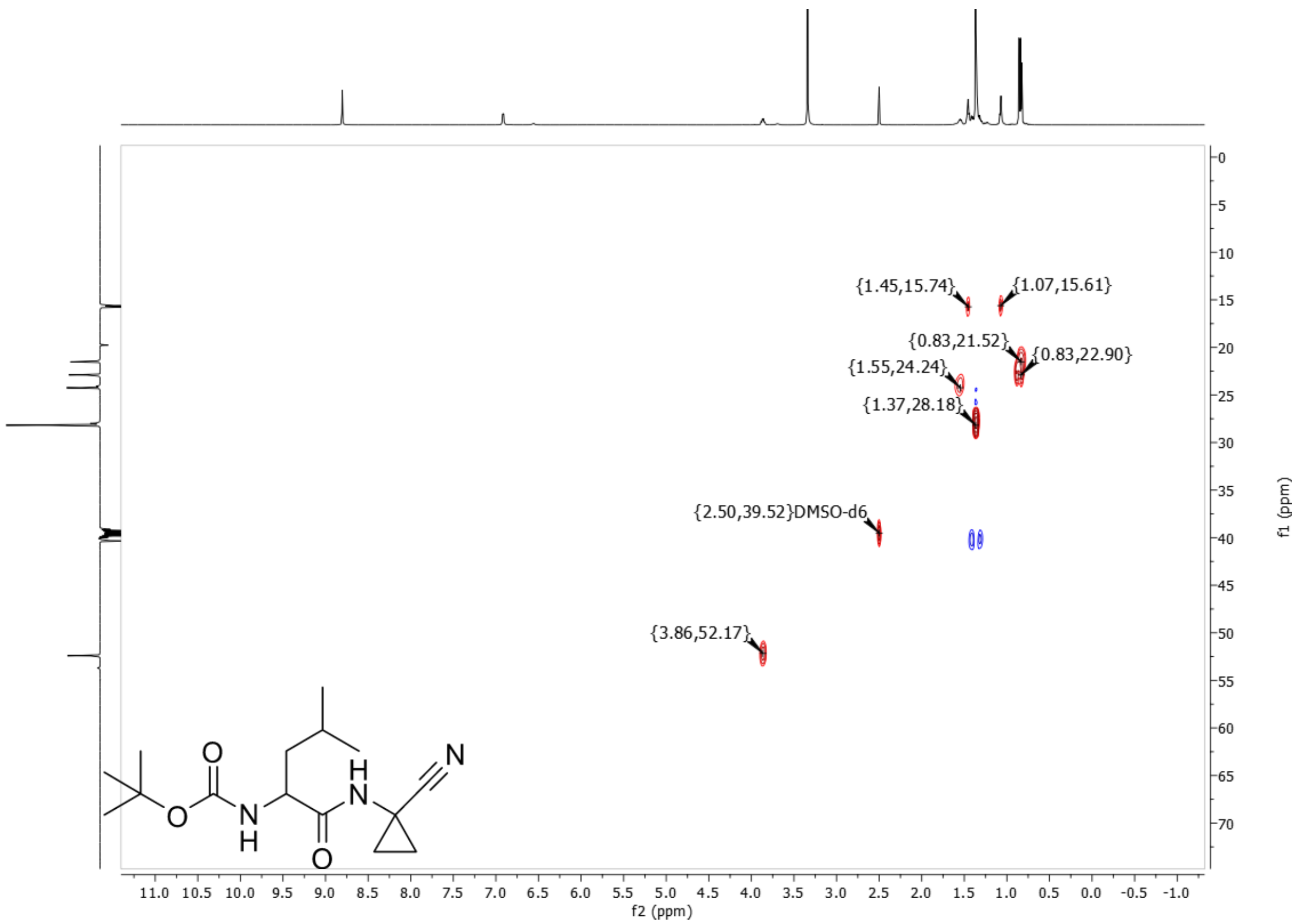

Figure S37. HSQC-NMR Spectrum of compound **7** (150 MHz DMSO-*d*6).

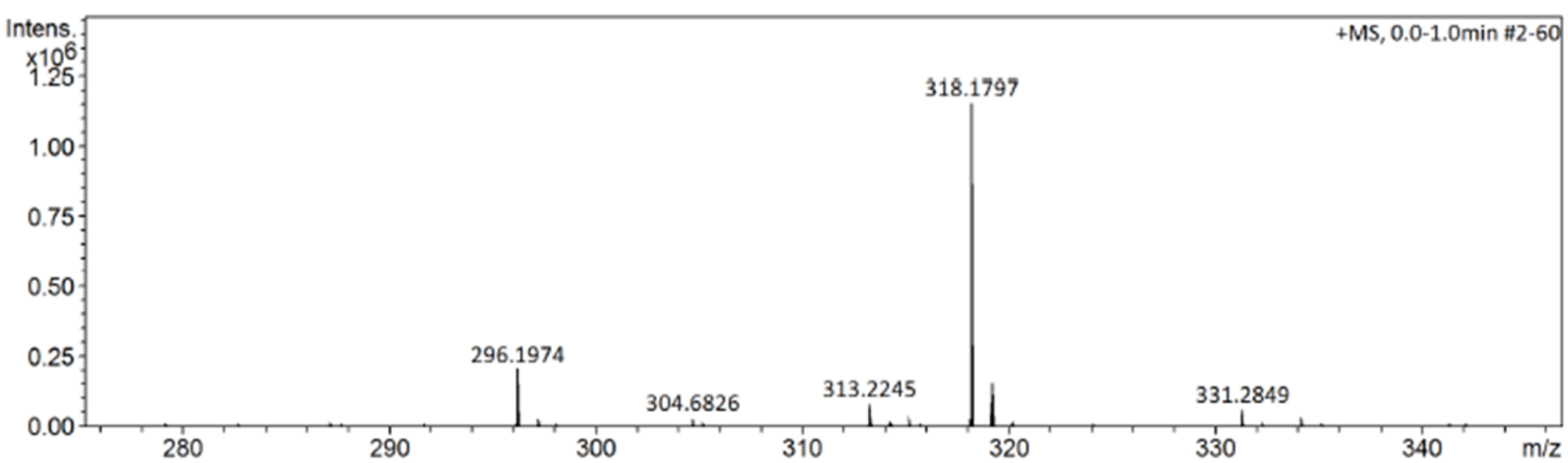

Figure S38. HRMS Spectrum of compound **7** (ESI$^+$).

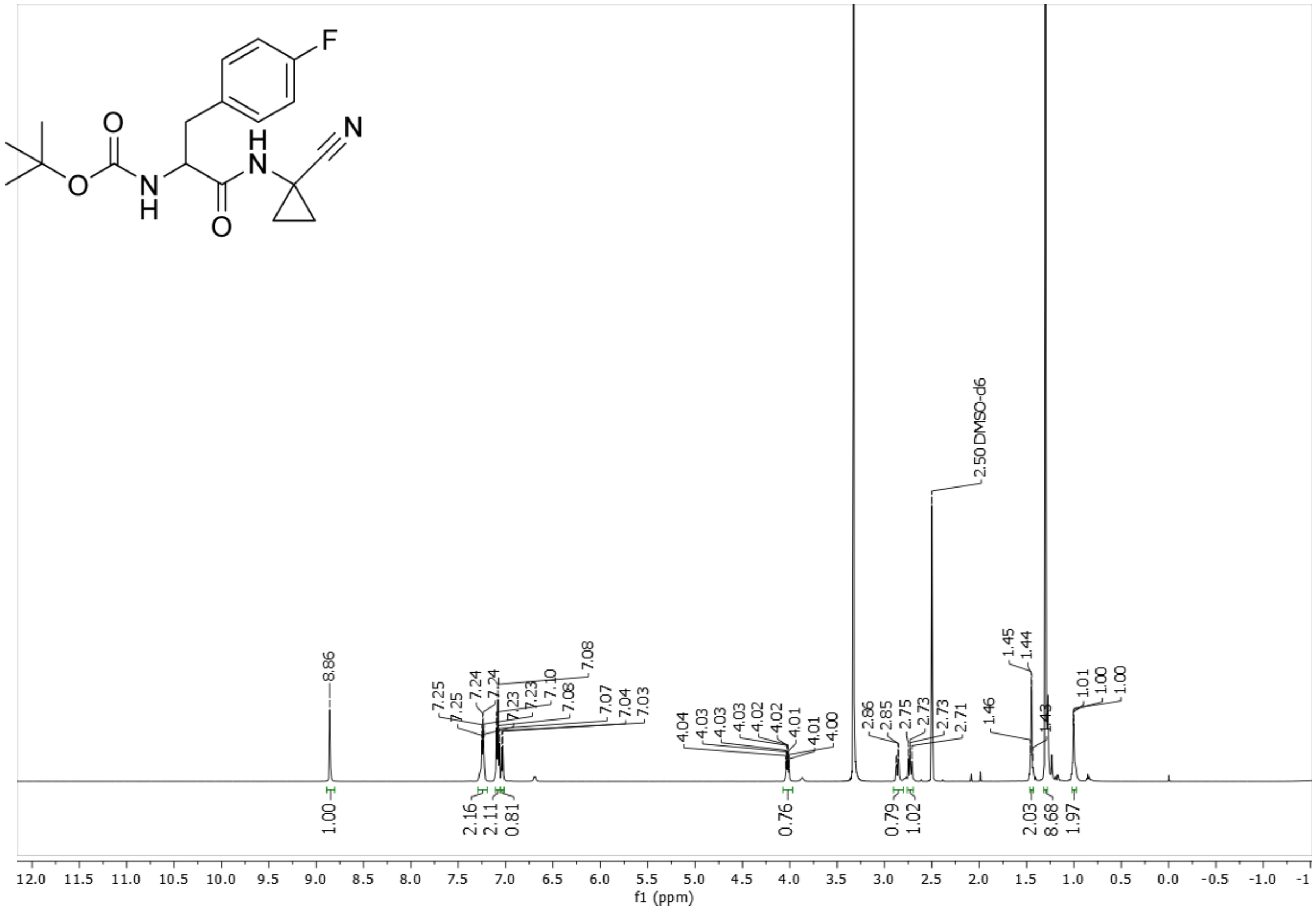

Figure S39. ^{1}H-NMR Spectrum of compound **8** (600 MHz DMSO-*d*6).

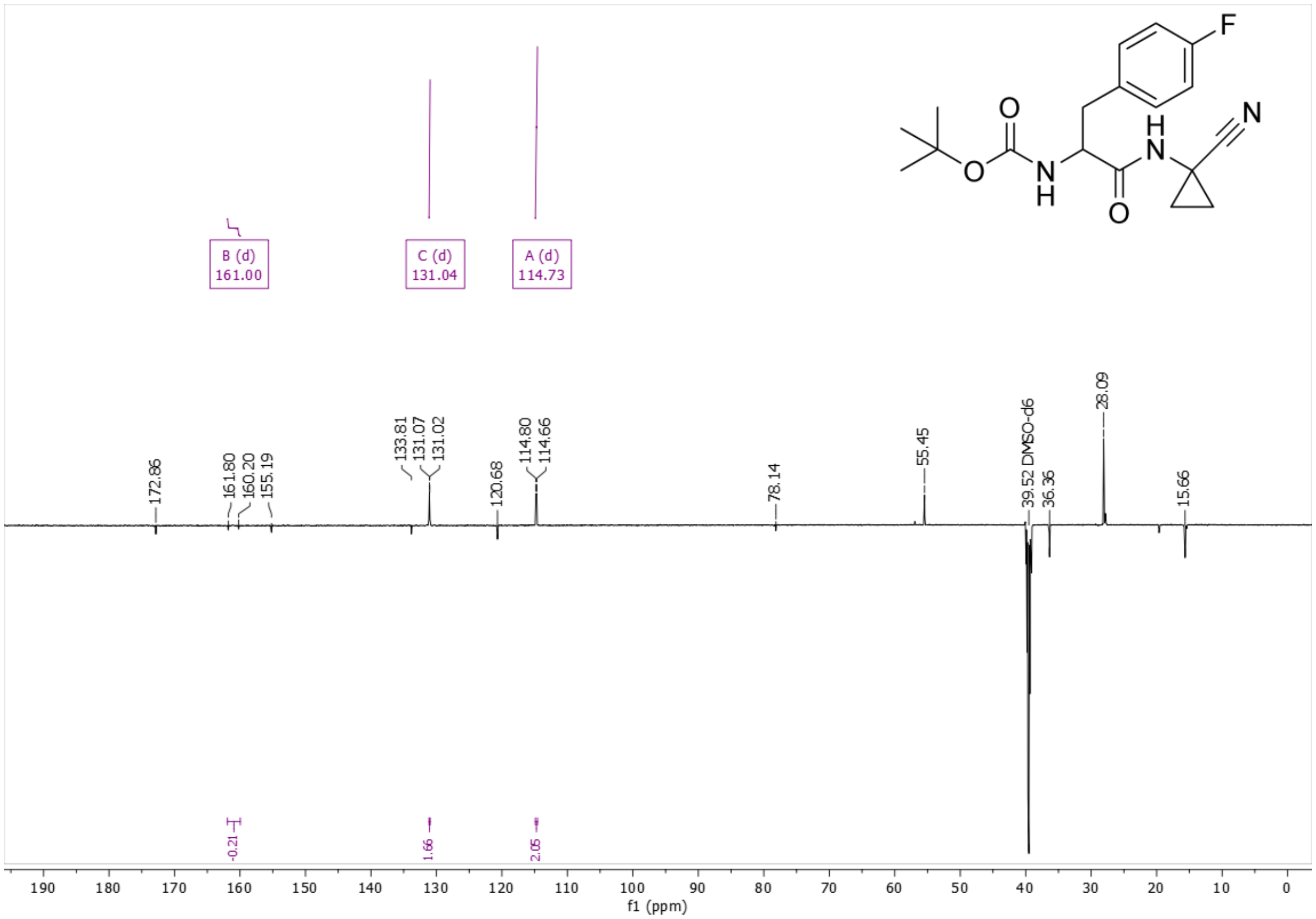

Figure S40. DEPTQ-NMR Spectrum of compound **8** (150 MHz DMSO-*d*6).

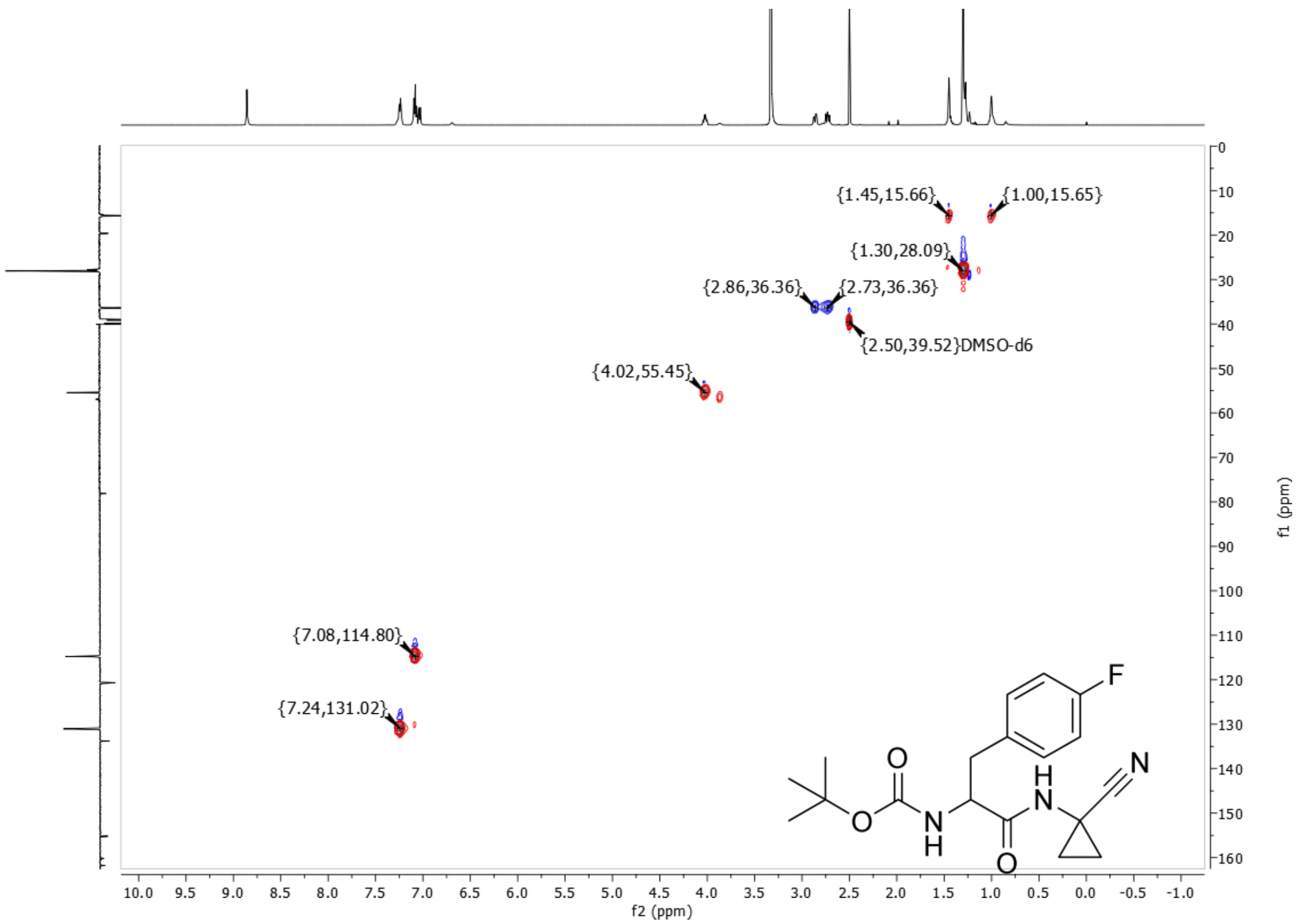

Figure S41. HSQC-NMR Spectrum of compound **8** (150 MHz DMSO-*d*6).

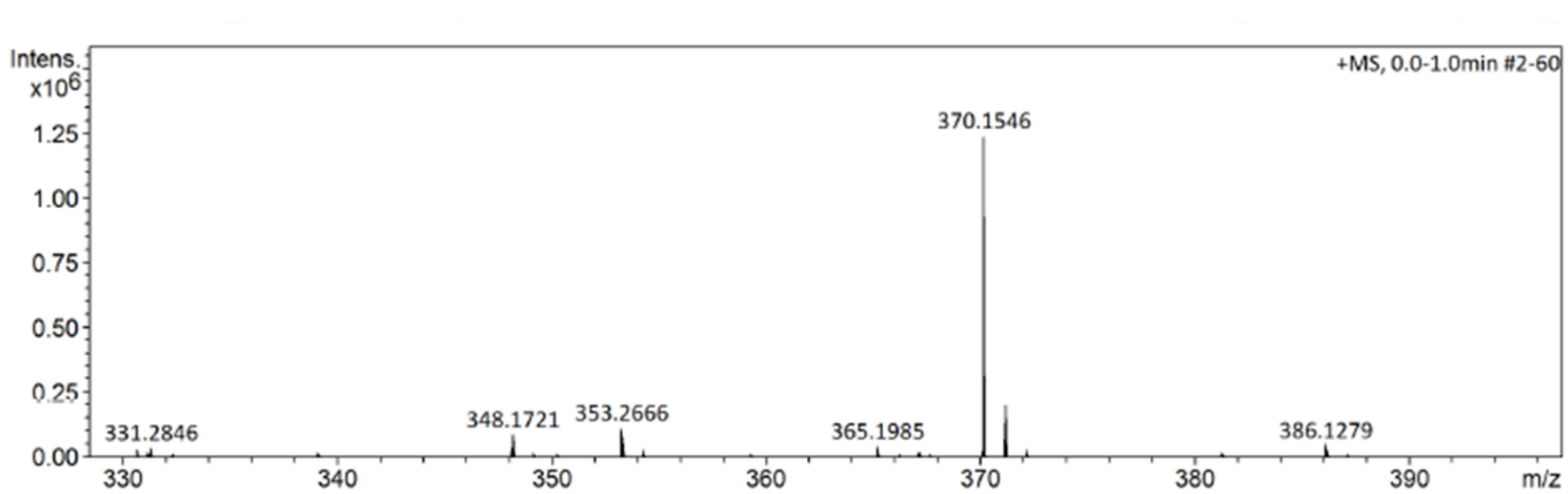

Figure S42. HRMS Spectrum of compound **8** (ESI$^+$).

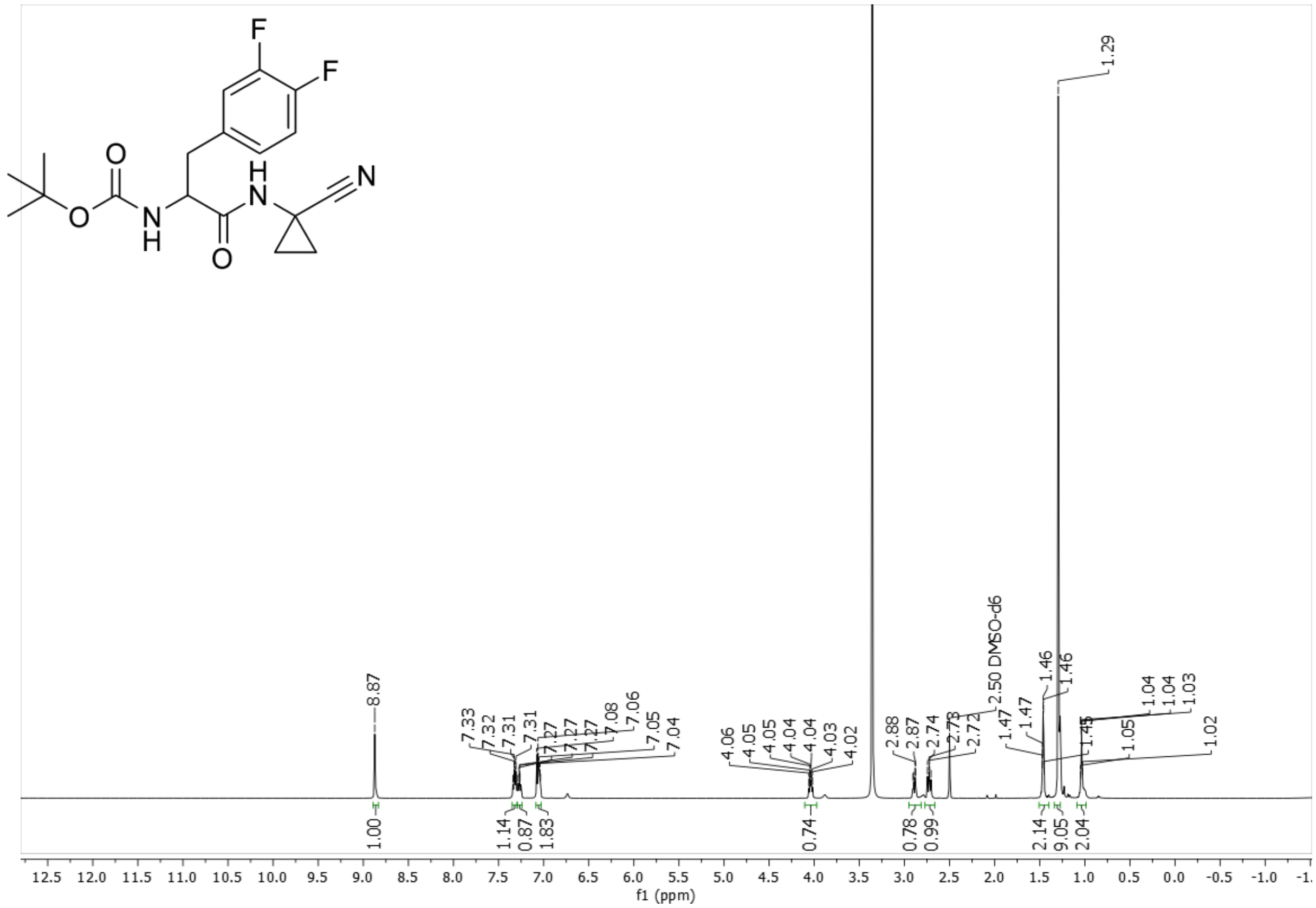

Figure S43. ^{1}H-NMR Spectrum of compound **9** (600 MHz DMSO-*d*6).

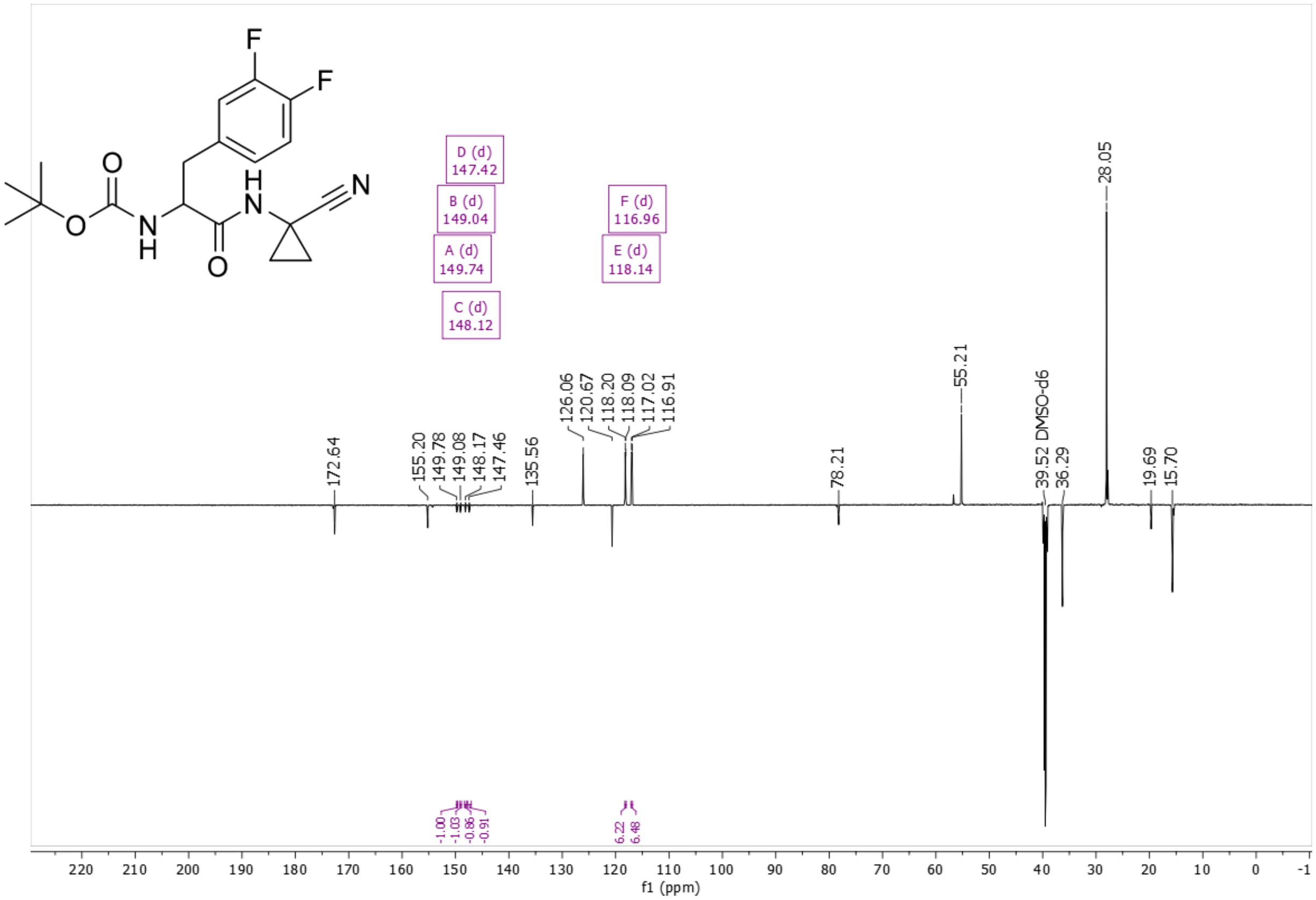

Figure S44. DEPTQ-NMR Spectrum of compound **9** (150 MHz DMSO-*d*6).

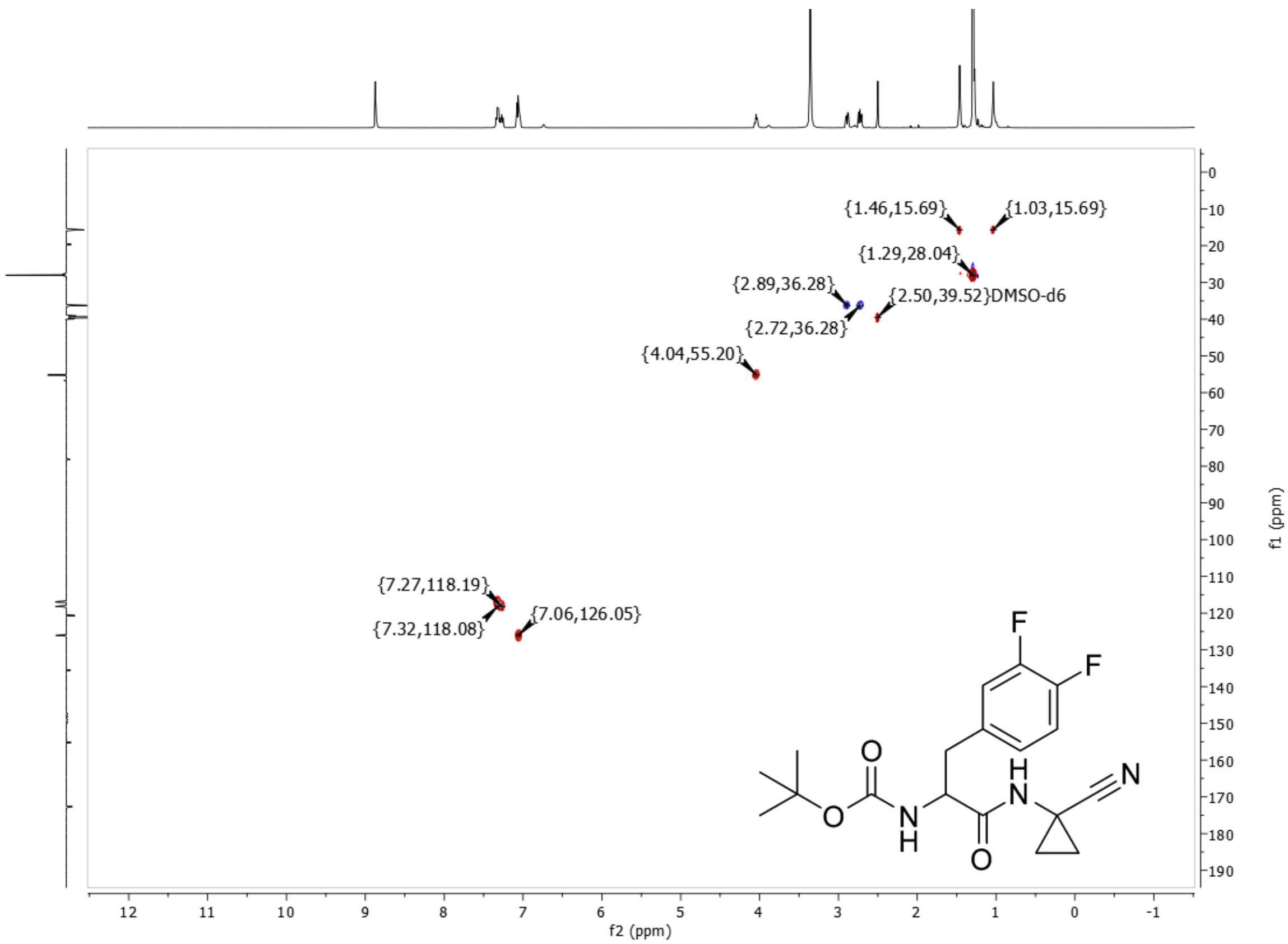

Figure S45. HSQC-NMR Spectrum of compound **9** (150 MHz DMSO-*d*6).

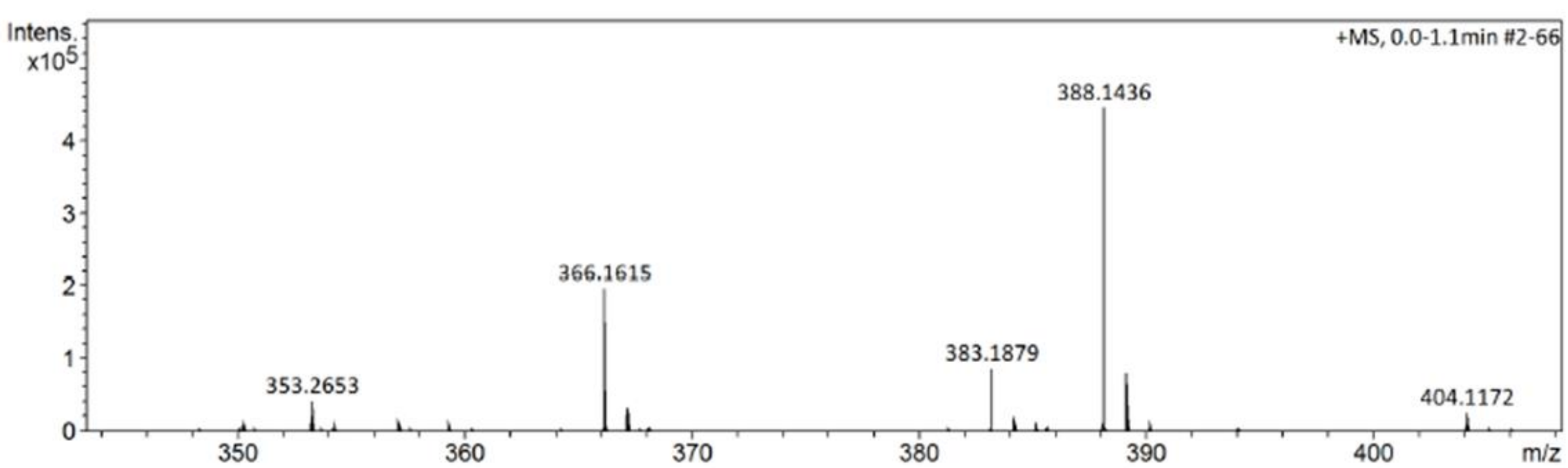

Figure S46. HRMS Spectrum of compound **9** (ESI$^+$).
